# Field Evaluation of Do-It-Yourself Air Filtration Solutions for Evaporative Coolers to Reduce Ambient Particle Infiltration in Homes in Wildfire-Affected Communities

**DOI:** 10.1021/acsestair.5c00488

**Published:** 2026-03-10

**Authors:** Mingyu Wang, Aditya Singh, David Chang, Isabella Kaser, Jeff Wagner, Zhong-Min Wang, Brett C. Singer, Shelly L. Miller, Nayamin Martinez, Ruben Rodriguez, Stephanie Jarmul, McKenna Thompson, Peggy Reynolds, Julie Von Behren, John R. Balmes, Mohammad Heidarinejad, Brent Stephens, Gina Solomon

**Affiliations:** † Department of Civil, Architectural, and Environmental Engineering, 2455 Illinois Institute of Technology, Chicago, Illinois 60616, United States; ‡ Tracking California, 1665 Public Health Institute, Oakland, California 94607, United States; § Center for Laboratory Sciences, Environmental Health Laboratory, 117025 California Department of Public Health, Richmond, California 94804, United States; ∥ 1666 Lawrence Berkley National Laboratory, Berkeley, California 94720, United States; ⊥ Department of Mechanical Engineering, 1877 University of Colorado, Boulder, Colorado 80309, United States; # Central California Environmental Justice Network, Fresno, California 93727, United States; ¶ Office of Environmental Health Hazard Assessment, 7020 California Environmental Protection Agency, Oakland, California 94612, United States; ∇ Department of Epidemiology and Biostatistics, University of California, San Francisco, California 94158, United States; ○ School of Medicine, University of California, San Francisco, California 94143, United States; ⧫ Division of Environmental Health Sciences, School of Public Health, 43166 University of California, Berkeley, California 94704, United States

**Keywords:** air pollution, air filtration, indoor air quality, particulate matter, wildfire, low-cost sensors, air quality intervention

## Abstract

Evaporative coolers (ECs) introduce outdoor air pollutants indoors when operating. This study evaluates the potential of do-it-yourself (DIY) air filtration solutions for ECs to cost-effectively reduce the infiltration of ambient fine particulate matter (PM_2.5_) in homes with ECs using measurements in 48 homes in wildfire-affected agricultural communities in California. All homes received one portable air cleaner (PAC); 25 homes also received DIY filters (mostly MERV 13) attached to their ECs. PurpleAir monitors measured indoor and outdoor PM_2.5_ concentrations. PAC operation was monitored in all of the homes. EC usage was monitored in some homes and predicted using relative humidity dynamics in all homes. Conditional analyses between EC likely on and off conditions were used to evaluate the impacts of DIY EC filters on ambient PM_2.5_ infiltration, including during several wildfire-affected days. Median levels of ambient PM_2.5_ infiltration increased ∼36–42% in homes with only PAC interventions when ECs were likely operating compared to only ∼10–11% in homes with both PACs and DIY EC filters. Pre/postintervention comparisons in a subset of homes confirmed PM_2.5_ infiltration reductions. EC filter performance declined after extended use. Results suggest that short-term EC filter deployments are likely a cost-effective way to mitigate wildfire smoke infiltration inside these homes.

## Graphical abstract

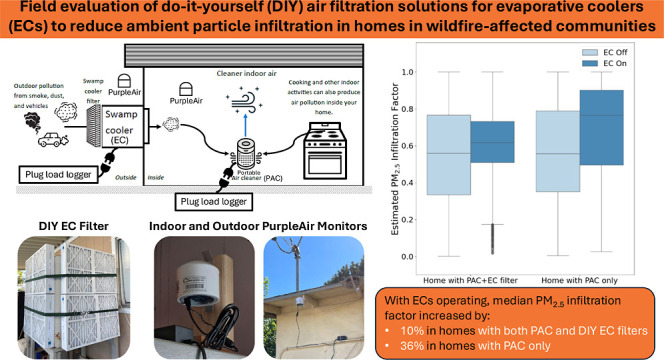

## Introduction

In hot and dry regions of the world, many homes use evaporative coolers (ECs), also called “swamp coolers”, to cool their living spaces.^1^ Because ECs draw large amounts of outdoor air indoors through moist pads with low filtration efficiency for particles and gases,^2,3^ their operation can increase the infiltration of ambient air pollutants, including pollutants in wildfire smoke in affected regions.^4,5^ Infiltration of wildfire smoke through ECs is particularly important given the increasing frequency and severity of wildfires and the adverse impacts of wildfire smoke exposure on human health.^6–16^ The increasingly frequent co-occurrences of both wildfire smoke and extreme heat,^17,18^ during which indoor cooling is a necessity, further motivate the need to address pollutant infiltration through ECs during wildfire events. Among the many pollutants commonly present in wildfire smoke, including particulate matter (PM) and its constituents (e.g., organic carbon and trace metals), gases (e.g., carbon monoxide (CO), nitrogen oxides (NO_*x*_), and volatile organic compounds (VOCs)), and both gas-phase and particle-bound semivolatile organic compounds (SVOCs) such as polycyclic aromatic hydrocarbons (PAHs),^19–23^ the adverse health effects associated with wildfire smoke exposure to date are largely attributed to fine particulate matter (PM_2.5_) exposure.^24,25^ Because people spend most of their time indoors^26^ where ambient PM_2.5_ infiltrates and persists with varying efficiencies,^27–37^ much of human exposure to PM_2.5_ from wildfire smoke and other ambient sources occurs indoors, especially at home.

To address these challenges in homes with ECs in regions affected by wildfire smoke, we initially developed and laboratory-tested simple do-it-yourself (DIY) filtration solutions for residential ECs to reduce the level of infiltration of pollutants common in wildfire smoke. Solutions primarily targeted particle removal but also sought some removal of VOCs with the appropriate filter media selection. The temporary solution, which was inspired by DIY box fan and filter combinations as an emerging low-cost wildfire smoke mitigation measure,^38–40^ consists of the direct attachment of particle filters to the outdoor air intakes on the exterior of ECs using bungee straps.^41^ The use of new 10 cm-thick filters with a minimum efficiency reporting value (MERV) of 13 was shown in laboratory testing to achieve at least 40–50% single-pass particle removal efficiency for most particles >0.3 μm in diameter while minimizing airflow resistance and maintaining reasonably high EC flow rates necessary for effective residential cooling.^42^ The use of MERV 13 filters impregnated with activated carbon was also shown to provide some VOC removal in addition to the desired particle removal. Flat sheet filter media with various MERV values were also shown to be effective in supplementing coverage of irregularly shaped ECs, with differing impacts on airflow resistance and particle removal. Although MERV 13 filters in other building applications are effective at mitigating the infiltration of PM_2.5_ in wildfire smoke,^43^ the DIY EC solution had not yet been field-tested in real residences.

Here, we describe an experimental field evaluation of the impact of DIY EC filtration solutions on indoor PM of predominantly outdoor origin and their cost-effectiveness in the homes of predominantly agricultural workers in a wildfire-prone region of California’s San Joaquin Valley, which has the highest prevalence of asthma in the state.^44^ We leverage high time-resolution measurements of indoor and outdoor PM_2.5_ with low-cost particle monitors over multiple months to compare the effects of the deployed DIY filtration solutions to a conventional wildfire smoke mitigation recommendation of operating portable air cleaners (PACs) with high-efficiency particulate air (HEPA) filters indoors. While the motivation for this work was to reduce wildfire smoke exposure, farmworker communities in the region are also impacted by other sources of ambient air pollution including agricultural dust, roadway and off-road equipment emissions, soil-derived *Coccidioides* (causing valley fever), and ambient ozone,^45–49^ for which the developed filtration solutions may also be beneficial but are not explicitly tested here.

## Materials and Methods

This research was conducted as part of the Filtration for Respiratory Exposure to wildfire Smoke from Swamp Cooler Air (FRESSCA) and FRESSCA-Mujeres community-engaged research projects, which aimed to develop and test air filtration solutions for reducing the infiltration of wildfire smoke into the homes of farmworkers. Participants with residential ECs were recruited by the Central California Environmental Justice Network (CCEJN) in three counties in the San Joaquin Valley (Fresno, Kings, and Kern). A smaller cohort of participants was initially recruited for pilot testing in 2022 (details are provided in the Supporting Information), lessons from which were used to inform a larger full-scale intervention study in 2023 with a larger cohort of participants. In both phases, participation was restricted to individuals living in nonsmoking homes to minimize other sources of PM in the study homes. The study was approved by the Public Health Institute IRB #I22-002 and #I22-002a.

### Full-Scale Intervention

In the full-scale intervention testing in 2023, participants in 58 homes were recruited in Fresno, Kings, and Kern Counties. Recruitment began in April 2023 and continued through September 2023. All interventions were deployed between July and September 2023, staggered with recruitment, and left in place along with monitoring instruments into October 2023, when all equipment was retrieved from study homes. Participants in 48 homes ultimately received intervention(s) and completed field measurements. The recruited homes were mostly a mix of detached single-family, manufactured, and mobile homes, with an average floor area of ∼130 m^2^. Each home had either a side-mounted or roof-mounted EC unit (most of which were not ducted), and some also had window air-conditioning (AC) units. Central forced air heating or cooling systems or other heating systems (e.g., woodstoves) were not observed in the homes.

All homes were provided one PAC with a HEPA filter as a conventional wildfire smoke mitigation measure.^50–57^ To the best of our knowledge and observations, none of the participants had already owned air cleaners prior to the study. Homes received either a Levoit 300, Winix D360, or Levoit H133 PAC. The sample of PACs was selected through a combination of availability via solicited donations (Winix) and acquisition following engagement with community partners to identify relatively small-footprint devices with reasonably high clean air delivery rates (CADRs) and low noise levels. PACs were not explicitly sized in each home via calculations but rather were selected and deployed in a convenient location based on practical considerations such as available floor space, power outlet availability, and occupant preferences. The manufacturer-reported CADR for smoke-sized particles (0.09–1 μm) and energy efficiency rating (CADR/W) for the deployed PACs were obtained from the ENERGY STAR Certified Room Air Cleaners list that was available at the time of deployment in 2023,^84^ each reported for the highest fan speed settings as follows: Levoit 300 – 238 m^3^/h, 5.3 (m^3^/h)/W; Winix D360 – 396 m^3^/h, 6.6 (m^3^/h)/W; and Levoit H133 – 483 m^3^/h, 10 (m^3^/h)/W. Tables A1 and A2 in the Supporting Information summarize basic home characteristics and which type of PAC each home received for the double and single intervention homes, respectively.

Twenty-five (25) homes also received DIY filtration solutions on their ECs. In each home that received a DIY EC filtration solution, filters were attached to the exterior air intakes of their ECs.^41,42^ Most of the DIY EC filtration solutions included 10 cm (4 in.)-depth MERV 13 filters attached with bungee straps, with gaps taped to improve the seal as necessary. A mix of 10 cm MERV 13 filters with impregnated carbon (Rensa CarbonWeb CA-13 or AAF International AmAir/C M13 filters) was primarily used, with some 10 cm filters without carbon as backup (Tex-Air MERV 13 commercial pleated filters). The MERV 13 filters all had similar initial size-resolved particle removal efficiency from both ASHRAE Standard 52.2 laboratory tests (per data sheets) and our prior laboratory testing.^42^ While we demonstrated previously via laboratory testing that the carbon-impregnated filters offer some modest removal of VOCs that can be present in wildfire smoke,^42^ VOCs were not assessed in this part of the field study. Only two common sizes of 10 cm (4 in.) depth MERV 13 filters were used to standardize sizing in the field deployments: either 41 × 63 cm (16 × 25 in.) or 51 × 76 cm (20 × 30 in.). As many filters as necessary to cover the entire EC air intake areas were used, typically ranging from 2 to 6 filters, depending on the EC size and filter dimensions. Some homes also received supplemental flat-sheet (0.5 cm thick) filter media to cover narrow or curved portions of EC intakes. Flat-sheet media products, which were purchased via major online retailers and had various brand names, were labeled as either MERV 13 or 16, whereas we previously lab-tested them to perform closer to MERV 11 when new. In two homes with obstructions around their EC intakes, only flat-sheet filter media was used to cover the entire EC intake area. Table A3 in the Supporting Information summarizes filter deployment details for each home. Several photographic examples of DIY EC filter installations are also shown in the Supporting Information Appendix.

The DIY EC filtration solutions were not installed on ECs that were located on rooftops for access and safety reasons; thus, many of the homes that received only indoor HEPA-based PACs had rooftop EC units. Therefore, while true randomization was desired, the pragmatic study design instead resulted in pseudorandomization, whereby selection into the EC filtration group was determined in large part by the team’s ability to access the EC and install the DIY filtration solution. However, visual inspections suggested that there was a similar distribution of EC makes and models across rooftop ECs and both through-the-wall and through-the-window (i.e., horizontal flow) ECs. Once installed, the DIY EC filtration solutions were left attached for as long as ∼3 months, representing a relatively long-term use of the solution compared to their intended design use for short-term deployments only during wildfire conditions. Yet, the longer-term deployments allowed for the collection of greater amounts of data during both nonwildfire and, if they occurred, wildfire conditions, which are episodic and can be rare, and also allowed for investigating potential changes in filtration performance over time. All participants were provided with free professional EC servicing prior to filter installation to ensure that their equipment was working properly.

### Long-Term Measurements

Each home received a PurpleAir (PA) PA-II with Wi-Fi connectivity or, most commonly, a PA-II-SD monitor with onboard microSD card storage in addition to Wi-Fi connectivity to measure indoor PM concentrations at high-time resolution throughout the study. PA monitors (PAs) were purchased new for this project and were chosen because of their good performance against research- and regulatory-grade monitors^58^ and their minimal drift in instrument response over time scales relevant to this project.^59–61^ PAs were placed away from PACs where possible; however, in some homes, limited space and access to power outlets prevented ideal placement, such that in some cases, the devices were placed next to each other. While not ideal, these rooms were relatively small and likely reasonably well mixed. A total of 8 outdoor PAs were also installed in locations near clusters of homes such that multiple homes could be represented by a single outdoor monitor. The distance between each home and its nearest outdoor PA is provided in Tables A1 and A2 in the Supporting Information appendix. Distances ranged from 0 to 14.5 km with a mean (SD) of 1.3 (2.8) km and a median of 0.3 km; most homes (>90%) had their nearest outdoor PA located within 2.5 km.

Some homes, depending on recruitment timing and availability, received PAs before the installation of PAC/EC intervention(s) (as early as April 2023), while others received PAs only during installation of the intervention(s) (between July and September 2023, depending on the rolling recruitment date). All PAs that were connected to Wi-Fi transmitted data at 10 min intervals. PAs with onboard SD cards also logged data at 2 min intervals for backup. Team members routinely monitored the PAs that were connected to Wi-Fi to ensure they were online and transmitting data. If monitors were offline for more than ∼7 days, they were reported to the CCEJN field team for further troubleshooting. However, it was not always possible to bring monitors back online in a timely manner. Thus, it was common to rely on backup onboard SD data.

The use of PACs and ECs was not prescribed but instead was determined by participant preferences and behaviors. After learning from the pilot year testing that we had difficulties knowing when PACs or ECs were operating without direct monitoring, Onset HOBO Plug Load Data Loggers were installed on all PACs and as many of the ECs as feasible to monitor their operation during the full-scale intervention testing in 2023.^62^ Plug load loggers (PLLs) logged continuously at 1 min intervals. PLLs were not installed on most of the rooftop ECs because access was restricted, and power was not typically delivered through accessible outlets (i.e., they were hardwired).

### Spot Measurements

Spot measurements were conducted following scripted protocols in as many homes as feasible to compare EC performance immediately before and after DIY filter installations. Air velocity leaving the EC supply outlets was measured in a several-point grid pattern (typically 12-points) by using an Extech AN200 vane anemometer. The EC supply airflow rate was estimated by multiplying the average velocity by the area of the supply outlet measured in the field. The pressure difference across the building envelope under different EC operating conditions was measured by using an Energy Conservatory DG-700 differential pressure gauge. The single-pass size-resolved particle removal efficiency across the ECs (and any filters) was estimated by alternately measuring particle concentrations in front of the EC supply outlets and in front of the EC air intakes with a MetOne GT-526 optical particle counter. Spot measurements were also repeated on as many homes as accessible at the time of equipment retrieval in October 2023 to assess the impacts of long-term real-world use on EC filter performance. Individual home readings from spot measurements are shown in the Supporting Information appendix. Field measurement protocols and instructional videos are available on a public repository for the project: https://osf.io/arz3d/overview.

### Data Analysis

With this pragmatic, community-driven study design in which both groups received at least one intervention, there was no true control arm. Instead, we sought to compare results from homes with double interventions (i.e., PAC and DIY EC filtration) to those with only single interventions (i.e., PAC only), similar to a superiority trial design. At least some data were successfully collected from a total of 44 of the 48 recruited homes, including 21 homes with only a PAC and 23 homes with both PAC and DIY EC filters. The distribution of successful data collection among intervention groups and periods is shown in Table S1. A time-stamp-merged master time-series data file was generated to append all available time-resolved field data from all instruments from all homes into one data set for analysis, with the data set for each home beginning with the time of indoor PA deployment and ending with the time that the field team began retrieving equipment from all sites (Oct 3, 2023). Data from the PAs and PLLs were collected and averaged to 10 min intervals. Data from the nearest identified outdoor PAs were merged to these same 10 min intervals for all homes. Laboratory colocation measurements of PAs were conducted at the end of field deployments, and resulting colocation factors were applied to the PA data to “calibrate” monitor data relative to each other using the mean of all monitors’ output as a reference; full details of these colocation procedures and our findings are provided elsewhere.^61^ Data from the PLLs installed on the PACs were used to quantify PAC runtimes and estimate the amount of smoke-sized particle-free air that was delivered by PACs (i.e., the approximate in situ CADR) at each recorded time interval by multiplying the measured power draw by the manufacturer-reported energy efficiency rating (i.e., CADR/W) for the PAC used in each home. It should be noted that these estimates are only approximations of in situ performance. Data from the PLLs installed on the ECs were used to estimate the EC runtimes. Patterns of EC power draw data were visually inspected at each home and were used to generate binary on/off indicators for each measured EC.

These merged data were then used to conduct a variety of conditional statistical analyses based on several logically flagged variables, including intervention type (PAC only vs PAC + DIY EC filter), EC run mode (on vs off), PAC run mode (on vs off, or binned amount of particle-free air delivered), and different periods of time (pre- vs postintervention, during wildfire vs nonwildfire periods, and during either the first few weeks or last few weeks of filter installations). The use of long-term, high-time-resolution data collected in this project was essential to compare PM_2.5_ measurements under a variety of transient conditions with high statistical confidence. This type of nuanced analysis is nearly impossible with traditional integrated samples, which cannot capture the types of varied transient conditions observed herein. Comparisons in each conditionally defined group were analyzed using distributional box plots and statistical measures of the effect size. All box plots show median values, interquartile ranges (IQR) from the first to third quartiles (*Q*_1_–*Q*_3_), upper and lower bounds of *Q*_3_ + 1.5 × IQR and *Q*_1_ – 1.5 × IQR, respectively, and outliers outside of the upper and lower bounds. Because of the large sample sizes and the dependent nature of many observations (e.g., repeated and clustered time-series data from the same homes) in most comparisons, Cohen’s *d* was used to quantify the effect size of differences between two comparison groups^63^ rather than conventional hypothesis testing. While Cohen’s *d* values of 0.2, 0.5, and 0.8 have been commonly interpreted as indicative of small, medium, and large effect sizes, interpretations can vary between fields;^64,65^ thus, we primarily rely on relative comparisons of Cohen’s *d* values herein.

### Constrained Indoor/Outdoor PM_2.5_ Ratios and Infiltration Factors (*F*_inf_)

PM_2.5_ mass concentrations for all time-series data from all indoor and outdoor PAs were calculated using the alternative algorithm with a calibration factor of 3 (ALT-CF3) that was available at the time of data retrieval (i.e., PM_2.5__alt).^66,67^ This algorithm has better accuracy and a lower limit of detection (LOD) compared to other algorithms historically used by PurpleAir.^68,69^ PM concentration data that was downloaded via Wi-Fi by API calls had the PM_2.5__alt algorithm already built in at 10 min intervals. Data downloaded from the microSD cards required postprocessing of raw counts at 2 min intervals into PM_2.5__alt concentrations at 10 min intervals to match the Wi-Fi collected data.^61^ Concurrent indoor and outdoor PM_2.5__alt concentrations in the merged data file were then used to calculate indoor/outdoor (I/O) ratios at all 10 min interval timestamps for which data were available. For concurrent timestamps that were missing the nearest outdoor PA data, a next-nearest and next–next-nearest monitor were also identified, and those data were substituted as the nearest monitor. This approach yields concurrent I/O data without any time lag between outdoors and indoors. Because other studies have reported lag times between outdoor and indoor PM_2.5_ concentrations as high as 2 h,^70,71^ due to a combination of ambient transport dynamics over the distance between outdoor monitors and individual buildings and the time scales of outdoor-to-indoor transport dynamics via infiltration/ventilation, the sensitivity of different lag time assumptions is explored in the Supporting Information.

To narrow comparisons to periods in which indoor PM_2.5_ was likely predominantly of ambient origin, analysis of I/O concentration data is constrained only to periods in which PM_2.5_ I/O ratios are less than or equal to 1, with the logic that for many episodic indoor sources during periods of low-to-moderate outdoor concentrations, I/O ratios are often much greater than 1, and therefore, when I/O is less than or equal to 1, indoor PM is likely predominantly of outdoor origin.^68^ The sensitivity of this approach to different thresholds of constrained I/O ratios (i.e., 0.9 and 1.1) is explored in Supporting Information. Since this approach likely excludes only relatively large indoor sources and may not catch minor indoor PM impacts that result in indoor concentrations that are lower than outdoor concentrations, PM_2.5_ infiltration factors (*F*_inf_) were also estimated from time-series data by algorithmically identifying and excluding periods influenced by intermittent indoor PM sources based on the concept of z-score, as described in the Supporting Information (see Figure S2). Constrained I/O ratios (I/O ≤ 1) and *F*_inf_ values were thus used as the two main indoor PM_2.5_ metrics for evaluating the impacts of DIY EC filtration, since a reduction in indoor PM_2.5_ of outdoor origin is the primary goal of EC filtration and EC filtration is not expected to have any effect on PM_2.5_ from indoor sources, given the location of installation on EC outdoor air intakes.

### Predicting EC Runtime

Because the PLLs were most commonly installed on ECs in homes that received the DIY EC filter solutions (i.e., the same access issues that prevented installation of DIY EC filters on rooftop EC units also prevented installation of plug load loggers on those ECs), measurements of EC operation were not randomized among the single- and double-intervention groups. Among the 44 homes that yielded data for analysis, 16 homes (36%) did not have PLLs installed on the EC (all of which were rooftop-mounted ECs). Therefore, we also developed a method of predicting EC run mode using indoor relative humidity (RH) dynamics measured by the indoor PAs in each home, initially introduced elsewhere^72^ and described in more detail in the Supporting Information (see Figure S3). Briefly, using true run mode data from homes with PLLs installed on their ECs, we found that patterns of rapidly increasing indoor RH could be used to algorithmically predict when ECs were operating. We adjusted algorithm parameters to detect true EC operational periods with a reasonably high accuracy (67%) and a low false positive rate (8%), yielding an additional 10,988 10 min interval data points in which ECs were likely operating to use for analysis in homes that were missing PLL data on their ECs (Table S2). Therefore, we relied on a combination of both measured and predicted values to flag times when ECs are either known or predicted to be operating for use in conditional analyses of indoor and outdoor PM_2.5_ data. We also separately conducted versions of conditional analyses using only measured and predicted EC runtimes to investigate whether the runtime data source influenced the results (reported in the Supporting Information).

## Results and Discussion

### Spot Measurements

Figure 1 shows the percentage change in EC supply airflow rates measured with the DIY filters installed compared to those without filters. In 25 homes that had airflow rate measurements on the day of new filter installation (Figure 1a), the median (IQR) reduction in the flow rate with new filters installed compared to that without filters was 15% (11–23%), which was consistent with prior laboratory testing of these same types of filters.^42^ Approximately 70% of homes met the design goal of less than 20% flow reduction for new EC filter installations. In 20 homes that had paired airflow rate measurements on the day of new filter installation and after approximately 3 months of filter use, the median (IQR) reduction in the flow rate with the filters installed compared to that without filters increased from 13% (9–18%) when filters were new to 17% (10–21%) when filters were used (Figure 1b), demonstrating that particle loading on the filters and other operational factors led to an apparent increase in pressure drop across the filters and a modest reduction in airflow rates, approximately as expected.^73^ No instances of EC fan failure were observed at the conclusion of the field study, suggesting that the attachment of the EC filtration solutions did not present a reliability risk due to fan overheating or overuse in these installations. Smaller flow reductions may be expected for shorter-term use depending on the magnitude of dust loading during operation. The median (IQR) envelope pressure difference from spot measurements was +0.4 (0.3–0.6) Pa with the EC off, +9.2 (4.7–13.1) Pa with the EC on without EC filters, and +5.6 (2.6–7.6) Pa with the EC on and with EC filters installed, suggesting that the homes are positively pressurized with respect to outdoors when ECs are operating both with and without EC filters (Figure S5).

**Figure 1:**
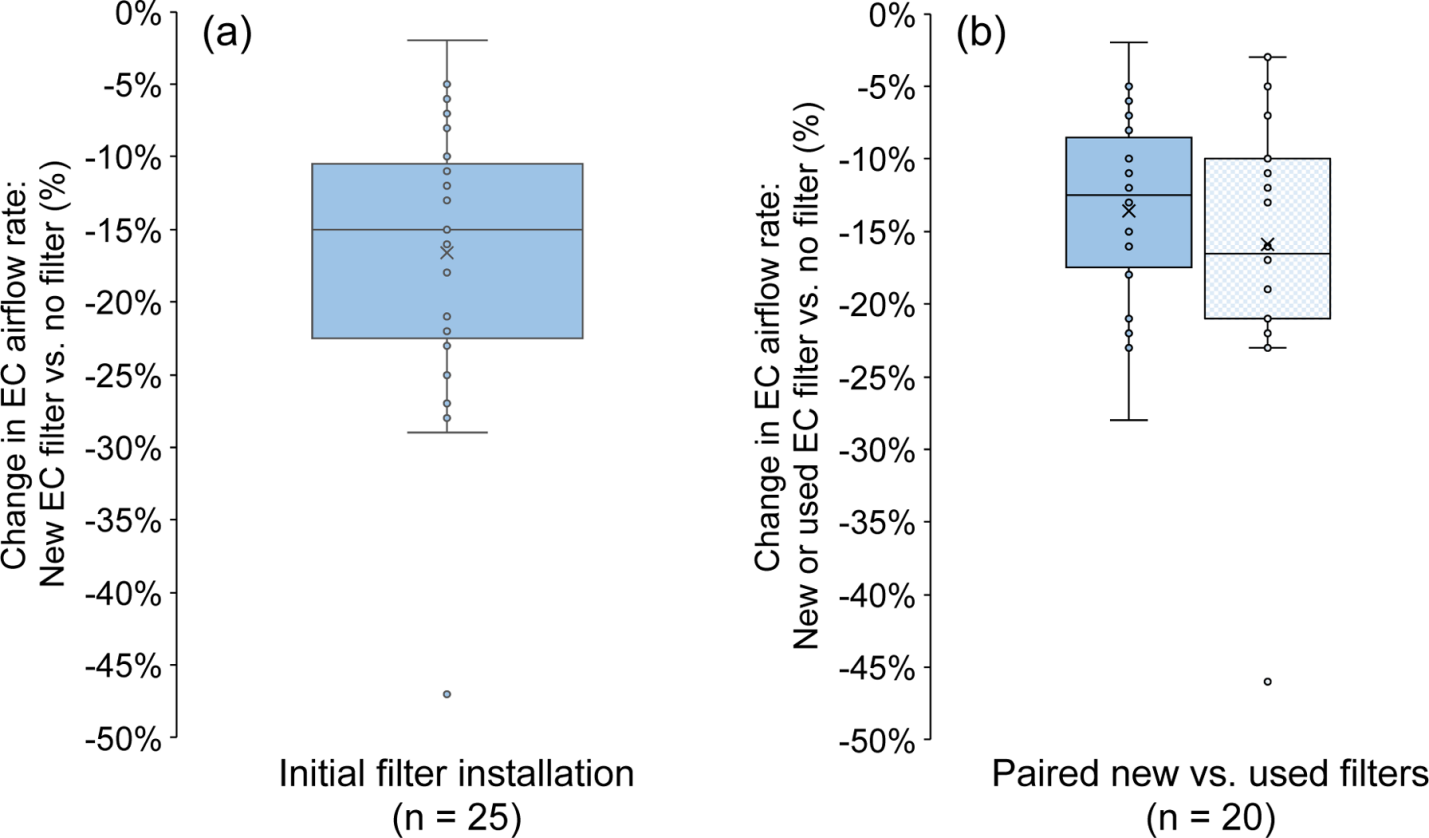
Percentage change in EC supply airflow rates measured with a DIY filter solution installed compared to no filter conditions: (a) following initial filter installation and (b) comparing only those homes that had paired measurements both with new filters and with used filters following approximately 3 months of use.

Figure 2 shows spot measurements of the in situ size-resolved particle removal efficiency of several DIY EC filter installations made (i) immediately after new filters were installed and (ii) after filters were used for approximately 3 months. Measurements were conducted in 13 homes initially and in 11 homes after 3 months of use; 10 homes had paired measurements before and after use. Size-resolved efficiency curves approximately followed expectations for MERV 13 filters, even with the relatively high uncertainty in in situ upstream/downstream concentration measurements without intentional upstream aerosol generation or more controlled aerosol sampling. With new filters, the mean particle removal efficiencies were approximately 49%, 62%, and 76% for particles in the 0.3–1, 1–2.5, and 2.5–10 μm size ranges, respectively; these bins are chosen to approximately match the ASHRAE Standard 52.2 size bins.^74^ After 3 months of use, in situ removal efficiencies declined to approximately 36%, 36%, and 50% for the same corresponding size ranges. This reduction in removal efficiency was likely caused by a combination of decreases in electrostatic charge on the filter fibers after dust loading, impacts of water damage over time, and potentially increased bypass airflow around filters that no longer adhered as securely against the EC intakes as they did at the initial installation. These performance data support the design intent that EC filters should be used primarily in short-term scenarios to mitigate wildfire smoke and then be removed and stored until the next deployment.

**Figure 2:**
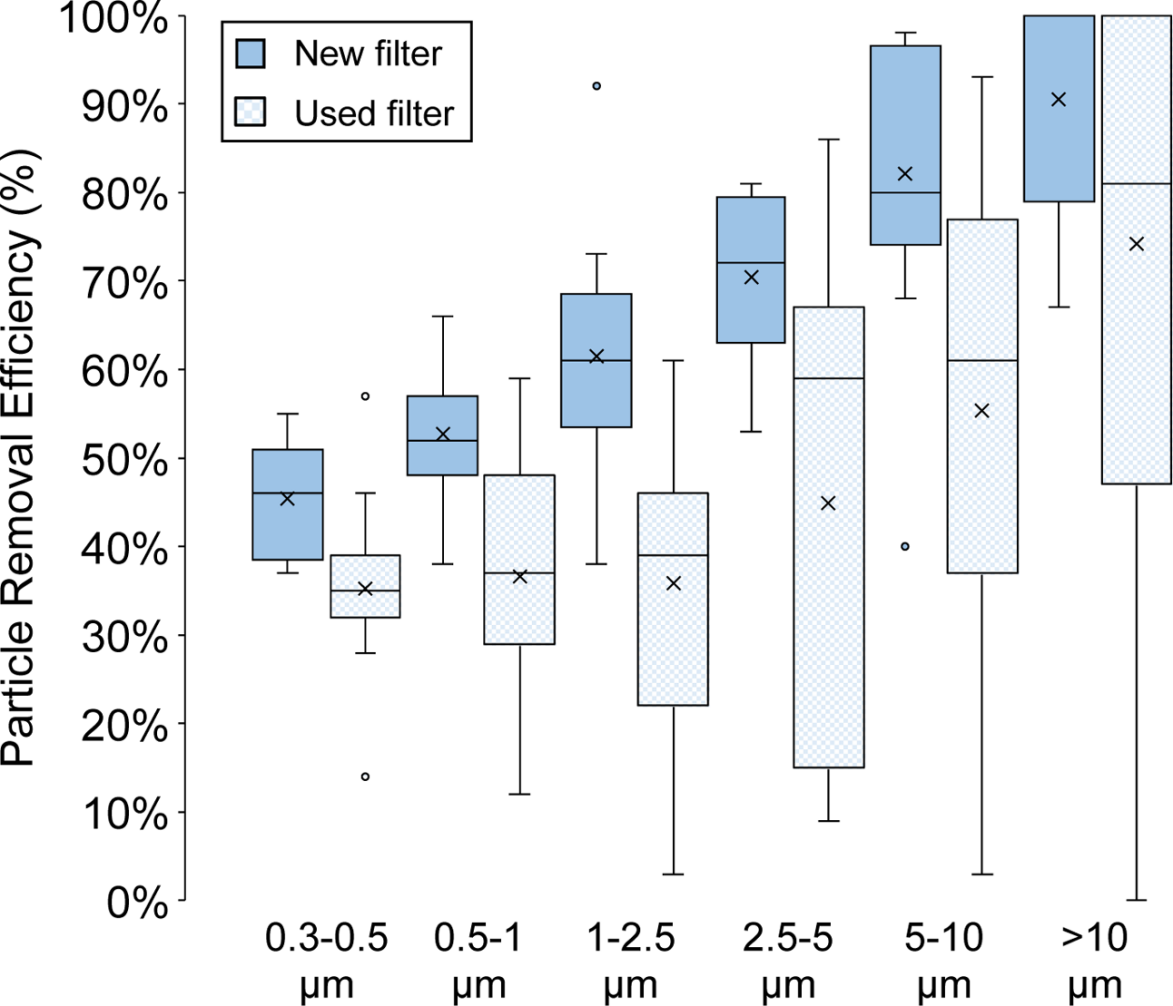
Spot measurements of in situ size-resolved particle removal efficiency of the EC and DIY filter combinations with new filters initially installed and with used filters approximately 3 months after sustained use (*n* = 10).

### Long-Term Measurements

Preintervention and postintervention durations, as well as durations of successful PA data collection, are shown for each home in Figure S1. Based on the timeline of intervention and monitor deployments in each home, the theoretical maximum number of concurrent 10 min interval data points possible within the periods of time when indoor and outdoor PAs were installed ranged from ∼3000 to ∼24,000 in each home (i.e., 6 data points/h, 24 h/day, minimum 21 days of deployment, maximum 165 days of deployment), with a total theoretical maximum number of data points across all 44 homes of just under 700,000. Ultimately, there were 568,980 data points of concurrent indoor and nearest (or next-nearest, or next-next-nearest) outdoor PA measurements at 10 min intervals available for analysis across all 44 homes for which at least some indoor/outdoor PA data were available. Missing data were due to a combination of unstable Wi-Fi connections, power disconnections, or instrument failures. From these available data, the mean (SD) indoor PM_2.5_ concentration, outdoor PM_2.5_ concentration, and I/O PM_2.5_ concentration ratio across all homes and data points (via ALT-CF3) were 8.4 (23.4) μg/m^3^, 6.4 (7.2) μg/m^3^, and 2.0 (9.4), respectively. I/O PM_2.5_ concentration ratios were greater than 1 for ∼52% of the total matched 10 min interval data set across all homes, indicating that substantive indoor sources affected indoor concentrations a slight majority of the time and leaving ∼48% of the full data set for conditional analyses with constrained I/O ratios ≤1.

#### All Available Postintervention Data

Figure 3 utilizes conditional analyses on the two primary PM_2.5_ metrics (constrained I/O ratios ≤1 and *F*_inf_) with all available data points from PAs from all homes in the postintervention periods to quantify the differential impacts of DIY EC filters on indoor PM_2.5_ of predominantly outdoor origin. For both metrics, data from homes with both PAC and DIY EC filters are compared to those with only a PAC, and data from periods in which the ECs were either known or predicted to be operating are compared to periods in which ECs are either known or predicted to be off. Figure 3a shows the distributions of constrained I/O PM_2.5_ concentration ratios across all recorded data points in all homes. Figure 3b shows distributions of PM_2.5_ infiltration factors (*F*_inf_) estimated for the same groups of homes and EC operational conditions. Detailed summaries of the total number of 10 min interval data points included in each category and for each home in Figure 3 are also provided in Tables S3–S5 in the Supporting Information. It should be noted that while some of the box plots in Figure 3 and in subsequent figures appear to result in implausible values of 0 for constrained I/O ratios or *F*_inf_, they are not actually zero but rather slightly above 0. Such low values are rare in the data set but may appear more common in box plots than they really are, especially for plots with wide IQRs, given how whiskers are defined.

**Figure 3:**
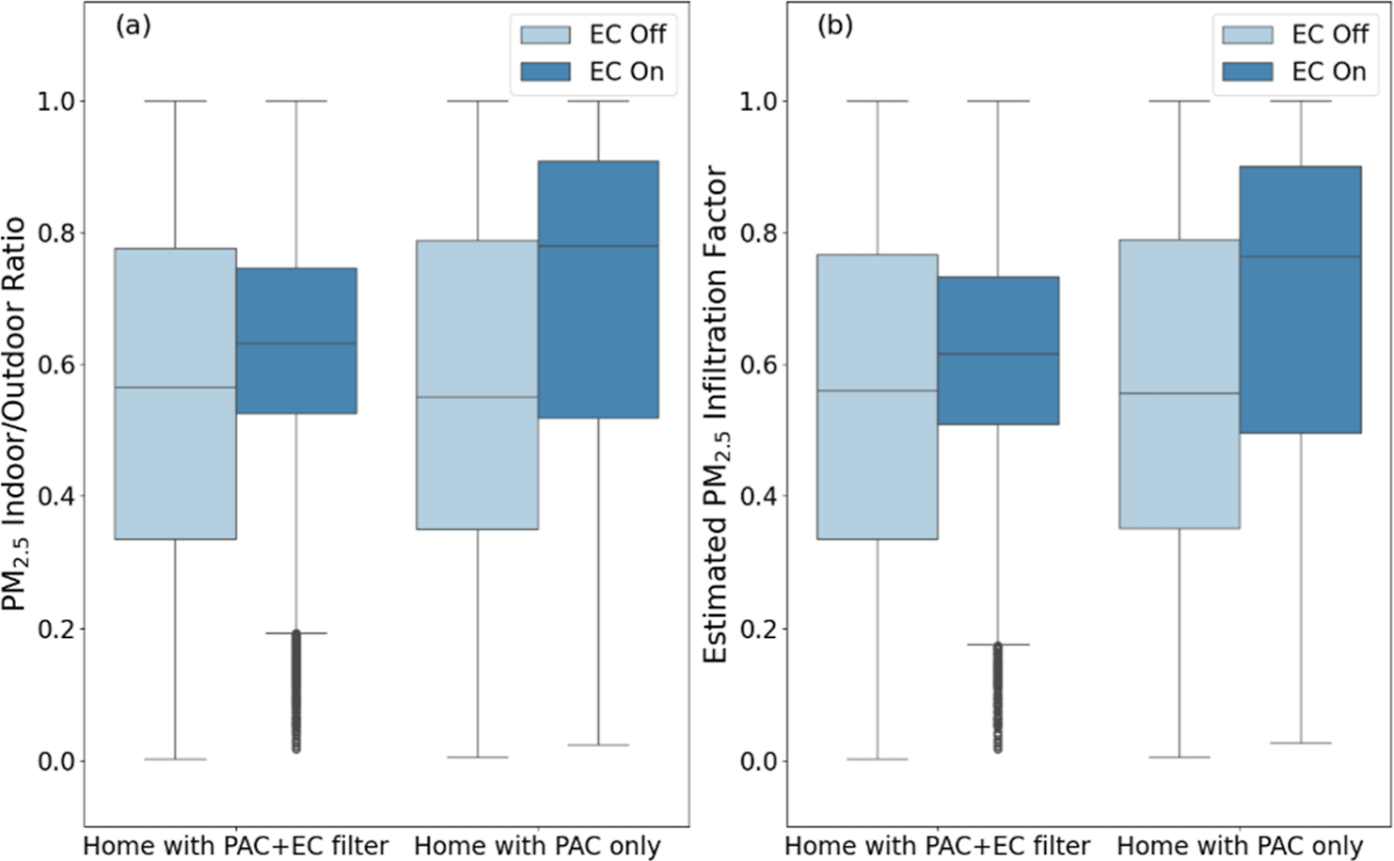
Comparison of (a) PM_2.5_ I/O ratios constrained to I/O ≤ 1 (i.e., excluding indoor sources) and (b) estimated PM_2.5_ infiltration factor (*F*_inf_) between homes with PAC + EC filter homes with PAC only, conditionally comparing when ECs were known or predicted to be on to when they were known or predicted to be off, using all available postintervention data.

Because differences in PAC operation among intervention groups could plausibly affect these comparisons, PAC usage and in situ CADRs were first investigated to determine if further stratification analysis was needed. Most homes operated PACs most frequently on low fan speed settings, accounting for ∼43% of the total duration of measurements. PACs were operated ∼24% and ∼12% of the time on medium and high fan speed settings, respectively, while they were off or unplugged ∼21% of the time. The average daily PAC runtime also gradually declined during the field campaign, from an average of >80% of the time in the first few weeks to only <70% of the time in the last few weeks. The resulting distributions of the in situ CADR delivered in PAC + EC filter homes and PAC-only homes were similar, with a median of ∼27 m^3^/h and ∼28 m^3^/h, respectively, and a small effect size (*d* = 0.23). Given these similarities in PAC operation, the conditional analyses used herein do not account for any differences in PAC operation between intervention groups (see Figures S20–S23 for a summary of PAC usage patterns and in situ CADRs across all homes with available PLL data).

With ECs either known or predicted to be operating, the median constrained I/O ratio in the homes with both PAC and EC filters was 0.63 compared to 0.57 when ECs were either known or predicted to be off. Conversely, the median constrained I/O ratio in the PAC-only homes increased from 0.55 with ECs likely off to 0.78 with ECs likely on. The differences in constrained I/O PM_2.5_ ratios between EC likely on and EC likely off conditions had a larger effect size in PAC-only homes (*d* = 0.53) than in homes with both PAC and EC filters (*d* = 0.36), suggesting DIY EC filtration more successfully mitigated ambient PM_2.5_ infiltration. Similarly, the median PM_2.5_ infiltration factor in the PAC + EC filter homes increased from 0.56 with ECs likely off to 0.62 with ECs likely operating. By comparison, the median PM_2.5_ infiltration factor in the PAC-only homes increased from 0.56 with ECs likely off to 0.76 with ECs likely operating. The differences in PM_2.5_ infiltration factor between EC likely on and off conditions again had a larger effect size in PAC-only homes (*d* = 0.46) compared to that in PAC + EC filter homes (*d* = 0.32). Further, when ECs were known or predicted to be off, constrained I/O ratios and *F*_inf_ were similar across both PAC-only and PAC + EC filter groups, suggesting that the homes were reasonably well randomized in terms of their baseline PM_2.5_ infiltration characteristics with ECs likely off. Also, because these data include periods in which PACs were operating (albeit mostly on low fan speed settings), the magnitudes of constrained I/O PM_2.5_ ratios and *F*_inf_ are likely higher in both intervention groups than they would be without any PAC operation. However, the difference is likely modest, given the relatively low in situ CADRs estimated from PAC runtime measurements. Overall from Figure 4, comparisons of both constrained I/O ratios and *F*_inf_ values similarly demonstrate that in homes without filters installed on the air intakes of their EC, indoor PM_2.5_ of predominantly ambient origin substantially increased when ECs were likely operating (median increase ∼36–42%), whereas the use of DIY EC filters largely mitigated the additional infiltration such that the proportion of ambient PM_2.5_ infiltrating and persisting indoors was only modestly greater with ECs likely on than that observed during EC likely off conditions (median increase ∼10–11%). Comparisons of distributions of home average constrained I/O ratios and *F*_inf_ values yielded similar findings and also allowed for significance testing; differences between EC likely on and off conditions were significant for PAC-only homes but were not significant for PAC + EC filter homes (Figure S8 and Table S12).

**Figure 4:**
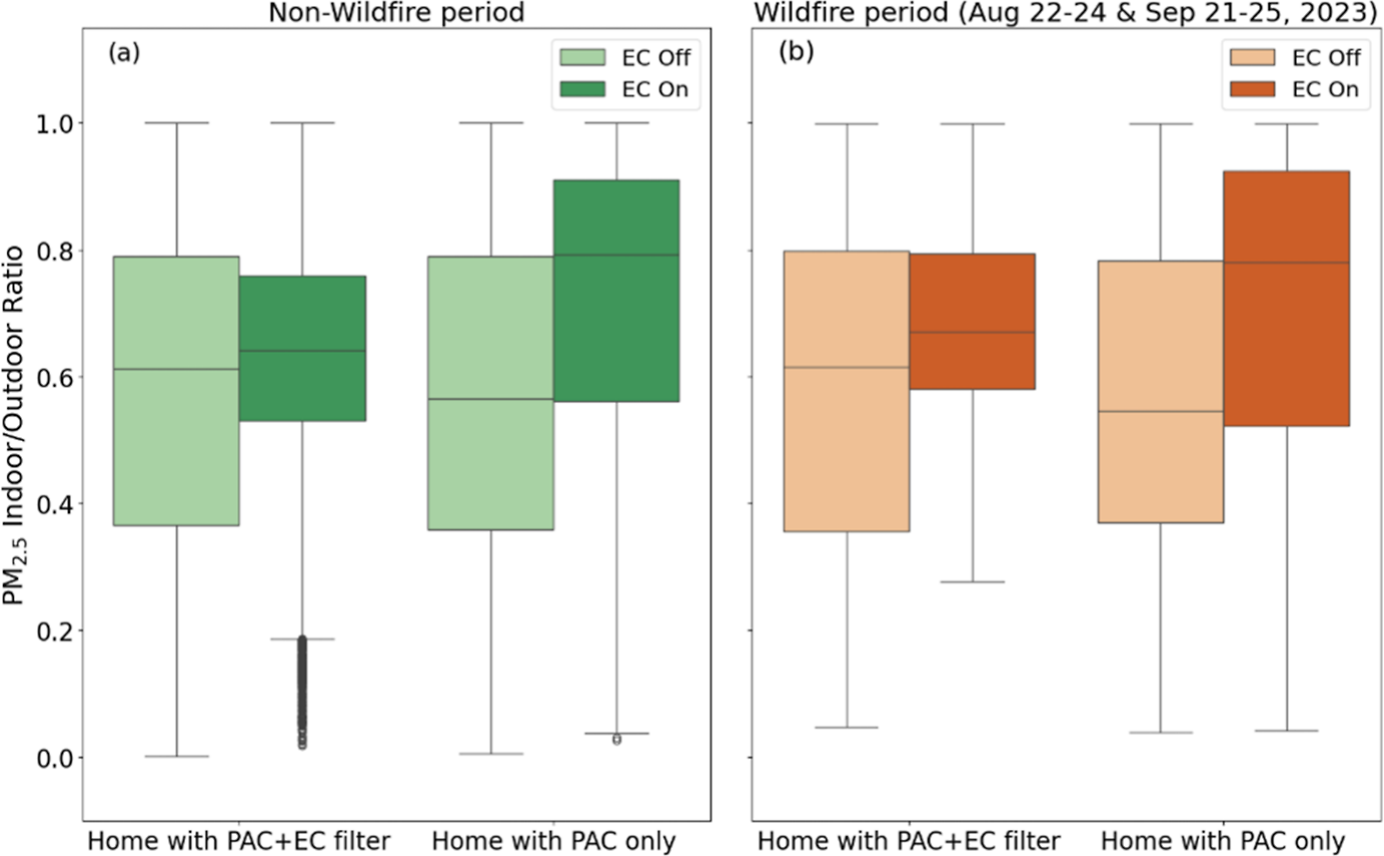
Comparison of constrained I/O PM_2.5_ ratios ≤1 in all homes during (a) nonwildfire periods and (b) wildfire event periods, comparing PAC + EC filter homes vs PAC only homes, and when ECs were measured or predicted on vs off.

As described in the Supporting Information, results were not very sensitive to different assumptions for constrained I/O ratio thresholds or lag periods between indoor and outdoor measurements (Figures S11–S18 in the Supporting Information). Additionally, while both constrained I/O ratios and *F*_inf_ metrics yield similar results and lead to similarly strong correlations between indoor and outdoor PM_2.5_ concentrations, the constrained I/O ratio metric yields a much larger data set to conditionally analyze compared to the *F*_inf_ metric (178,281 vs 89,931 data points at 10 min intervals across all homes). Further, the *F*_inf_ algorithm may incorrectly label some periods as indoor sources when they are not, such as when a rapid increase in outdoor PM_2.5_ concentrations results in a rapid increase in indoor PM_2.5_ concentrations. Therefore, for the remainder of the conditional analyses herein, the constrained I/O ratio ≤1 is used as the primary metric to provide the largest possible data set for comparison, with good confidence that it reasonably reflects ambient PM_2.5_ infiltration. Further, repeating the same analyses in Figure 3 using only true (measured) or only predicted EC runtime data shows similar findings (Figures S6 and S7 and Tables S6–S11), albeit with less data available for analysis. For example, the combination of measured and predicted EC runtime yielded 23,557 *F*_inf_ data points in which the EC was either measured or predicted to be on, but using only measured or predicted runtime limited the analysis to only 18,467 and 12,436 *F*_inf_ values, respectively. Therefore, we primarily rely on the combination of both measured and predicted EC runtimes for the remainder of the analyses to maximize data availability, again with confidence that the combination of measured and predicted EC runtimes reasonably represents likely EC on conditions (EC off prediction is less accurate given the high rate of false negatives).

#### Wildfire vs Nonwildfire Periods

There were two distinct periods of wildfire smoke transport into the region from large fires in Northern California and Oregon during the postintervention period: August 22–24, 2023, and September 21–25, 2023, totaling approximately 8 days. These fires included the Pickett fire in Napa County in California and the Flat fire in Deschutes and Jefferson counties in Oregon in August 2023 and the Anvil and Flat fires in Oregon and the Smith River, Happy Camp, and SRF Lightning Complex fires in California in September 2023. Analyzing data from the two outdoor PAs with the most data available (one at a home in Kern County and one at a home in Fresno County), the median outdoor PM_2.5_ concentration (estimated by PM_2.5__alt) was 21.2 μg/m^3^ during these defined wildfire event periods (including 6.8 μg/m^3^ during the August wildfire event and 23.6 μg/m^3^ during the September wildfire event) compared to a median of 4.2 μg/m^3^ during all other nonwildfire periods (Figure S9 and Table S13). Compared to recent records of ambient PM_2.5_ during wildfire smoke events nationally and in California, these were relatively modest smoke events but also in the range of magnitude of many other wildfires.^75^ For reference, comparisons of hourly concentrations from a single outdoor PA against those measured at the nearest regulatory monitoring site (∼15 km away) suggest that the PM_2.5__alt algorithm yielded estimates of PM_2.5_ concentrations that were within ∼20–30% of the regulatory monitor readings, on average, slightly overestimating during wildfire periods and slightly underestimating during the remaining periods (Figure S10). Figure 4 compares the distribution of constrained I/O PM_2.5_ ratios between the defined wildfire periods and all the other nonwildfire periods in the postintervention data; again, these comparisons do not account for PAC operation, given the similar rates of usage among the two intervention groups.

Differences in the distributions of constrained I/O PM_2.5_ ratios between EC likely on/off conditions and between PAC + EC filter and PAC-only homes were similar during both wildfire and nonwildfire periods. During nonwildfire periods, the median constrained I/O ratio in homes with both PAC and EC filters was 0.61 when ECs were known or predicted to be off compared to 0.64 (+5%) when ECs were known or predicted to be on, while the median constrained I/O PM_2.5_ ratio in homes with only PACs was 0.57 when ECs were known or predicted to be off compared to 0.79 when likely on (+39%) (both were similar to Figure 3, given the large amount of nonwildfire periods, with comparable effect sizes of *d* = 0.58 and *d* = 0.29, respectively). During the wildfire periods, the median constrained I/O PM_2.5_ ratio in the homes with both PAC and EC filters increased from 0.62 with ECs known or predicted to be off to 0.67 with ECs known or predicted to be on (+8%; *d* = 0.52), while the same values increased from 0.55 to 0.78 (+42%; *d* = 0.64) in PAC-only homes. These comparisons suggest that the constrained I/O ratio during nonwildfire periods, which account for most of the data collection period due to infrequent wildfire smoke events during the monitoring campaign, provides a strong indicator of the infiltration of ambient PM_2.5_ indoors that can also be used to infer performance during wildfire event periods. Additional details are provided in Tables S14–S16.

#### New vs Used Filters

To investigate whether the effectiveness of the deployed DIY EC filters diminished over time, Figure 5 shows constrained I/O PM_2.5_ ratios compared using conditional analyses in the homes with both PAC and EC filters during two different 3 week periods: (i) the first 3 week period immediately following EC filter installation and (ii) 3 weeks beginning 30 days after the end of the initial 3 week period following installation. Sixteen homes with PAC and EC filters had data available during the first 3 weeks, and 12 homes had data during the second 3 weeks (some homes completed the study prior to reaching >50 days or lost data from PAs). Only homes with at least 144 data points (equivalent to 24 h of data at 10 min intervals, albeit not necessarily consecutive) were included in this analysis.

**Figure 5:**
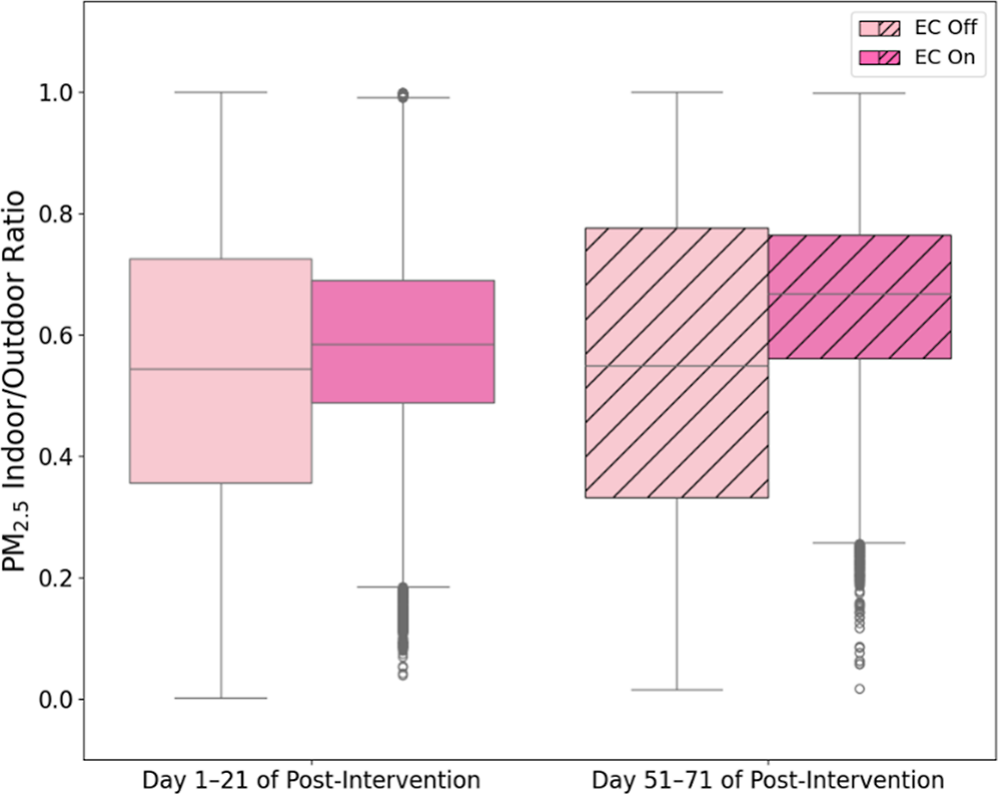
Comparison of constrained I/O PM_2.5_ ratios ≤1 in homes with PACs and EC filters, comparing data when ECs were measured or predicted on vs off and comparing between newly installed EC filters (day 1–21 following installation) and used EC filters (day 51–71 following installation).

In the first 21 days following EC filter installation, the median constrained I/O ratio increased from 0.55 when ECs were known or predicted to be off to 0.59 when ECs were known or predicted to be operating (+7%; *d* = 0.22). In the 21 days following the initial 50 days of installation, the median constrained I/O PM_2.5_ ratio increased from 0.55 when ECs were likely off to 0.69 when ECs were likely operating (+25%; *d* = 0.46). Details are summarized in Tables S17 and S18. These comparisons demonstrate that the effectiveness of the EC filters in mitigating the infiltration of ambient PM_2.5_ appeared to decline over time during the 2–3 months of deployment, although the median increase in the constrained I/O ratio at 51–71 days was still smaller than the increase in homes with only PAC. This finding supports the recommendation to utilize DIY EC filters temporarily, for example, deploying only when ambient PM_2.5_ is elevated due to wildfire smoke conditions and removing them when the episode subsides (i.e., installing for up to several days at a time).

#### Pre/Postintervention by Home

In a subset of homes with data available, constrained I/O PM_2.5_ ratios were compared within homes before and after interventions when ECs were either known or predicted to be operating, and those individual home differences were compared across intervention groups (Figure 6). Only homes that had at least 3 weeks of data available during both pre- and postintervention periods were included in this analysis, which resulted in data from 10 PAC-only homes and 11 homes with both PAC and EC filters. Among the 10 PAC-only homes, 6 homes had detectably lower median constrained I/O PM_2.5_ ratios after the intervention, and the constrained I/O ratio decreased by more than 10% in four of those homes. Conversely, among the 11 PAC + EC filter homes, 10 homes had detectably lower constrained I/O PM_2.5_ ratios after the intervention, and 7 decreased by more than 10%. Aggregating across all pre/postintervention data from all homes within the two intervention groups (Figure S19), the median constrained I/O PM_2.5_ ratio decreased from 0.82 preintervention to 0.81 postintervention (−1%; *d* = 0.31) in PAC-only homes but decreased from 0.86 preintervention to 0.66 postintervention (−23%; *d* = 0.74) in homes with both PAC + EC filters. Additional details are provided in Tables S19 and S20.

**Figure 6:**
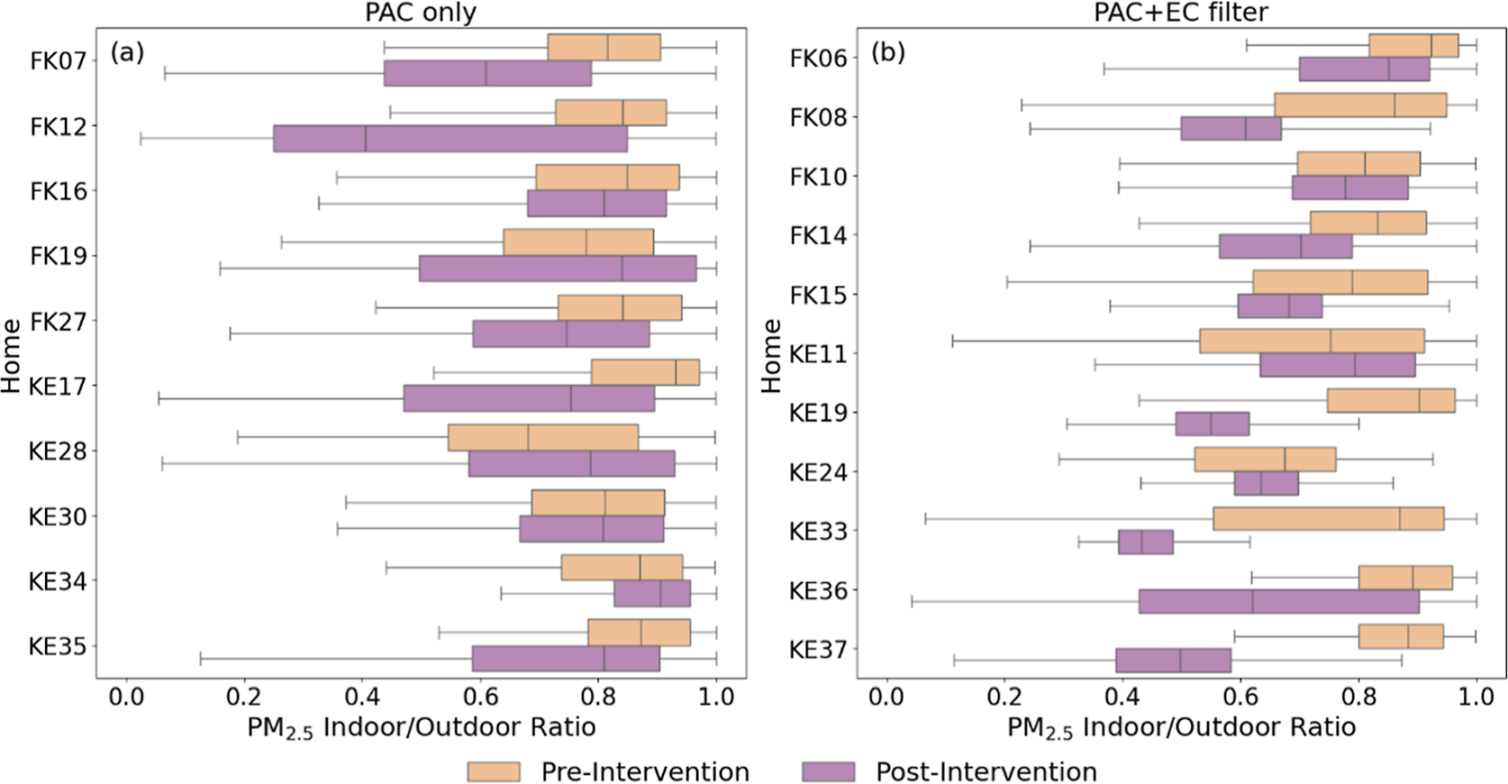
Comparison of constrained I/O PM_2.5_ ratios between preintervention and postintervention periods within individual homes when ECs were either known or predicted to be operating: (a) PAC-only homes and (b) PAC + EC filter homes.

#### Comparing PACs and EC Filters

Even with modest operation overall, the usage of PACs did appear to reduce indoor PM_2.5_ of ambient origin across all homes, as constrained I/O PM_2.5_ ratios were lower in both PAC-only and PAC + EC filter homes with the PACs operating compared to those with the PACs off (Figure S24 and Table S21). While this demonstrates the beneficial effect that PACs can have on indoor concentrations of ambient PM_2.5_, the effect only occurs after PM_2.5_ first infiltrates indoors. Thus, it is worth comparing the two approaches to air filtration in other ways. First, we compare results from the conditional analyses of field-collected data to theoretical values of PM_2.5_ *F*_inf_ that would be expected when ECs are operating using a steady-state well-mixed mass balance approach, with inputs informed by the field study (eq 1)­

1 $$F_{\inf}=\frac{C_{in}}{C_{out}}=\frac{P\lambda }{\lambda +k+\left(\frac{CADR}{V}\right)}$$

where *P* is the PM_2.5_ penetration factor through the EC with or without DIY filters (dimensionless), λ is the air change rate in the home while the EC is operating (1/h), *k* is the particle deposition loss rate coefficient inside the home (1/h), *CADR* is the average CADR for PM_2.5_ of a PAC operating inside the home (m^3^/h), and *V* is the home volume (m^3^). Assuming an average home volume in our study of 300 m^3^ and average EC supply airflow rates of 2300 m^3^/h and 2700 m^3^/h with and without DIY EC filters, respectively, the resulting air change rate due to EC supply airflow would be 7.4 1/h and 8.7 1/h with and without EC filters. Since these values are much greater than typical air infiltration rates in homes,^76,77^ we assume these values reasonably approximate total λ while ECs are operating and that other sources of air infiltration are negligible. Assuming the 0.3–0.5 μm size bin most closely represents EC removal efficiency for PM_2.5_, the PM_2.5_ efficiency of ECs with and without DIY filters may be approximated as 40% and 5%, respectively, making *P* = 0.6 with filters and *P* = 0.95 without filters.^42^ Finally, we assume *k* = 0.5 1/h as a reasonable value within typical ranges for PM_2.5_ based on prior literature^78–81^ and that the average *CADR* = 60 m^3^/h, both of which are assumed to be the same in homes with and without DIY filters. With these assumptions, we expect *F*_inf_ for PM_2.5_ to be ∼0.88 in a home without EC filters and ∼0.55 in a home with DIY MERV 13 EC filters. These estimates are within 13–16% and 12–13% of our median constrained I/O and *F*_inf_ values from the field study (Figure 3), suggesting our conditional analysis approaches using field-collected data are within reasonable bounds of expected values.

Second, given the very large airflow rates through ECs when operating, the air change rate delivered by ECs is reasonably expected to be the dominant PM_2.5_ removal mechanism present inside the home. Therefore, we also approximate how much additional indoor air cleaning by PACs in a home without EC filters would be needed to achieve the same level of reduction in *F*_inf_ as achievable by the EC filters by setting *F*_inf_ = 0.55 and solving for CADR in eq 1. Doing so yields an additional 1800 m^3^/h of CADR from the PACs that would be needed to provide the same level of *F*_inf_ reduction as that of the EC filters (i.e., from 0.88 to 0.55). For context, the manufacturer-rated CADR for smoke-sized particles for the largest PAC used in this study was ∼480 m^3^/h operating at high fan speed. Assuming that 480 m^3^/h also reasonably approximates the CADR for PM_2.5_, the average home would need approximately 4 of the largest PACs operating on high fan speed settings to achieve the same level of reduction in *F*_inf_ as the DIY EC filters would. In a home with the smaller PACs used herein (CADR = 225 m^3^/h), approximately 8 PACs would need to be running on high to achieve the same level of *F*_inf_ reduction. At typical retail costs of ∼$100 for the smallest PAC and ∼$200 for the largest PAC in this study, an additional ∼$800 in upfront costs for PACs would be needed to achieve the same level of *F*_inf_ reduction as would be expected from the DIY EC filters, while the total upfront cost of filters needed to cover the EC intakes was $60–$180 depending on EC size. The use of DIY PAC solutions such as Corsi–Rosenthal (CR) boxes that combine a box fan with MERV 13 filters could also achieve a similar level of *F*_inf_ reduction as DIY EC filters at lower cost than many commercial PACs, necessitating perhaps one CR box operating on high (CADR ∼ 1400 m^3^/h for 0.3 μm particles) or two CR boxes operating on low (CADR ∼ 1000 m^3^/h each).^82^ Differences in operational electricity costs between EC filters and commercially available or DIY PACs to achieve the same level of *F*_inf_ reduction as EC filtration are likely negligible since ECs commonly draw ∼300–400 W when operating, and 4–8 commercial PACs or 1–2 CR boxes operating on high fan speed settings may draw ∼200–350 W depending on make, model, and quantity. Operating the most efficient PACs in our study would yield differences of no more than ∼$2/day in electricity costs for continuous operation at an average residential electricity rate of ∼$0.30/kWh in California. Importantly, introducing 4–8 PACs or 1–2 CR boxes into these homes with an average floor area of ∼130 m^2^ is also practically challenging, given space constraints. These approximations also suggest that the use of lower MERV (e.g., MERV 8 or MERV 11) on EC intakes could also likely achieve greater reductions in *F*_inf_ than practically feasible with PACs operating indoors at a lower cost than the MERV 13 filters tested herein.

#### Limitations and Conclusions

There are several limitations to this work. For one, many of our conditional analyses and predictions of EC runtime were necessitated by the realities of the pragmatic study design and execution, which involved coordination across teams in different locations and with different skill sets and training, in addition to coordination between community organizations and study participants with varied availability for home access. Two, although the EC filtration solutions were developed specifically to address wildfire smoke infiltration, there were not many days with elevated PM_2.5_ concentrations due to wildfire smoke in the study communities during our measurements, which led to many of our analyses focusing on relatively low ambient and indoor PM_2.5_ concentrations. Sometimes these values were near or below the functional limit of detection of the PAs (i.e., 1 μg/m^3^). While this may have affected the accuracy of I/O ratios and *F*_inf_ calculations, our colocation measurements also suggest the monitors perform well below this functional limit.^83^ Additionally, while EC filters demonstrated benefits, EC filters obviously provide benefits only when the EC is operating, which ranged from an average of <20% of the time during mild weather to >50% of the time during warmer weather (Figure S25). There may be other solutions for these homes that could provide longer-lasting benefits, such as switching from ECs to high-efficiency minisplit systems and air-sealing envelopes to mitigate PM infiltration, although these would be expensive and likely infeasible options for many project participants. Another practical consideration is the impact of added EC filtration solutions, leading to reliability concerns, such as overheating or fan failure due to an excessive pressure drop. At least for the solutions and durations tested herein, no evidence of such failures was observed, perhaps in part because each unit received maintenance prior to filter installation. This does not rule out the possibility of problems on other EC units that receive higher-pressure-drop filters, especially if the units are not serviced in advance. Finally, the present work describes performance measures based only on measurements of PM_2.5_ with low-cost monitors; other components of the larger FRESSCA-Mujeres project that measured metals and gas-phase VOCs and SVOCs in air and urine samples are presented elsewhere.^72^ This work also did not evaluate the impacts of EC filtration or PACs on other pollutants of concern in the region, such as pesticide-derived air pollution, ambient ozone, or fungal spores that could lead to fungal growth on moist EC pads, although the developed solutions may also help address some of these issues as well.

Despite these limitations, these findings suggest that DIY EC filtration solutions offer a practical and affordable strategy to reduce indoor exposure to outdoor PM_2.5_ in homes with ECs. In our primary conditional analyses with the full data set, the median level of ambient PM_2.5_ infiltration (evaluated using two different metrics) increased by only ∼10–11% during EC likely on vs likely off conditions in homes with DIY EC filters and a PAC but increased by ∼36–42% in homes with only PACs. Analyses were robust with respect to wildfire vs nonwildfire periods and to assumptions for lag times between indoor and outdoor concentrations and constrained I/O ratio thresholds. Comparisons using home mean values of ambient PM_2.5_ infiltration metrics and pre/postintervention comparisons in a subset of homes with data available both confirmed the magnitude and direction of PM_2.5_ infiltration reductions with EC filters observed in the larger data set. Filter performance declined after extended use, likely for a combination of reasons. As such, these findings suggest that the temporary use of DIY EC filtration solutions during wildfire smoke events can help cost-effectively improve indoor air quality. We also demonstrate that even modest filtration at the source through which air is delivered into homes with ECs operating (i.e., EC air intakes) is likely to achieve a greater reduction in ambient PM_2.5_ infiltration than most feasible arrangements of indoor HEPA-based PACs, such that even lower levels of EC filtration (e.g., MERV 8 or MERV 11) can likely provide some reductions in PM_2.5_ infiltration through ECs at even lower cost than MERV 13 filters. Finally, the combination of DIY EC filtration and indoor PACs, whether commercially available units or DIY solutions such as CR boxes, offers promising opportunities to simultaneously reduce indoor PM_2.5_ of both ambient and indoor origin.

## Supplementary Material



## Notes

Additional/expanded materials, methods, results, and discussion, including pilot year testing, data collection timeline and availability summary from intervention year, calculations of PM_2.5_ infiltration factors and EC runtimes, tests of assumptions for indoor/outdoor PM_2.5_ lag times and constrained I/O ratio thresholds, and expanded results for group comparisons; PAC and EC runtimes and impacts; and individual home characteristics and spot measurements (PDF)
