# Supplementary material for: Field Evaluation of Do-It-Yourself Air Filtration Solutions for Evaporative Coolers to Reduce Ambient Particle Infiltration in Homes in Wildfire-Affected Communities
Source: ACS EST Air. 2026 Mar 10;3(4):1144–56. doi: 10.1021/acsestair.5c00488 (PMC13077632; doi:10.1021/acsestair.5c00488)
Supplement: Supplementary file 1 [file ea5c00488_si_001.pdf]

Supporting Information (SI) for:

Field evaluation of do-it-yourself (DIY) air filtration solutions for evaporative coolers to reduce ambient particle infiltration in homes in wildfire-affected communities

Mingyu Wang [a], Aditya Singh [a], David Chang [b], Isabella Kaser [b], Jeff Wagner [c], Zhong-Min Wang [c], Brett C. Singer [d], Shelly L. Miller [e], Nayamin Martinez [f], Ruben Rodriguez [f], Stephanie Jarmul [g], McKenna Thompson [g], Peggy Reynolds [h], Julie Von Behren [h], John R. Balmes [i,j], Mohammad Heidarinejad [a], Brent Stephens [a]*, Gina Solomon [i]

[a] Department of Civil, Architectural, and Environmental Engineering, Illinois Institute of Technology, Chicago, IL 60616 USA
[b] Tracking California, Public Health Institute, Oakland, CA 94607 USA
[c] California Department of Public Health, Center for Laboratory Sciences, Environmental Health Laboratory, Richmond, CA 94804 USA
[d] Lawrence Berkley National Laboratory, Berkeley, CA 94720 USA
[e] Department of Mechanical Engineering, University of Colorado, Boulder, CO 80309 USA
[f] Central California Environmental Justice Network, Fresno, CA 93727 USA
[g] Office of Environmental Health Hazard Assessment, California Environmental Protection Agency, Oakland, CA 94612 USA
[h] Department of Epidemiology and Biostatistics, University of California, San Francisco, CA 94158 USA
[i] School of Medicine, University of California, San Francisco, CA 94143 USA
[j] Division of Environmental Health Sciences, School of Public Health, University of California, Berkeley, CA 94704 USA

*Corresponding author:
Brent Stephens, PhD
Professor and Department Chair
Arthur W. Hill Endowed Chair in Sustainability
Department of Civil, Architectural, and Environmental Engineering
Illinois Institute of Technology
Alumni Memorial Hall Room 228
3201 S Dearborn Street
Chicago, IL 60616 USA
bstephe5@illinoistech.edu

Supplemental Materials and Methods

Pilot testing

In the initial pilot year of field testing (2022), participants in 31 homes were recruited in three communities within Fresno and Kern Counties (Coalinga, Arvin, and Lamont). Participants in 25 homes successfully completed pilot testing. During this phase, we tested the use of participant questionnaires, installed PurpleAir PA-II or PA-II-SD monitors inside each home, and installed 5 PA-II monitors in several nearby outdoor locations (2 in Kern County and 3 in Fresno County). In July 2022, 9 homes received only a DIY EC filtration solution (i.e., a 10-cm deep MERV 13 filter impregnated with activated carbon attached to their EC intake), 9 homes received only a PAC with HEPA as a commonly recommended wildfire smoke mitigation measure, 6 homes received only a DIY box fan and MERV 13 filter combination as an emerging low-cost wildfire smoke mitigation measure, and 1 home received no intervention. However, a total of 4 homes practically served as control homes by reporting that they rarely used their provided intervention (i.e., 2 of the homes with DIY box fan and filter) or by intervention failure (i.e., the plastic wrap around the HEPA filter in one home with a PAC was never removed, so the intervention did not work as intended). Monitoring continued in each home until October 2022 when the equipment was retrieved. At that time, data from only 21 of 25 homes was successfully retrieved due to data losses from the Wi-Fi connected PA-II monitors.

Preliminary data and lessons learned in the pilot year were used to inform full-scale intervention testing in the following year. For example, many of the indoor PA-II monitors were Wi-Fi-only monitors without onboard storage, and we found that frequent Wi-Fi connection issues resulted in extensive periods of lost data that limited the amount of data available for analysis. This led to the selection of a greater number of PA-II-SD monitors with onboard SD card storage in the second year of testing to improve data collection success rates. PurpleAir also informed us early during the pilot phase that the PA-II monitors needed to be replaced due to faulty sensor algorithms, which limited our ability to use the pre-intervention PA data in the pilot year (faulty monitors were replaced during the July 2022 pilot intervention deployments). Additionally, we received feedback from participants and other stakeholders during the pilot year that it would be ideal for this community-based intervention project, rather than having control homes without any intervention, to provide all participants with at least one mitigation solution to improve indoor air quality and reduce smoke infiltration and persistence in the event of wildfire. We also heard from study participants that they greatly preferred the PAC to the box fan and filter intervention due to aesthetics and noise issues. Most of the remaining methods and results herein focus on the full-scale intervention in year 2.

Intervention year field campaign: data availability by intervention group

Table S1 summarizes home data collection and data availability by intervention group from the 2023 field campaign. At least some amount of data was successfully collected from 44 homes, including 21 homes with PAC only and 23 homes with both a PAC and DIY EC filtration solution. A total of 39 homes had at least some pre-intervention data available from PurpleAir (PA) monitors, while 36 homes had at least some post-intervention PA data available. Only 14 homes had plug load logger (PLL) data from their evaporative coolers (ECs) available during the pre-intervention period (about half of the DIY filtration intervention homes did not receive their EC PLLs until the filtration intervention was installed), while 25 homes had EC PLL data from the post-intervention period (yet skewed highly towards homes with PAC and EC filter interventions given that most PAC only homes had inaccessible rooftop ECs). A total of 34 homes ultimately had PLL data available from their PAC interventions, split evenly between intervention groups (PAC PLL data collection failed in 10 homes for various reasons, including failure to launch correctly, instrument malfunction due to disturbances such as spills or pests, or plugged in incorrectly). Separating by intervention type, for homes with PAC only, 18 homes yielded PA data and 3 homes yielded EC PLL data during the pre-intervention period, while 18 homes yielded PA data, 5 yielded EC PLL data, and 17 yielded PAC PLL data during the post-intervention period. For homes with both PAC and EC filters, 21 homes yielded PA data and 11 homes yielded EC PLL data during the pre-intervention period, while 18 homes yielded PA data, 21 homes yielded EC PLL data, and 17 homes yielded PAC PLL data during the post-intervention period.

Table S1. Summary of home data collection and availability by intervention group

Summary of home data collection/availability	# of homes with PAC only	# of homes with PAC and EC filter	Total
Total homes	21	23	44
Homes with pre-intervention PA data	18	21	39
Homes with post-intervention PA data	18	18	36
Homes with pre-intervention PLL data on EC	3	11	14
Homes with post-intervention PLL data on EC	5	20	25
Homes with post-intervention PLL data on PAC	17	17	34

Figure S1 shows the intervention deployment timeline and measurement duration for pre- and post-intervention periods, as well as the amount of PurpleAir data successfully collected in each home. The full-scale intervention year field campaign ran from April 2023 to October 2023, with monitor deployments gradually conducted in homes based on the field team's sequential home recruitment and participant scheduling. Most of the homes received pre-intervention

monitoring sometime between April and July 2023, with interventions occurring mostly in late July 2023, although several homes received only post-intervention monitoring and/or were recruited later in the timeline based on participant response and availability. The PurpleAir data availability also varied by home. Several homes (e.g., FK08, KE30) had PurpleAir data coverage throughout most of their monitoring periods, with minimal data gaps. However, many homes (e.g., FK03, KE38) had intermittent data missing, with notable gaps in PurpleAir data availability due to unstable Wi-Fi connections, power disconnections, or other technical issues.

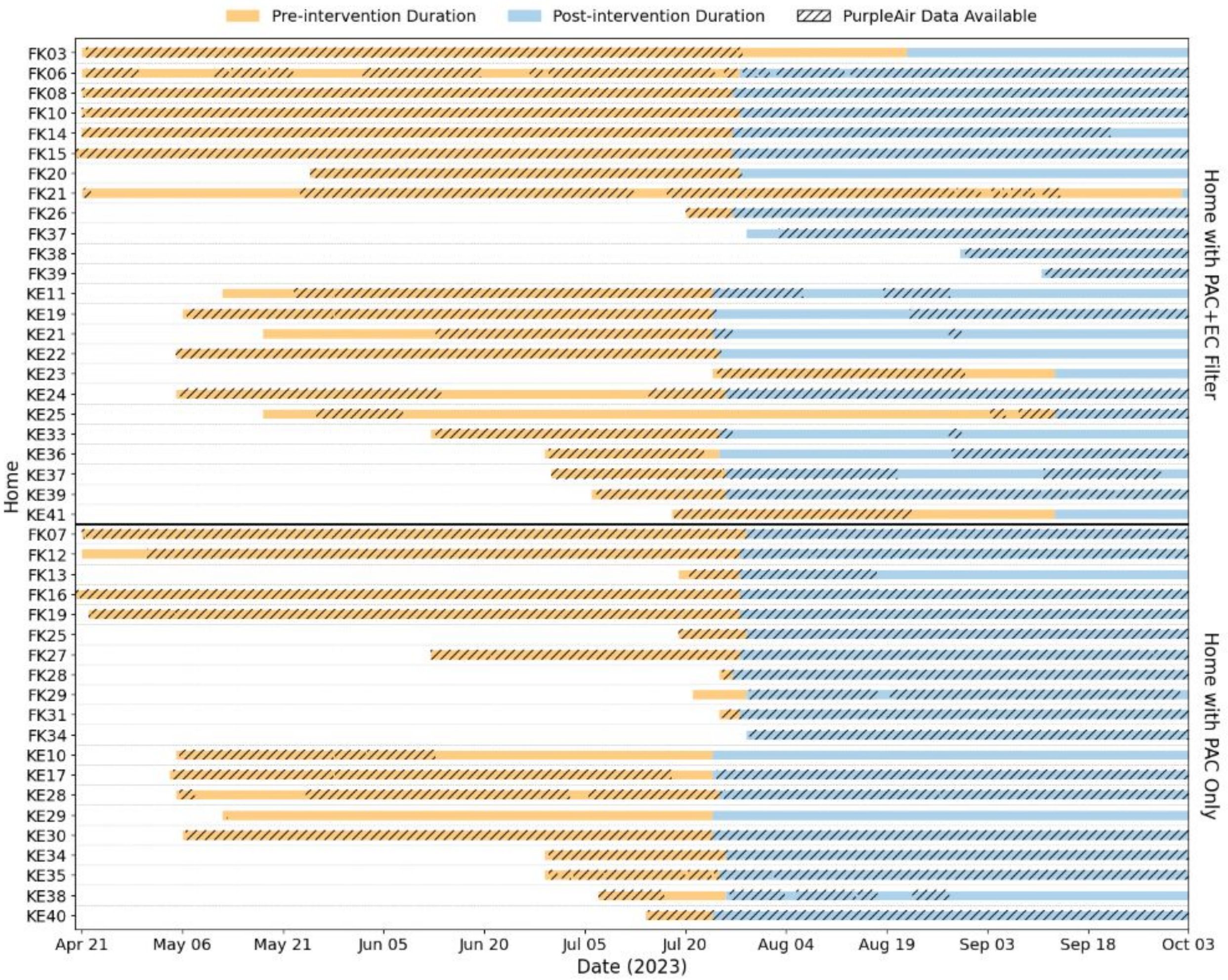

Figure S1. Intervention timeline, measurement duration, and PurpleAir data availability for each home in the full-scale intervention year (2023)

Calculating $PM_{2.5}$ infiltration factors (F_{inf})

To estimate the ambient $PM_{2.5}$ infiltration factor from time-resolved indoor and outdoor PA data, periods influenced by indoor PM sources are identified and excluded.[1–5] The methodology for identifying indoor sources for eventual exclusion leverages the concept of statistical dispersion, characterized by a z-score. If a subsequent indoor $PM_{2.5}$ concentration data point in a time-series deviates from a moving average by more than a specified multiplier of the moving standard deviation (i.e., a 'threshold'), the point is considered a potential anomaly. We chose a multiplier of 5 based on iterative visual inspection. We used a moving average window of 12 continuous data points (i.e., 120 minutes), calculating the mean and standard deviation over this window at each time step. The next data point following the lagging window was classified as a potential indoor source (signal = 1) if its absolute deviation from the mean of the previous 12 data points was more than 5 times the standard deviation of those previous 12 data points. All other values were considered normal (signal = 0). To prevent peak values from affecting the moving average, if a point was flagged as a potential anomaly (signal = 1), its concurrent value used for calculating the moving average was computed as a weighted combination of the current value and the previous value using an influence factor (Equation S1).

$$\overline{y_i} = influence\,factor \times y_i + (1 - influence\,factor) \times y_{i-1} \qquad \text{(S1)}$$

where $\overline{y_i}$ is the concurrent value of anomaly used for calculating the average at time *i*, the influence factor is a factor that determines the influence of the anomaly value to the moving average window (0.05 was used herein, determined via iteration), y_i is the raw observation value at time *i*, and y_{i-1} is the raw observation value at time *i*-1. This method ensured that peaks do not disproportionately shift the moving average baseline, thus enhancing the robustness of the detection against transient fluctuations.

Two additional steps were taken to flag data points as either signal = 1 or signal = 0 in case the method above failed to detect some indoor source periods: (a) identifying data points where indoor $PM_{2.5}$ concentration is higher than the concurrent outdoor $PM_{2.5}$ concentration (I/O > 1); and (b) excluding data points where the increase in indoor $PM_{2.5}$ concentration between two consecutive points is higher than the increase in the concurrent outdoor $PM_{2.5}$. An example application of these methods to identify indoor $PM_{2.5}$ of only ambient origin (shown as green dots) is provided in **Figure S2**. F_{inf} was then calculated at each time step as the indoor $PM_{2.5}$ concentration without sources (green dots) divided by the concurrently measured outdoor $PM_{2.5}$ concentration (i.e., I/O ratio in the absence of indoor sources).

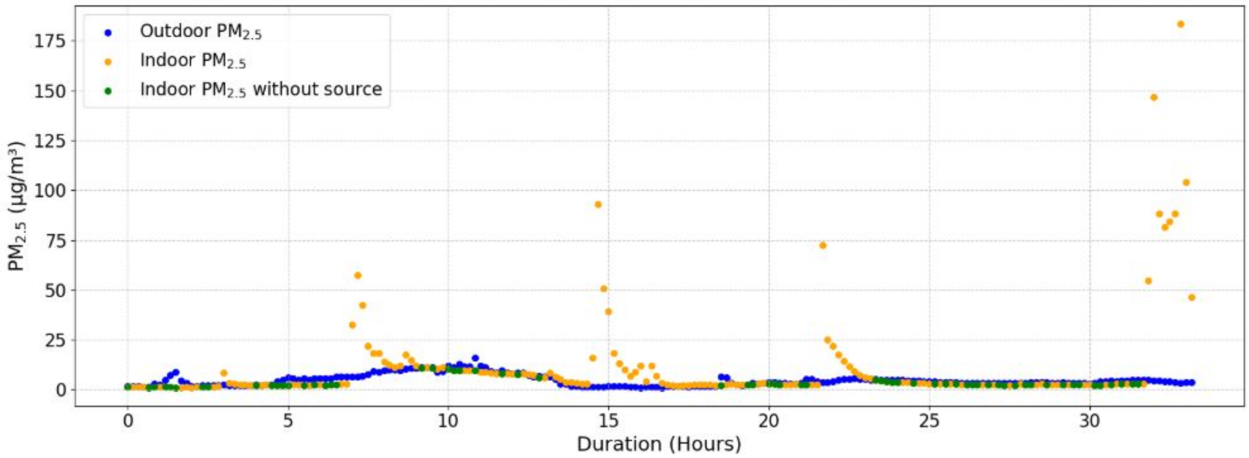

Figure S2. Example of data processing to identify indoor $PM_{2.5}$ of only ambient origin (i.e., 'without source') in a home

This approach to estimating F_{inf} has some limitations. For example, if outdoor concentrations increase rapidly, leading to relatively rapid increases in indoor concentrations, those indoor elevations can be incorrectly flagged as an indoor-generated source rather than outdoor-infiltrated. However, such behavior is not consistently detectable, and such dynamics in the data set lack of a generalizable pattern. Differences in outdoor-to-indoor signals in cases like this may be attributable to a combination of factors such as whether the EC was on or off (or likely on or off in our predicted runtime data set), whether windows could have been open or not (unknown in our data set), whether or not an EC filter was installed, varying distances to the nearest outdoor monitors (often close but sometimes farther), and whether or not indoor sources appeared during this same time period of rapid and then sustained increases in outdoor concentrations (which we noticed happening not infrequently in our review of time-series data such that one can no longer distinguish outdoor sources from indoor sources). As such, the F_{inf} algorithm does not account for these scenarios.

Predicting EC runtime

We investigated the potential use of indoor temperature, indoor relative humidity (RH), and indoor humidity ratio, as well as the difference between concurrent indoor and outdoor values for these parameters, measured by the indoor PurpleAir monitors for predicting EC run mode in homes such that we could potentially predict EC runtime in homes without PLLs on their EC. Using true run mode data from homes with PLLs installed on their EC, we visually observed in the time-series data that often when the EC turned on, the indoor RH rapidly increased, which is reasonable given how ECs function (i.e., providing cooler air but with higher RH at constant enthalpy to the space). An example of time-series indoor RH data along with real PLL-measured

EC runtime is shown in **Figure S3**. In this example, there were 5 periods during which the EC was measured to be operating (via PLL) and indoor RH also rapidly increased at the same time that the EC turned on. This pattern was consistently observed by visual inspection across most homes. Therefore, we developed a custom Python script to automatically identify the rising portion of each indoor RH peak as the most promising indicator of runtime and mark those time stamps as ‘EC likely on’ (i.e., 1). All other times were marked as ‘EC likely off’ (i.e., 0).

The methodology to identify the rising indoor RH portions of time-series data has the same logic as the method to identify periods with indoor $PM_{2.5}$ sources, but a potential rising portion of indoor RH (signal = 1) was identified only if its deviation from the mean of the previous lagging window was higher than the threshold times the standard deviation of the previous lagging window. We used a lagging window of 5 and an influence factor of 0.5 to identify the rising indoor RH portions, established by iteration with visual inspection. We also tested the rising indoor RH method with different thresholds (i.e., from 1 to 3.5 in increments of 0.5) and length of the rising portion of the peak (i.e., from no limit, to lasting more than 10 minutes, to lasting more than 20 minutes). We generated a confusion matrix to inform eventual parameter selection for this method, comparing known (truth) EC runtime values (measured by PLL) with the estimated EC runtime to quantify the performance of this prediction method with various tuning parameters.

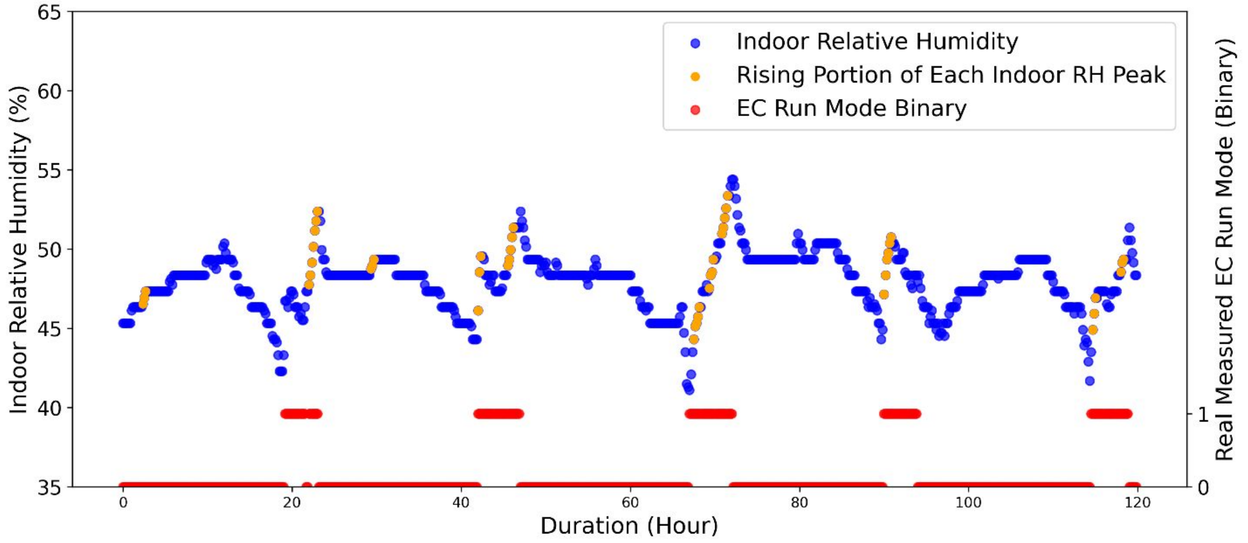

Figure S3. Example of runtime prediction algorithm results using time series indoor RH data (via PA) compared to real measured EC runtime (via PLL)

We compared the utility of these approaches using four different factors to understand model performance in context with how much data are included/excluded using data from in homes with known EC run modes (i.e., in homes with PLLs installed on their ECs), including:

- Total number of data points predicted as EC 'on'. Higher number of data points was desirable to maximize data available for subsequent conditional analysis.
- False positive rate (FPR), calculated as the number of data points in which the EC is predicted to be 'on' but the EC is actually 'off', divided by the total number of data points in which the EC is actually 'off'. Lower FPR is desirable, as we do not wish to analyze data thinking the EC is on but it is actually off. It is important to note that a high true positive rate, or TPR, calculated as the number of data points in which the EC is predicted to be 'on' and is actually 'on', was difficult to attain and thus TPR was not a primary criterion for method selection.
- Accuracy, calculated as the sum of accurately predicted time points (i.e., number of data points when the EC is predicted 'on' and is actually 'on' plus the number of data points when the EC is predicted 'off' and is actually 'off'), divided by the total number of data points available. Higher accuracy is desirable.
- Diagnostic odds ratio (DOR), calculated as the positive likelihood ratio (i.e., TPR divided by FPR) divided by the negative likelihood ratio (i.e., the false negative rate, or FNR, divided by the true negative rate, or TNR) (high DOR, ideally well above 1, is desirable). Higher DOR is desirable.

In conducting the EC runtime prediction matrix across different thresholds and length of rising portion with indoor RH data, we found that a relatively low FPR, high accuracy, high DOR, and a reasonably high number of total predicted data points as EC 'on' could be achieved using a threshold of 2 and no duration threshold, which yielded a false positive rate of only 8%, an accuracy of 67%, and a DOR of 3.53 when applied to true EC on/off data from homes with PLLs on their EC (**Table S2**). Among a total of 335,538 valid data points at 10-minute intervals from PLLs on ECs, the ECs were measured to be on during 121,996 data points (36% true runtime overall). In contrast, this rising indoor RH EC runtime prediction method with these tuning parameters yielded a total of 47,862 data points in which the EC was predicted to be operating (14% predicted runtime). In other words, the method had a high false negative rate of 76% (i.e., low TPR), which means the method only captures approximately 24% of the true EC runtime. However, a low false positive rate of 8% means that when the method predicts the EC is operating,

it is very likely to be actually operating, even though it misses many instances in which the EC is truly operating.

To illustrate further, in the example shown in **Figure S3**, the EC was measured to be operating during 134 of those data points. Among the concurrent 134 indoor RH data, 41 data points (31%) were marked as EC likely operating (rising RH portion) and 93 data points (69%) were not. Importantly, however, no data were marked as potential EC off when the EC was measured to be actually on, while 6 indoor RH data points were marked as EC likely on when the EC was measured to actually be off. Given the success of this method, and because very few of our homes without EC filter interventions had PLLs installed on their ECs, yet given how EC runtime is crucial for contextualizing indoor/outdoor PA data, we rely on a combination of both measured and predicted values to flag times when ECs are operating (or likely operating) for use in conditional analyses herein to avoid biasing our collected data in favor of homes with EC filters. In all cases, priority in the data is given to true values of EC operation as measured by PLL.

Table S2. EC runtime prediction matrix across different thresholds and duration of indoor RH rising portion; the chosen thresholds and duration are highlighted in bold/underline

Threshold	Duration (min)	Measured On	Measured Off	Predicted On	Predicted Off	TPR[1]	FPR[2]	FNR[3]	ACC[4]	DOR[5]
1	0	121996	213542	55244	280294	0.28	0.10	0.72	0.68	3.70
	10	121996	213542	45467	290071	0.24	0.08	0.76	0.67	3.79
	20	121996	213542	29367	306171	0.17	0.04	0.83	0.67	5.17
1.5	0	121996	213542	52219	283319	0.27	0.09	0.73	0.68	3.69
	10	121996	213542	43402	292136	0.23	0.07	0.77	0.67	3.77
	20	121996	213542	28010	307524	0.17	0.04	0.83	0.67	5.18
**2**	**0**	**121996**	**213542**	**47862**	**287676**	**0.24**	**0.08**	**0.76**	**0.67**	**3.53**
	10	121996	213542	39851	295687	0.21	0.07	0.79	0.67	3.62
	20	121996	213542	25751	309787	0.15	0.03	0.85	0.67	5.05
2.5	0	121996	213542	41931	293607	0.21	0.08	0.79	0.66	3.17
	10	121996	213542	34255	301283	0.17	0.06	0.83	0.66	3.25
	20	121996	213542	22041	313497	0.13	0.03	0.87	0.66	4.64
3	0	121996	213542	35645	299893	0.17	0.07	0.83	0.66	2.83
	10	121996	213542	28317	307221	0.14	0.05	0.86	0.65	2.93
	20	121996	213542	17895	317643	0.10	0.03	0.90	0.66	4.30
3.5	0	121996	213542	29596	305942	0.14	0.06	0.86	0.65	2.49
	10	121996	213542	23051	312487	0.11	0.05	0.89	0.65	2.55
	20	121996	213542	14211	321327	0.08	0.02	0.92	0.65	3.85

[1]TPR: True positive rate; [2]TNR: True negative rate; [3]FNR: False negative rate; [4]ACC: Accuracy; [5]DOR: Diagnostic odds ratio

Supplemental Results and Discussion

Pilot year data

Figure S4 shows distributions of raw (i.e., from PAs directly without co-location factors applied) concurrent indoor/outdoor (I/O) $PM_{2.5}$ concentration ratios measured in the pilot year homes at 1-hour average intervals in the 15 days following receipt of one of four types of interventions: a) MERV 13 DIY EC filter, b) indoor PAC with HEPA filter, c) indoor box fan with MERV 13 filter, or d) effectively no intervention (i.e., they received an air cleaner but did not operate it, or declined to receive any intervention). I/O $PM_{2.5}$ ratios are not constrained to ≤1 for these pilot analyses. While there is a small sample size of homes and data available in each group, and the small control group was not an intentional control group by design, homes with DIY EC filters had the lowest median $PM_{2.5}$ I/O ratio (median of medians = 0.56), followed by the indoor HEPA PAC group (median of medians = 1.04), the box fan and filter group (median of medians = 1.05), and the minimal/no intervention group (median of medians = 1.08). It is also worth noting that this analysis does not control for EC operation, and 90% of the 1-hour outdoor $PM_{2.5}$ concentrations as measured by PAs in these locations were below 10 μg/m^3, indicating minimal outdoor concentration events (e.g., no wildfires) during the study period. Nevertheless, the pilot data suggested that the MERV 13 EC filtration solution had promise as a real-world solution for these homes.

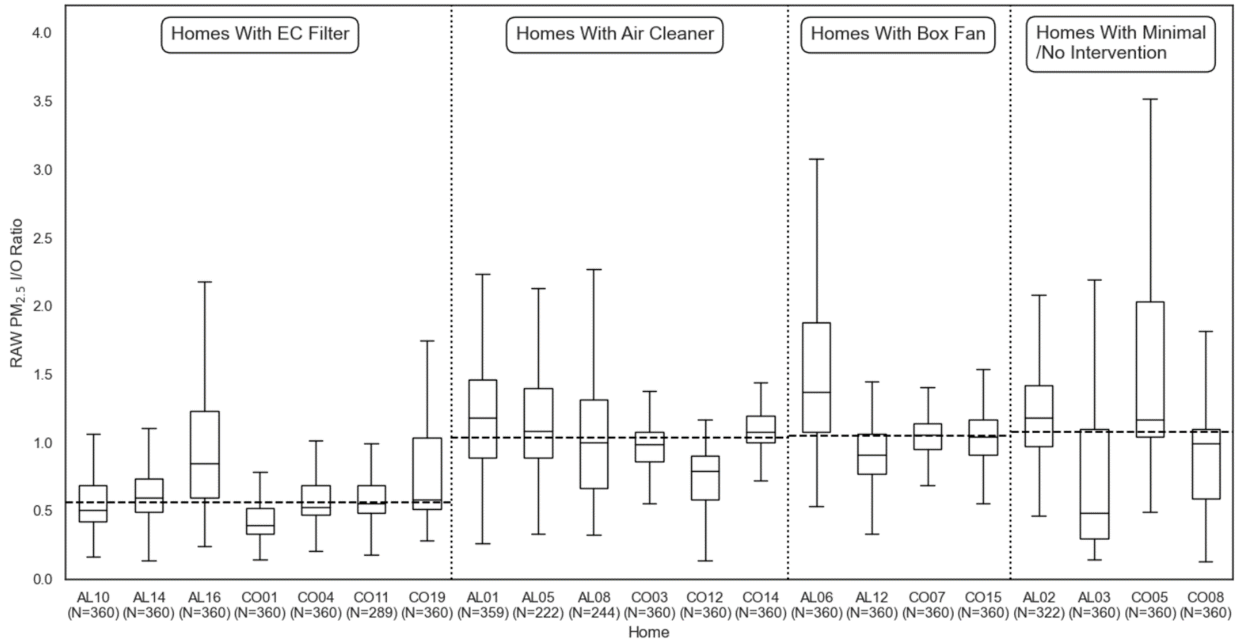

Figure S4. Summary of pilot year (2022) indoor/outdoor (I/O) $PM_{2.5}$ concentration ratios: data from 15 days following intervention, 1-hour average data (the number of data points available for comparison is shown in parentheses)

Full-scale field study

Spot measurements

Figure S5 shows the indoor-outdoor pressure difference across the building envelope measured in 15 homes that had paired measurements with the EC off, the EC operating without a filter, and the EC operating with a new DIY filter installed. The median (IQR) envelope pressure difference increased with the EC operating, as expected, from +0.4 (0.3-0.6) Pa (i.e., approximately neutral) with the EC off to +9.2 (4.7-13.1) Pa with the EC on without an EC filter installed, to +5.6 (2.6-7.6) Pa with the EC on and with an EC filter installed. None of the indoor-outdoor envelope pressure differences were negative with the EC operating with or without the DIY filter. The reduction in pressure difference with EC filters installed is consistent with a reduction in EC airflow rates, yet positive pressurization is still maintained as desired. Therefore, while the EC is operating with a filter installed, these homes are reasonably expected to maintain positive pressurization with respect to the outdoors, which should minimize infiltration of ambient particulate matter through envelope leaks and ensure that most of the incoming outdoor air flows through the EC filter as desired.

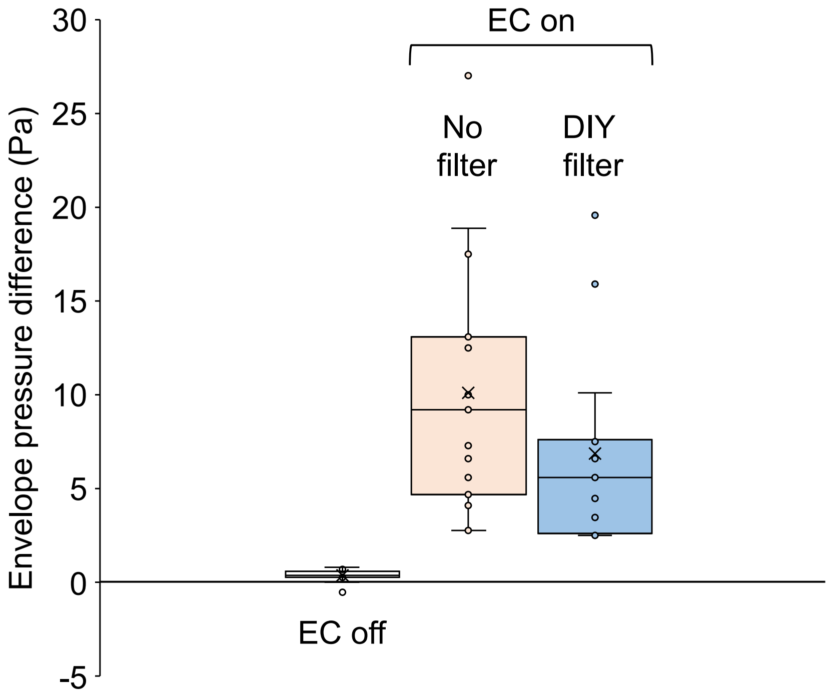

Figure S5. Indoor-outdoor pressure difference measured across the building envelope in 15 homes with the EC off, the EC operating without a filter, and the EC operating with a new DIY filter installed

Long-term measurements

During the post-intervention period in the full-scale field study, there was a total of 271,199 concurrent indoor and outdoor data points across all homes, with a mean (SD) indoor $PM_{2.5}$ _alt concentration, outdoor $PM_{2.5}$ _alt concentration, and I/O $PM_{2.5}$ _alt concentration ratio of 8.3 (24.4) μg/m^3, 7.9 (8.3) μg/m^3, and 1.5 (7.9), respectively. I/O $PM_{2.5}$ concentration ratios were greater than 1 for 34% of the total matched data set, leaving 66% of the data with potential to be analyzed for ambient infiltration in the absence of indoor sources using a constrained I/O ratio. A summary of data presented in **Figure 3** in the main text is provided in **Table S3**, including the number of $PM_{2.5}$_alt data points available for constrained I/O ratios and F_{inf} values during times when the ECs were either measured or predicted to be on or off, as well as summary statistics for those same conditions.

Table S3. Summary of $PM_{2.5}$_alt metrics presented in Figure 3 in the main text

**a) Constrained I/O $PM_{2.5}$ Ratio (-)**									
**Group**	**EC Run Mode[1]**	**Count**	**Mean**	**SD**	**Min**	**25%**	**50%**	**75%**	**Max**
PAC only	EC Off	82411	0.56	0.25	0.00	0.35	0.55	0.79	1.00
	EC On	13218	0.70	0.25	0.02	0.52	0.78	0.91	1.00
PAC + EC filter	EC Off	48007	0.56	0.26	0.00	0.34	0.57	0.78	1.00
	EC On	34645	0.63	0.18	0.02	0.52	0.63	0.75	1.00
**b) $PM_{2.5}$ Infiltration Factor (-)**									
**Group**	**EC Run Mode[1]**	**Count**	**Mean**	**SD**	**Min**	**25%**	**50%**	**75%**	**Max**
PAC only	EC Off	42111	0.56	0.25	0.00	0.35	0.56	0.79	1.00
	EC On	6258	0.68	0.26	0.03	0.50	0.76	0.90	1.00
PAC + EC filter	EC Off	24263	0.55	0.26	0.00	0.34	0.56	0.77	1.00
	EC On	17299	0.62	0.18	0.02	0.51	0.62	0.73	1.00

[1]Includes measured and predicted EC runtime values

Table S4 and **Table S5** expand upon **Table S3** to show the number of data points available for constrained I/O ratios and F_{inf} values, as well as summary statistics, for each home in the full-scale field study.

Table S4. Constrained I/O $PM_{2.5}$ ratio data summary for each home in each intervention group (supporting Figure 3 in the main text)

**Constrained I/O $PM_{2.5}$ Ratio (-)**										
**Group**	**Home**	**EC Run Mode[1]**	**Count**	**Mean**	**SD**	**Min**	**25%**	**50%**	**75%**	**Max**
PAC only	FK07	EC Off	4393	0.55	0.22	0.13	0.37	0.51	0.72	1.00
		EC On	491	0.61	0.22	0.06	0.44	0.61	0.79	1.00
	FK12	EC Off	6263	0.41	0.23	0.00	0.24	0.35	0.52	1.00
		EC On	1215	0.51	0.30	0.02	0.25	0.41	0.85	1.00
	FK13	EC Off	1577	0.59	0.27	0.06	0.39	0.64	0.82	1.00
		EC On	436	0.71	0.21	0.11	0.58	0.75	0.87	1.00
	FK16	EC Off	5434	0.36	0.20	0.02	0.20	0.32	0.45	1.00
		EC On	733	0.77	0.20	0.10	0.68	0.81	0.92	1.00
	FK19	EC Off	4104	0.57	0.21	0.01	0.42	0.53	0.70	1.00
		EC On	521	0.73	0.25	0.16	0.50	0.84	0.97	1.00
	FK25	EC Off	5807	0.74	0.18	0.04	0.63	0.78	0.88	1.00
		EC On	644	0.75	0.17	0.15	0.64	0.77	0.89	1.00
	FK27	EC Off	4083	0.66	0.19	0.06	0.53	0.65	0.82	1.00

		EC On	529	0.73	0.19	0.18	0.59	0.75	0.89	1.00
	FK28	EC Off	6386	0.62	0.27	0.02	0.36	0.69	0.86	1.00
		EC On	1466	0.74	0.19	0.09	0.62	0.80	0.89	1.00
	FK29	EC Off	2521	0.76	0.18	0.02	0.65	0.80	0.90	1.00
		EC On	443	0.76	0.19	0.05	0.64	0.82	0.92	1.00
	FK31	EC Off	3249	0.69	0.20	0.03	0.54	0.71	0.86	1.00
		EC On	434	0.75	0.20	0.10	0.64	0.79	0.91	1.00
	FK34	EC Off	3065	0.81	0.19	0.10	0.72	0.87	0.95	1.00
		EC On	640	0.82	0.18	0.16	0.72	0.88	0.95	1.00
	KE17	EC Off	5963	0.52	0.21	0.02	0.35	0.50	0.68	1.00
		EC On	180	0.68	0.26	0.05	0.47	0.75	0.89	1.00
	KE28	EC Off	5747	0.37	0.21	0.02	0.21	0.33	0.49	1.00
		EC On	964	0.74	0.21	0.04	0.58	0.79	0.93	1.00
	KE30	EC Off	3824	0.78	0.14	0.04	0.69	0.80	0.90	1.00
		EC On	490	0.78	0.15	0.36	0.67	0.81	0.91	1.00
	KE34	EC Off	5544	0.50	0.21	0.01	0.35	0.46	0.63	1.00
		EC On	1608	0.85	0.16	0.08	0.83	0.90	0.96	1.00
	KE35	EC Off	6612	0.59	0.23	0.03	0.38	0.58	0.80	1.00
		EC On	836	0.72	0.23	0.06	0.59	0.81	0.90	1.00
	KE38	EC Off	2555	0.35	0.20	0.03	0.18	0.31	0.45	1.00
		EC On	779	0.26	0.18	0.08	0.14	0.19	0.32	1.00
	KE40	EC Off	5284	0.52	0.25	0.01	0.32	0.49	0.72	1.00
		EC On	809	0.60	0.27	0.03	0.37	0.62	0.84	1.00
PAC + EC filter	FK06	EC Off	2555	0.35	0.20	0.03	0.18	0.31	0.45	1.00
		EC On	779	0.26	0.18	0.08	0.14	0.19	0.32	0.99
	FK08	EC Off	5284	0.52	0.25	0.01	0.32	0.49	0.72	1.00
		EC On	809	0.60	0.27	0.03	0.37	0.62	0.84	1.00
	FK10	EC Off	1424	0.75	0.16	0.10	0.66	0.76	0.87	1.00
		EC On	3924	0.80	0.17	0.07	0.70	0.85	0.92	1.00
	FK14	EC Off	3851	0.73	0.18	0.05	0.65	0.76	0.86	1.00
		EC On	3827	0.58	0.15	0.04	0.50	0.61	0.67	1.00
	FK15	EC Off	4899	0.48	0.20	0.07	0.31	0.43	0.62	1.00
		EC On	2555	0.77	0.15	0.04	0.69	0.78	0.88	1.00
	FK26	EC Off	3358	0.73	0.16	0.14	0.64	0.74	0.85	1.00
		EC On	955	0.67	0.17	0.08	0.56	0.70	0.79	1.00
	FK37	EC Off	2121	0.62	0.19	0.05	0.48	0.62	0.75	1.00
		EC On	5597	0.65	0.15	0.03	0.59	0.68	0.74	1.00
	FK38	EC Off	3994	0.71	0.19	0.09	0.60	0.75	0.86	1.00
		EC On	1292	0.60	0.16	0.08	0.51	0.61	0.70	1.00
	FK39	EC Off	4566	0.38	0.22	0.03	0.20	0.32	0.53	1.00
		EC On	2937	0.53	0.14	0.02	0.47	0.53	0.58	1.00
	KE11	EC Off	978	0.60	0.18	0.04	0.48	0.58	0.71	1.00
		EC On	2471	0.57	0.10	0.14	0.51	0.56	0.62	1.00
	KE19	EC Off	2392	0.45	0.16	0.00	0.34	0.44	0.54	1.00
		EC On	274	0.48	0.14	0.10	0.41	0.46	0.53	0.97
	KE21	EC Off	1611	0.52	0.26	0.02	0.28	0.52	0.74	1.00
		EC On	424	0.76	0.16	0.13	0.63	0.79	0.90	1.00
	KE24	EC Off	3454	0.37	0.15	0.02	0.27	0.35	0.45	1.00
		EC On	1873	0.56	0.17	0.02	0.49	0.55	0.61	1.00
	KE25	EC Off	n/a	n/a	n/a	n/a	n/a	n/a	n/a	n/a
		EC On	180	0.21	0.15	0.05	0.10	0.15	0.32	0.82
	KE33	EC Off	232	0.32	0.20	0.05	0.14	0.35	0.44	0.98
		EC On	3270	0.79	0.14	0.03	0.72	0.81	0.90	1.00

[1]Includes measured and predicted EC runtime values

Table S5. $PM_{2.5}$ infiltration factor (F_{inf}) data summary for each home in each intervention group (supporting Figure 3 in the main text)

**$PM_{2.5}$ Infiltration Factor (F_{inf}) (-)**										
**Group**	**Home**	**EC Run Mode[1]**	**Count**	**Mean**	**SD**	**Min**	**25%**	**50%**	**75%**	**Max**
PAC only	FK07	EC Off	2296	0.55	0.22	0.13	0.37	0.52	0.72	1.00
		EC On	220	0.59	0.23	0.06	0.39	0.58	0.78	1.00
	FK12	EC Off	3096	0.39	0.23	0.00	0.23	0.34	0.50	1.00
		EC On	615	0.49	0.29	0.03	0.24	0.37	0.82	1.00
	FK13	EC Off	739	0.62	0.25	0.09	0.46	0.65	0.82	1.00
		EC On	188	0.69	0.19	0.15	0.56	0.73	0.85	1.00
	FK16	EC Off	2571	0.37	0.21	0.02	0.21	0.32	0.47	1.00
		EC On	360	0.76	0.19	0.10	0.68	0.80	0.91	1.00
	FK19	EC Off	2052	0.59	0.22	0.01	0.42	0.54	0.73	1.00
		EC On	287	0.73	0.25	0.19	0.49	0.86	0.96	1.00
	FK25	EC Off	2931	0.72	0.18	0.04	0.61	0.76	0.86	1.00
		EC On	266	0.74	0.18	0.15	0.60	0.74	0.89	1.00
	FK27	EC Off	2248	0.66	0.19	0.06	0.53	0.64	0.81	1.00
		EC On	225	0.69	0.19	0.18	0.54	0.70	0.85	1.00
	FK28	EC Off	3308	0.62	0.26	0.02	0.38	0.69	0.85	1.00
		EC On	644	0.72	0.20	0.09	0.61	0.80	0.87	1.00
	FK29	EC Off	1434	0.75	0.19	0.02	0.64	0.80	0.90	1.00
		EC On	197	0.75	0.18	0.14	0.64	0.81	0.90	1.00
	FK31	EC Off	1869	0.70	0.21	0.03	0.55	0.72	0.88	1.00
		EC On	233	0.75	0.21	0.10	0.61	0.79	0.91	1.00
	FK34	EC Off	1681	0.81	0.18	0.11	0.72	0.87	0.94	1.00
		EC On	299	0.81	0.18	0.16	0.70	0.87	0.95	1.00
	KE17	EC Off	3053	0.50	0.21	0.02	0.34	0.48	0.66	1.00
		EC On	100	0.67	0.27	0.05	0.47	0.75	0.89	1.00
	KE28	EC Off	2854	0.37	0.21	0.02	0.21	0.33	0.49	1.00
		EC On	488	0.75	0.21	0.04	0.60	0.81	0.93	1.00
	KE30	EC Off	2088	0.77	0.15	0.04	0.67	0.79	0.89	1.00
		EC On	228	0.77	0.16	0.36	0.66	0.79	0.91	1.00
	KE34	EC Off	2625	0.49	0.22	0.01	0.34	0.46	0.63	1.00
		EC On	793	0.84	0.16	0.18	0.81	0.89	0.95	1.00
	KE35	EC Off	3514	0.58	0.23	0.05	0.38	0.58	0.79	1.00
		EC On	367	0.71	0.23	0.06	0.57	0.79	0.89	1.00
	KE38	EC Off	1194	0.33	0.20	0.03	0.18	0.29	0.43	1.00
		EC On	350	0.24	0.17	0.08	0.14	0.18	0.28	0.99
	KE40	EC Off	2558	0.52	0.26	0.01	0.31	0.49	0.74	1.00
		EC On	398	0.57	0.29	0.03	0.32	0.58	0.83	1.00
PAC+EC filter	FK06	EC Off	741	0.75	0.15	0.12	0.66	0.76	0.87	1.00
		EC On	2149	0.79	0.17	0.07	0.68	0.84	0.91	1.00
	FK08	EC Off	1879	0.72	0.18	0.05	0.63	0.75	0.85	1.00
		EC On	1846	0.57	0.15	0.04	0.48	0.59	0.65	1.00
	FK10	EC Off	2351	0.47	0.20	0.07	0.30	0.43	0.61	1.00
		EC On	1226	0.77	0.15	0.04	0.68	0.77	0.88	1.00
	FK14	EC Off	1791	0.72	0.17	0.14	0.63	0.73	0.84	1.00
		EC On	534	0.66	0.17	0.08	0.56	0.69	0.76	1.00
	FK15	EC Off	1150	0.62	0.19	0.05	0.49	0.62	0.76	1.00
		EC On	2823	0.63	0.15	0.03	0.57	0.66	0.72	1.00
	FK26	EC Off	2082	0.70	0.20	0.09	0.59	0.73	0.86	1.00
		EC On	728	0.60	0.16	0.08	0.51	0.61	0.69	1.00
	FK37	EC Off	2365	0.37	0.21	0.03	0.20	0.32	0.51	1.00
		EC On	1439	0.51	0.13	0.02	0.46	0.52	0.57	1.00
	FK38	EC Off	503	0.59	0.19	0.04	0.46	0.58	0.71	1.00

		EC On	1293	0.56	0.10	0.14	0.50	0.55	0.60	1.00
	FK39	EC Off	1156	0.44	0.16	0.00	0.32	0.42	0.52	0.99
		EC On	136	0.45	0.14	0.10	0.38	0.44	0.51	0.89
	KE11	EC Off	848	0.52	0.25	0.02	0.28	0.53	0.73	1.00
		EC On	190	0.75	0.18	0.13	0.61	0.77	0.91	1.00
	KE19	EC Off	1697	0.36	0.15	0.02	0.26	0.33	0.43	0.99
		EC On	907	0.54	0.17	0.02	0.47	0.53	0.59	1.00
	KE21	EC Off	72	0.18	0.11	0.05	0.09	0.15	0.29	0.49
		EC On	113	0.29	0.17	0.05	0.09	0.32	0.42	0.66
	KE24	EC Off	1612	0.78	0.14	0.03	0.70	0.79	0.88	1.00
		EC On	476	0.63	0.12	0.16	0.57	0.62	0.68	1.00
	KE25	EC Off	307	0.65	0.13	0.15	0.56	0.63	0.71	0.98
		EC On	654	0.64	0.16	0.04	0.54	0.61	0.73	1.00
	KE33	EC Off	104	0.14	0.10	0.05	0.07	0.11	0.16	0.65
		EC On	17	0.43	0.07	0.33	0.39	0.43	0.46	0.59
	KE36	EC Off	1615	0.59	0.22	0.03	0.41	0.55	0.77	1.00
		EC On	241	0.62	0.25	0.04	0.41	0.57	0.89	1.00
	KE37	EC Off	1704	0.23	0.19	0.02	0.10	0.16	0.30	0.98
		EC On	950	0.46	0.15	0.02	0.37	0.49	0.56	0.98
	KE39	EC Off	2286	0.58	0.27	0.06	0.34	0.59	0.85	1.00
		EC On	1577	0.64	0.14	0.02	0.57	0.64	0.71	1.00

[1]Includes measured and predicted EC runtime values

Figure S6 shows the same conditional analysis as **Figure 3** in the main text, but only using real EC run mode measured by PLLs. When ECs were known to be operating, the median constrained I/O $PM_{2.5}$ ratio in the PAC only homes was 0.87 compared to 0.39 when ECs were known to be off (an increase of +123%). Conversely, the median constrained I/O $PM_{2.5}$ ratio in the homes with both PAC and EC filters increased from 0.57 with ECs known to be off to 0.63 (+11%) with ECs known to be on. The differences in constrained I/O $PM_{2.5}$ ratios between EC on and EC off conditions had a much larger effect size (d = 1.86) in the PAC only homes compared to homes with both PAC and EC filters (d = 0.36). Similarly, the median $PM_{2.5}$ infiltration factor in the PAC only homes increased from 0.39 with ECs known to be off to 0.87 (+123%) with ECs known to be operating, while the median $PM_{2.5}$ infiltration factor in the PAC+EC filter homes increased from 0.56 with ECs known to be off to 0.62 (+11%) with ECs known to be on. The difference in PAC only homes also had a larger effect size (d = 1.84) than the homes with both PAC and EC filters (d = 0.33). A summary of data presented in **Figure S6** is also provided in **Table S6**, followed by a summary for each home in **Table S7** and **Table S8**. The more drastic differences in both metrics between EC off and on conditions in the PAC only homes using measured EC runtime compared to predicted and/or measured + prediction EC runtime may be attributable to a combination of fewer false negatives in EC on/off signals using true measured data as well as a small number of homes in the PAC only group that had PLLs on ECs.

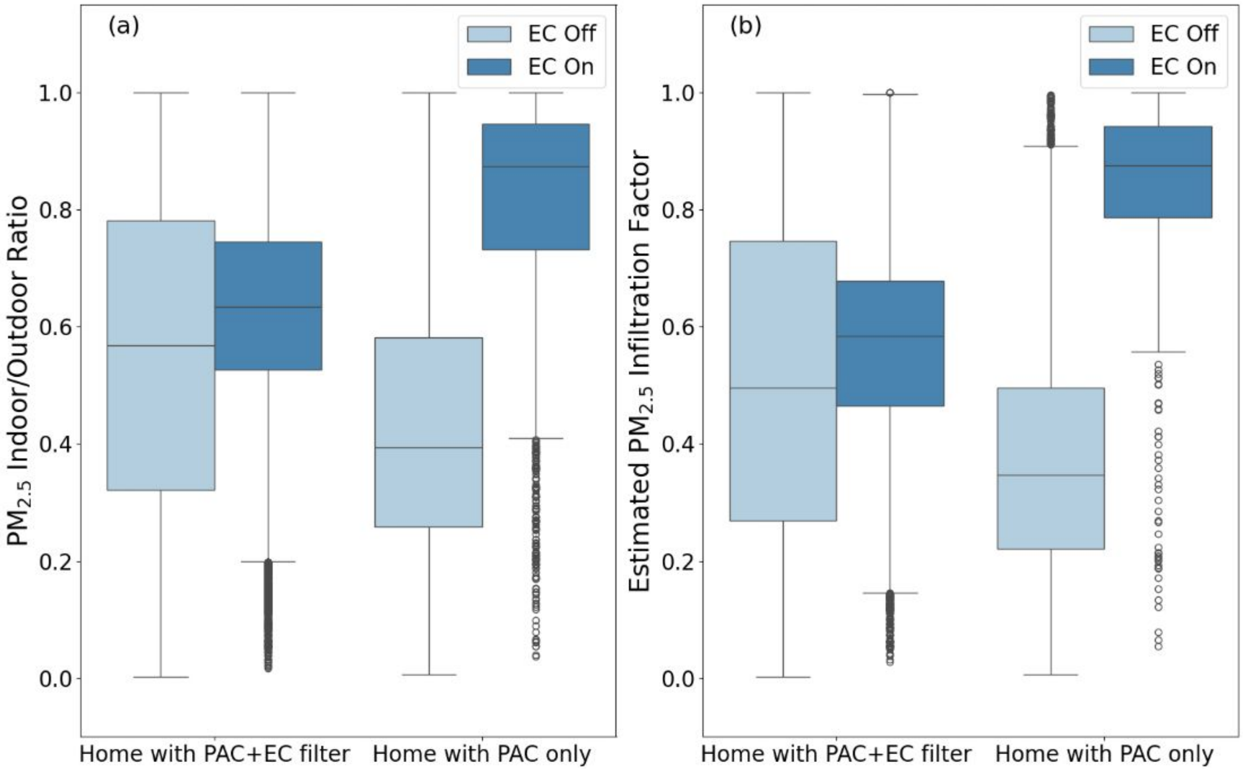

Figure S6. Comparison of (a) $PM_{2.5}$ I/O ratios constrained to I/O ≤ 1 (i.e., excluding indoor sources) and (b) estimated $PM_{2.5}$ infiltration factor between homes with PAC + EC filter homes with PAC only, conditionally comparing only when ECs were measured to be on to when they were measured to be off, using all available post-intervention data

Table S6. Summary of $PM_{2.5}$_alt metrics presented in Figure S6

**a) Constrained I/O $PM_{2.5}$ Ratio (-)**									
**Group**	**EC Run Mode[1]**	**Count**	**Mean**	**SD**	**Min**	**25%**	**50%**	**75%**	**Max**
PAC only	EC Off	21893	0.43	0.22	0.01	0.26	0.39	0.58	1.00
	EC On	3328	0.81	0.19	0.04	0.73	0.87	0.95	1.00
PAC + EC filter	EC Off	42352	0.55	0.26	0.00	0.32	0.57	0.78	1.00
	EC On	33547	0.63	0.18	0.02	0.53	0.63	0.74	1.00
**b) $PM_{2.5}$ Infiltration Factor (-)**									
**Group**	**EC Run Mode[1]**	**Count**	**Mean**	**SD**	**Min**	**25%**	**50%**	**75%**	**Max**
PAC only	EC Off	10779	0.43	0.22	0.01	0.26	0.39	0.57	1.00
	EC On	1677	0.80	0.19	0.04	0.73	0.87	0.94	1.00
PAC + EC filter	EC Off	21258	0.55	0.26	0.00	0.32	0.56	0.77	1.00
	EC On	16790	0.62	0.18	0.02	0.51	0.62	0.73	1.00

[1]Includes only measured EC runtime values

Table S7. Constrained I/O $PM_{2.5}$ ratio data summary for each home in each intervention group in Figure S6

Constrained I/O $PM_{2.5}$ Ratio (-)										
**Group**	**Home**	**EC Run Mode[1]**	**Count**	**Mean**	**SD**	**Min**	**25%**	**50%**	**75%**	**Max**
PAC only	FK16	EC Off	5434	0.36	0.20	0.02	0.20	0.32	0.45	1.00
		EC On	733	0.77	0.20	0.10	0.68	0.81	0.92	1.00
	KE17	EC Off	5963	0.52	0.21	0.02	0.35	0.50	0.68	1.00
		EC On	180	0.68	0.26	0.05	0.47	0.75	0.89	1.00
	KE28	EC Off	5747	0.37	0.21	0.02	0.21	0.33	0.49	1.00
		EC On	964	0.74	0.21	0.04	0.58	0.79	0.93	1.00
	KE34	EC Off	3323	0.47	0.21	0.01	0.30	0.45	0.65	1.00
		EC On	1339	0.89	0.10	0.15	0.85	0.91	0.96	1.00
	KE35	EC Off	1426	0.49	0.22	0.09	0.31	0.44	0.66	1.00
		EC On	112	0.89	0.12	0.06	0.86	0.93	0.96	1.00
PAC+EC filter	FK06	EC Off	1424	0.75	0.16	0.10	0.66	0.76	0.87	1.00
		EC On	3924	0.80	0.17	0.07	0.70	0.85	0.92	1.00
	FK08	EC Off	3851	0.73	0.18	0.05	0.65	0.76	0.86	1.00
		EC On	3827	0.58	0.15	0.04	0.50	0.61	0.67	1.00
	FK10	EC Off	4899	0.48	0.20	0.07	0.31	0.43	0.62	1.00
		EC On	2555	0.77	0.15	0.04	0.69	0.78	0.88	1.00
	FK14	EC Off	3358	0.73	0.16	0.14	0.64	0.74	0.85	1.00
		EC On	955	0.67	0.17	0.08	0.56	0.70	0.79	1.00
	FK15	EC Off	2121	0.62	0.19	0.05	0.48	0.62	0.75	1.00
		EC On	5597	0.65	0.15	0.03	0.59	0.68	0.74	1.00
	FK26	EC Off	3994	0.71	0.19	0.09	0.60	0.75	0.86	1.00
		EC On	1292	0.60	0.16	0.08	0.51	0.61	0.70	1.00
	FK37	EC Off	4566	0.38	0.22	0.03	0.20	0.32	0.53	1.00
		EC On	2937	0.53	0.14	0.02	0.47	0.53	0.58	1.00
	FK38	EC Off	978	0.60	0.18	0.04	0.48	0.58	0.71	1.00
		EC On	2471	0.57	0.10	0.14	0.51	0.56	0.62	1.00
	FK39	EC Off	1394	0.44	0.18	0.00	0.32	0.42	0.53	1.00
		EC On	87	0.50	0.19	0.10	0.38	0.48	0.62	0.97
	KE11	EC Off	458	0.38	0.23	0.05	0.20	0.29	0.53	1.00
		EC On	112	0.87	0.14	0.18	0.83	0.92	0.97	1.00
	KE19	EC Off	3454	0.37	0.15	0.02	0.27	0.35	0.45	1.00
		EC On	1873	0.56	0.17	0.02	0.49	0.55	0.61	1.00
	KE21	EC Off	180	0.21	0.15	0.05	0.10	0.15	0.32	0.82
		EC On	232	0.32	0.20	0.05	0.14	0.35	0.44	0.98
	KE24	EC Off	3270	0.79	0.14	0.03	0.72	0.81	0.90	1.00
		EC On	1156	0.64	0.11	0.09	0.59	0.63	0.70	1.00
	KE25	EC Off	n/a	n/a	n/a	n/a	n/a	n/a	n/a	n/a
		EC On	1173	0.66	0.15	0.04	0.56	0.64	0.76	1.00
	KE33	EC Off	223	0.14	0.09	0.05	0.08	0.11	0.16	0.65
		EC On	51	0.45	0.07	0.33	0.39	0.43	0.49	0.64
	KE37	EC Off	3523	0.23	0.20	0.02	0.10	0.16	0.30	1.00
		EC On	2171	0.48	0.15	0.02	0.39	0.50	0.58	0.99
	KE39	EC Off	4659	0.57	0.28	0.04	0.32	0.54	0.86	1.00
		EC On	3134	0.67	0.14	0.02	0.59	0.66	0.74	1.00

[1]Includes only measured EC runtime values

Table S8. $PM_{2.5}$ infiltration factor (F_{inf}) data summary for each home in each intervention group in Figure S6

**$PM_{2.5}$ Infiltration Factor (F_{inf}) (-)**										
**Group**	**Home**	**EC Run Mode[1]**	**Count**	**Mean**	**SD**	**Min**	**25%**	**50%**	**75%**	**Max**
PAC only	FK16	EC Off	2571	0.37	0.21	0.02	0.21	0.32	0.47	1.00
		EC On	360	0.76	0.19	0.10	0.68	0.80	0.91	1.00
	KE17	EC Off	3053	0.50	0.21	0.02	0.34	0.48	0.66	1.00
		EC On	100	0.67	0.27	0.05	0.47	0.75	0.89	1.00
	KE28	EC Off	2854	0.37	0.21	0.02	0.21	0.33	0.49	1.00
		EC On	488	0.75	0.21	0.04	0.60	0.81	0.93	1.00
	KE34	EC Off	1568	0.46	0.21	0.01	0.29	0.43	0.63	1.00
		EC On	676	0.88	0.11	0.27	0.84	0.90	0.95	1.00
	KE35	EC Off	733	0.48	0.21	0.09	0.30	0.43	0.65	1.00
		EC On	53	0.87	0.15	0.06	0.87	0.92	0.95	0.99
PAC+EC filter	FK06	EC Off	741	0.75	0.15	0.12	0.66	0.76	0.87	1.00
		EC On	2149	0.79	0.17	0.07	0.68	0.84	0.91	1.00
	FK08	EC Off	1879	0.72	0.18	0.05	0.63	0.75	0.85	1.00
		EC On	1846	0.57	0.15	0.04	0.48	0.59	0.65	1.00
	FK10	EC Off	2351	0.47	0.20	0.07	0.30	0.43	0.61	1.00
		EC On	1226	0.77	0.15	0.04	0.68	0.77	0.88	1.00
	FK14	EC Off	1791	0.72	0.17	0.14	0.63	0.73	0.84	1.00
		EC On	534	0.66	0.17	0.08	0.56	0.69	0.76	1.00
	FK15	EC Off	1150	0.62	0.19	0.05	0.49	0.62	0.76	1.00
		EC On	2823	0.63	0.15	0.03	0.57	0.66	0.72	1.00
	FK26	EC Off	2082	0.70	0.20	0.09	0.59	0.73	0.86	1.00
		EC On	728	0.60	0.16	0.08	0.51	0.61	0.69	1.00
	FK37	EC Off	2365	0.37	0.21	0.03	0.20	0.32	0.51	1.00
		EC On	1439	0.51	0.13	0.02	0.46	0.52	0.57	1.00
	FK38	EC Off	503	0.59	0.19	0.04	0.46	0.58	0.71	1.00
		EC On	1293	0.56	0.10	0.14	0.50	0.55	0.60	1.00
	FK39	EC Off	663	0.43	0.18	0.00	0.31	0.41	0.52	0.99
		EC On	43	0.44	0.19	0.10	0.31	0.43	0.56	0.89
	KE11	EC Off	258	0.40	0.24	0.05	0.19	0.32	0.57	1.00
		EC On	61	0.89	0.14	0.18	0.85	0.93	0.97	1.00
	KE19	EC Off	1697	0.36	0.15	0.02	0.26	0.33	0.43	0.99
		EC On	907	0.54	0.17	0.02	0.47	0.53	0.59	1.00
	KE21	EC Off	72	0.18	0.11	0.05	0.09	0.15	0.29	0.49
		EC On	113	0.29	0.17	0.05	0.09	0.32	0.42	0.66
	KE24	EC Off	1612	0.78	0.14	0.03	0.70	0.79	0.88	1.00
		EC On	476	0.63	0.12	0.16	0.57	0.62	0.68	1.00
	KE25	EC Off	n/a	n/a	n/a	n/a	n/a	n/a	n/a	n/a
		EC On	608	0.65	0.16	0.04	0.54	0.63	0.75	1.00
	KE33	EC Off	104	0.14	0.10	0.05	0.07	0.11	0.16	0.65
		EC On	17	0.43	0.07	0.33	0.39	0.43	0.46	0.59
	KE37	EC Off	1704	0.23	0.19	0.02	0.10	0.16	0.30	0.98
		EC On	950	0.46	0.15	0.02	0.37	0.49	0.56	0.98
	KE39	EC Off	2286	0.58	0.27	0.06	0.34	0.59	0.85	1.00
		EC On	1577	0.64	0.14	0.02	0.57	0.64	0.71	1.00

[1]Includes only measured EC runtime values

Similarly, **Figure S7** uses only predicted EC run mode for the same conditional analysis as **Figure 3** (main text) and **Figure S6** (SI). When ECs were predicted to be operating, the median constrained I/O $PM_{2.5}$ ratio in the PAC only homes was 0.87 compared to 0.39 when ECs were predicted to be off (an increase of +123%). Conversely, the median constrained I/O $PM_{2.5}$ ratio in the homes with both PAC and EC filters increased from 0.57 with ECs predicted to be off to 0.63 (+11%) with ECs predicted to be on. The differences in constrained I/O $PM_{2.5}$ ratios between predicted EC on and EC off conditions much larger effect size (d = 1.86) in PAC only homes than homes with both PAC and EC filters (d = 0.36). Similarly, the median $PM_{2.5}$ infiltration factor in the PAC only homes increased from 0.39 with ECs predicted to be off to 0.87 (+123%) with ECs predicted to be operating, while the median $PM_{2.5}$ infiltration factor in the PAC+EC filter homes increased from 0.56 with ECs predicted to be off to 0.62 (+11%) with ECs predicted to be operating. Again, the difference in PAC only homes had a much larger effect size (d = 1.84) than homes with both PAC and EC filters (d = 0.33). A summary of data presented in **Figure S7** is also provided in **Table S9**, followed by a summary for each home in **Table S10** and **Table S11**.

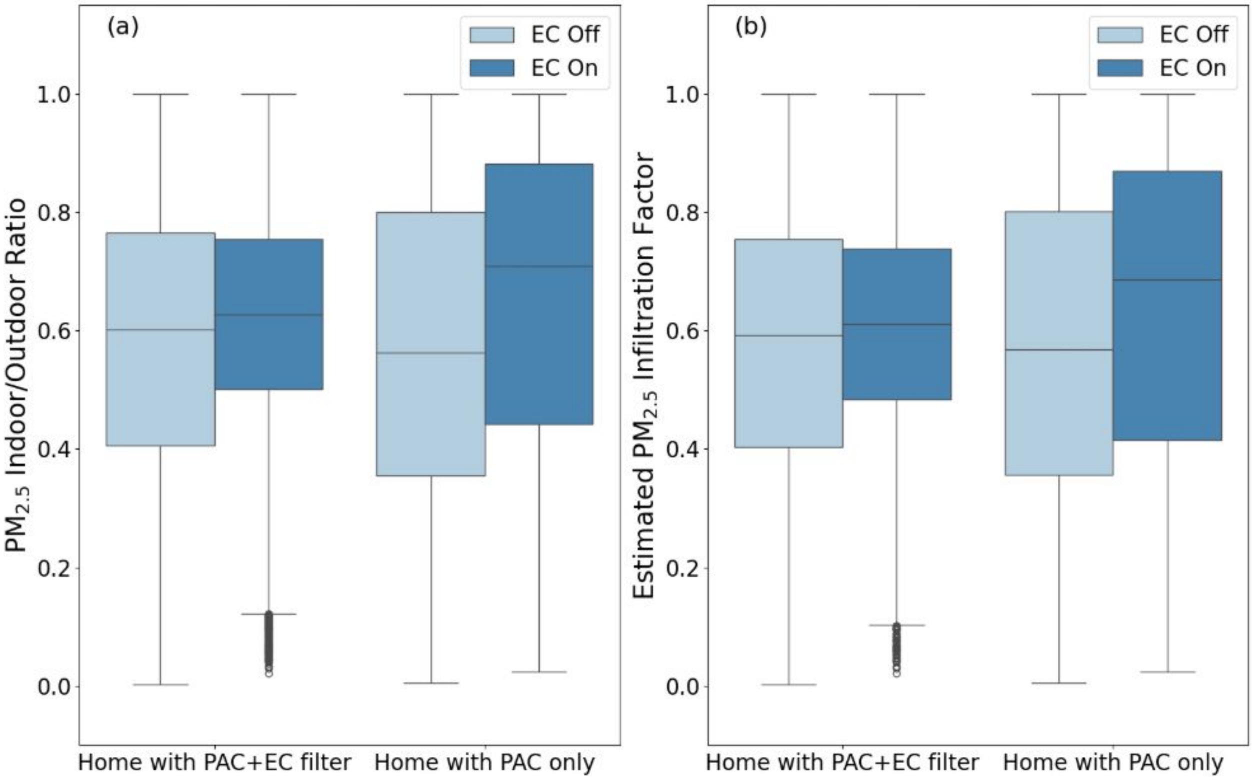

Figure S7. Comparison of (a) $PM_{2.5}$ I/O ratios constrained to I/O ≤ 1 (i.e., excluding indoor sources) and (b) estimated $PM_{2.5}$ infiltration factor between homes with PAC + EC filter homes with PAC only, conditionally comparing only when ECs were <u>predicted</u> to be on to when they were <u>predicted</u> to be off, using all available post-intervention data

Table S9. Summary of $PM_{2.5}$_alt metrics presented in Figure S7

**a) Constrained I/O $PM_{2.5}$ Ratio (-)**									
**Group**	**EC Run Mode[1]**	**Count**	**Mean**	**SD**	**Min**	**25%**	**50%**	**75%**	**Max**
PAC only	EC Off	83140	0.57	0.26	0.00	0.36	0.56	0.80	1.00
	EC On	12489	0.65	0.26	0.02	0.44	0.71	0.88	1.00
PAC + EC Filter	EC Off	68285	0.58	0.24	0.00	0.41	0.60	0.76	1.00
	EC On	14367	0.62	0.20	0.02	0.50	0.63	0.75	1.00
**b) $PM_{2.5}$ Infiltration Factor (-)**									
**Group**	**EC Run Mode[1]**	**Count**	**Mean**	**SD**	**Min**	**25%**	**50%**	**75%**	**Max**
PAC only	EC Off	42638	0.57	0.26	0.00	0.36	0.57	0.80	1.00
	EC On	5731	0.63	0.27	0.02	0.42	0.69	0.87	1.00
PAC + EC filter	EC Off	34857	0.58	0.23	0.00	0.40	0.59	0.75	1.00
	EC On	6705	0.60	0.20	0.02	0.48	0.61	0.74	1.00

[1]Includes only predicted EC runtime values

Table S10. Constrained I/O $PM_{2.5}$ ratio data summary for each home in each intervention group in Figure S7

**Constrained I/O $PM_{2.5}$ Ratio (-)**										
**Group**	**Home**	**EC Run Mode[1]**	**Count**	**Mean**	**SD**	**Min**	**25%**	**50%**	**75%**	**Max**
PAC only	FK07	EC Off	4393	0.55	0.22	0.13	0.37	0.51	0.72	1.00
		EC On	491	0.61	0.22	0.06	0.44	0.61	0.79	1.00
	FK13	EC Off	6263	0.41	0.23	0.00	0.24	0.35	0.52	1.00
		EC On	1215	0.51	0.30	0.02	0.25	0.41	0.85	1.00
	FK16	EC Off	1577	0.59	0.27	0.06	0.39	0.64	0.82	1.00
		EC On	436	0.71	0.21	0.11	0.58	0.75	0.87	1.00
	FK19	EC Off	5719	0.39	0.23	0.02	0.21	0.33	0.50	1.00
		EC On	448	0.63	0.27	0.11	0.41	0.69	0.86	1.00
	FK25	EC Off	4104	0.57	0.21	0.01	0.42	0.53	0.70	1.00
		EC On	521	0.73	0.25	0.16	0.50	0.84	0.97	1.00
	FK27	EC Off	5807	0.74	0.18	0.04	0.63	0.78	0.88	1.00
		EC On	644	0.75	0.17	0.15	0.64	0.77	0.89	1.00
	FK28	EC Off	4083	0.66	0.19	0.06	0.53	0.65	0.82	1.00
		EC On	529	0.73	0.19	0.18	0.59	0.75	0.89	1.00
	FK31	EC Off	6386	0.62	0.27	0.02	0.36	0.69	0.86	1.00
		EC On	1466	0.74	0.19	0.09	0.62	0.80	0.89	1.00
	FK34	EC Off	2521	0.76	0.18	0.02	0.65	0.80	0.90	1.00
		EC On	443	0.76	0.19	0.05	0.64	0.82	0.92	1.00
	KE17	EC Off	3249	0.69	0.20	0.03	0.54	0.71	0.86	1.00
		EC On	434	0.75	0.20	0.10	0.64	0.79	0.91	1.00
	KE28	EC Off	3065	0.81	0.19	0.10	0.72	0.87	0.95	1.00
		EC On	640	0.82	0.18	0.16	0.72	0.88	0.95	1.00
	KE30	EC Off	5564	0.52	0.21	0.02	0.35	0.50	0.68	1.00
		EC On	579	0.56	0.23	0.04	0.38	0.56	0.74	0.99
	KE34	EC Off	5994	0.41	0.24	0.02	0.22	0.36	0.55	1.00
		EC On	717	0.55	0.29	0.02	0.29	0.55	0.83	1.00
	KE35	EC Off	3824	0.78	0.14	0.04	0.69	0.80	0.90	1.00
		EC On	490	0.78	0.15	0.36	0.67	0.81	0.91	1.00
	KE38	EC Off	6217	0.55	0.24	0.01	0.37	0.51	0.76	1.00
		EC On	935	0.73	0.23	0.04	0.54	0.81	0.93	1.00
	KE40	EC Off	6535	0.60	0.23	0.03	0.39	0.60	0.81	1.00
		EC On	913	0.67	0.24	0.09	0.46	0.74	0.88	1.00
PAC + EC filter	FK06	EC Off	2296	0.55	0.22	0.13	0.37	0.52	0.72	1.00
		EC On	220	0.59	0.23	0.06	0.39	0.58	0.78	1.00
	FK08	EC Off	3096	0.39	0.23	0.00	0.23	0.34	0.50	1.00
		EC On	615	0.49	0.29	0.03	0.24	0.37	0.82	1.00
	FK10	EC Off	739	0.62	0.25	0.09	0.46	0.65	0.82	1.00

		EC On	188	0.69	0.19	0.15	0.56	0.73	0.85	1.00
	FK14	EC Off	2733	0.40	0.23	0.02	0.22	0.34	0.53	1.00
		EC On	198	0.64	0.26	0.11	0.43	0.70	0.87	1.00
	FK15	EC Off	2052	0.59	0.22	0.01	0.42	0.54	0.73	1.00
		EC On	287	0.73	0.25	0.19	0.49	0.86	0.96	1.00
	FK26	EC Off	2931	0.72	0.18	0.04	0.61	0.76	0.86	1.00
		EC On	266	0.74	0.18	0.15	0.60	0.74	0.89	1.00
	FK37	EC Off	2248	0.66	0.19	0.06	0.53	0.64	0.81	1.00
		EC On	225	0.69	0.19	0.18	0.54	0.70	0.85	1.00
	FK38	EC Off	3308	0.62	0.26	0.02	0.38	0.69	0.85	1.00
		EC On	644	0.72	0.20	0.09	0.61	0.80	0.87	1.00
	FK39	EC Off	1434	0.75	0.19	0.02	0.64	0.80	0.90	1.00
		EC On	197	0.75	0.18	0.14	0.64	0.81	0.90	1.00
	KE11	EC Off	1869	0.70	0.21	0.03	0.55	0.72	0.88	1.00
		EC On	233	0.75	0.21	0.10	0.61	0.79	0.91	1.00
	KE19	EC Off	1681	0.81	0.18	0.11	0.72	0.87	0.94	1.00
		EC On	299	0.81	0.18	0.16	0.70	0.87	0.95	1.00
	KE21	EC Off	2897	0.51	0.21	0.02	0.34	0.48	0.66	1.00
		EC On	256	0.54	0.23	0.04	0.37	0.54	0.70	0.99
	KE24	EC Off	3033	0.41	0.24	0.02	0.22	0.36	0.55	1.00
		EC On	309	0.54	0.30	0.02	0.24	0.55	0.82	1.00
	KE25	EC Off	n/a	n/a	n/a	n/a	n/a	n/a	n/a	n/a
		EC On	2088	0.77	0.15	0.04	0.67	0.79	0.89	1.00
	KE33	EC Off	228	0.77	0.16	0.36	0.66	0.79	0.91	1.00
		EC On	2992	0.56	0.25	0.01	0.36	0.51	0.78	1.00

[1]Includes only predicted EC runtime values

Table S11. $PM_{2.5}$ infiltration factor (F_{inf}) data summary for each home in each intervention group in Figure S7

**$PM_{2.5}$ Infiltration Factor (F_{inf}) (-)**										
**Group**	**Home**	**EC Run Mode[1]**	**Count**	**Mean**	**SD**	**Min**	**25%**	**50%**	**75%**	**Max**
PAC only	FK07	EC Off	2296	0.55	0.22	0.13	0.37	0.52	0.72	1.00
		EC On	220	0.59	0.23	0.06	0.39	0.58	0.78	1.00
	FK12	EC Off	3096	0.39	0.23	0.00	0.23	0.34	0.50	1.00
		EC On	615	0.49	0.29	0.03	0.24	0.37	0.82	1.00
	FK13	EC Off	739	0.62	0.25	0.09	0.46	0.65	0.82	1.00
		EC On	188	0.69	0.19	0.15	0.56	0.73	0.85	1.00
	FK16	EC Off	2733	0.40	0.23	0.02	0.22	0.34	0.53	1.00
		EC On	198	0.64	0.26	0.11	0.43	0.70	0.87	1.00
	FK19	EC Off	2052	0.59	0.22	0.01	0.42	0.54	0.73	1.00
		EC On	287	0.73	0.25	0.19	0.49	0.86	0.96	1.00
	FK25	EC Off	2931	0.72	0.18	0.04	0.61	0.76	0.86	1.00
		EC On	266	0.74	0.18	0.15	0.60	0.74	0.89	1.00
	FK27	EC Off	2248	0.66	0.19	0.06	0.53	0.64	0.81	1.00
		EC On	225	0.69	0.19	0.18	0.54	0.70	0.85	1.00
	FK28	EC Off	3308	0.62	0.26	0.02	0.38	0.69	0.85	1.00
		EC On	644	0.72	0.20	0.09	0.61	0.80	0.87	1.00
	FK29	EC Off	1434	0.75	0.19	0.02	0.64	0.80	0.90	1.00
		EC On	197	0.75	0.18	0.14	0.64	0.81	0.90	1.00
	FK31	EC Off	1869	0.70	0.21	0.03	0.55	0.72	0.88	1.00
		EC On	233	0.75	0.21	0.10	0.61	0.79	0.91	1.00
	FK34	EC Off	1681	0.81	0.18	0.11	0.72	0.87	0.94	1.00
		EC On	299	0.81	0.18	0.16	0.70	0.87	0.95	1.00
	KE17	EC Off	2897	0.51	0.21	0.02	0.34	0.48	0.66	1.00
		EC On	256	0.54	0.23	0.04	0.37	0.54	0.70	0.99

	KE28	EC Off	3033	0.41	0.24	0.02	0.22	0.36	0.55	1.00
		EC On	309	0.54	0.30	0.02	0.24	0.55	0.82	1.00
	KE30	EC Off	2088	0.77	0.15	0.04	0.67	0.79	0.89	1.00
		EC On	228	0.77	0.16	0.36	0.66	0.79	0.91	1.00
	KE34	EC Off	2992	0.56	0.25	0.01	0.36	0.51	0.78	1.00
		EC On	426	0.69	0.24	0.04	0.49	0.75	0.90	1.00
	KE35	EC Off	3489	0.59	0.23	0.05	0.38	0.59	0.80	1.00
		EC On	392	0.65	0.24	0.09	0.45	0.72	0.85	1.00
	KE38	EC Off	1194	0.33	0.20	0.03	0.18	0.29	0.43	1.00
		EC On	350	0.24	0.17	0.08	0.14	0.18	0.28	0.99
	KE40	EC Off	2558	0.52	0.26	0.01	0.31	0.49	0.74	1.00
		EC On	398	0.57	0.29	0.03	0.32	0.58	0.83	1.00
PAC+EC filter	FK06	EC Off	2407	0.78	0.17	0.07	0.67	0.82	0.91	1.00
		EC On	483	0.77	0.18	0.08	0.67	0.83	0.91	1.00
	FK08	EC Off	3064	0.65	0.18	0.04	0.54	0.66	0.79	1.00
		EC On	661	0.60	0.17	0.09	0.51	0.61	0.68	1.00
	FK10	EC Off	3170	0.56	0.23	0.04	0.34	0.54	0.75	1.00
		EC On	407	0.68	0.20	0.17	0.56	0.71	0.82	1.00
	FK14	EC Off	1885	0.71	0.17	0.08	0.62	0.72	0.83	1.00
		EC On	440	0.69	0.17	0.15	0.60	0.70	0.81	1.00
	FK15	EC Off	3121	0.63	0.16	0.03	0.53	0.66	0.73	1.00
		EC On	852	0.63	0.15	0.11	0.57	0.66	0.72	1.00
	FK26	EC Off	2378	0.68	0.20	0.08	0.56	0.70	0.83	1.00
		EC On	432	0.66	0.18	0.12	0.55	0.65	0.79	1.00
	FK37	EC Off	3345	0.42	0.20	0.03	0.24	0.42	0.55	1.00
		EC On	459	0.48	0.15	0.02	0.42	0.49	0.54	0.98
	FK38	EC Off	1425	0.57	0.14	0.04	0.49	0.55	0.62	1.00
		EC On	371	0.56	0.10	0.14	0.50	0.55	0.61	0.99
	FK39	EC Off	1108	0.43	0.16	0.00	0.32	0.42	0.52	0.99
		EC On	184	0.47	0.13	0.25	0.39	0.45	0.51	0.94
	KE11	EC Off	871	0.54	0.26	0.02	0.30	0.56	0.76	1.00
		EC On	167	0.68	0.21	0.13	0.57	0.73	0.83	1.00
	KE19	EC Off	2247	0.42	0.18	0.02	0.28	0.40	0.52	1.00
		EC On	357	0.45	0.17	0.03	0.32	0.48	0.56	0.99
	KE21	EC Off	153	0.21	0.15	0.05	0.08	0.15	0.34	0.66
		EC On	32	0.40	0.11	0.13	0.36	0.40	0.46	0.61
	KE24	EC Off	1703	0.76	0.15	0.03	0.67	0.77	0.87	1.00
		EC On	385	0.68	0.14	0.16	0.59	0.66	0.77	1.00
	KE25	EC Off	821	0.66	0.15	0.15	0.56	0.64	0.75	1.00
		EC On	140	0.56	0.14	0.04	0.50	0.55	0.61	0.98
	KE33	EC Off	107	0.15	0.12	0.05	0.07	0.11	0.16	0.65
		EC On	14	0.40	0.13	0.14	0.33	0.40	0.49	0.59
	KE36	EC Off	1615	0.59	0.22	0.03	0.41	0.55	0.77	1.00
		EC On	241	0.62	0.25	0.04	0.41	0.57	0.89	1.00
	KE37	EC Off	2247	0.30	0.21	0.02	0.12	0.24	0.47	0.98
		EC On	407	0.38	0.21	0.04	0.18	0.39	0.55	0.96
	KE39	EC Off	3190	0.60	0.23	0.02	0.39	0.62	0.78	1.00
		EC On	673	0.65	0.19	0.12	0.55	0.66	0.78	1.00

[1]Includes only predicted EC runtime values

Additionally, **Figure S8** shows the same conditional analysis as **Figure 3** in the main text, but instead using the mean results from each home as independent observations rather than the much larger data set and its dependent observations. When ECs were known or predicted to be operating, the median of the mean constrained I/O $PM_{2.5}$ ratio in the PAC only homes was 0.73 compared to 0.58 when ECs were known or predicted to be off (a median increase of +25%). Conversely, the median of the mean constrained I/O $PM_{2.5}$ ratio in the homes with both PAC and EC filters increased from 0.58 with ECs known or predicted to be off to 0.62 with ECs known or predicted to be on (a median increase of +7%). Similarly, the median of the mean $PM_{2.5}$ infiltration factors in the PAC only homes increased from 0.59 with ECs known or predicted to be off to 0.73 with ECs known or predicted to be operating (a median increase of +24%), while the median of the mean $PM_{2.5}$ infiltration factors in the PAC+EC filter homes increased from 0.58 with ECs known or predicted to be off to 0.61 with ECs known or predicted to be operating (a median increase of +5%). Mann-Whitney U tests indicated that both the distributions of mean constrained I/O $PM_{2.5}$ ratios and estimated $PM_{2.5}$ infiltration factors differed significantly between EC on and EC off conditions in homes with PAC only (p=0.013, *d*=0.84 for constrained I/O $PM_{2.5}$ ratio and p=0.028, *d*=0.75 for F_{inf}), while the differences between EC on and EC off conditions in homes with PAC and EC filters were not significant (p=0.37, *d*=0.44 for constrained I/O $PM_{2.5}$ ratio and p=0.44, *d*=0.37 for F_{inf}). A summary of data in **Figure S8** is also provided in **Table S**12**.**

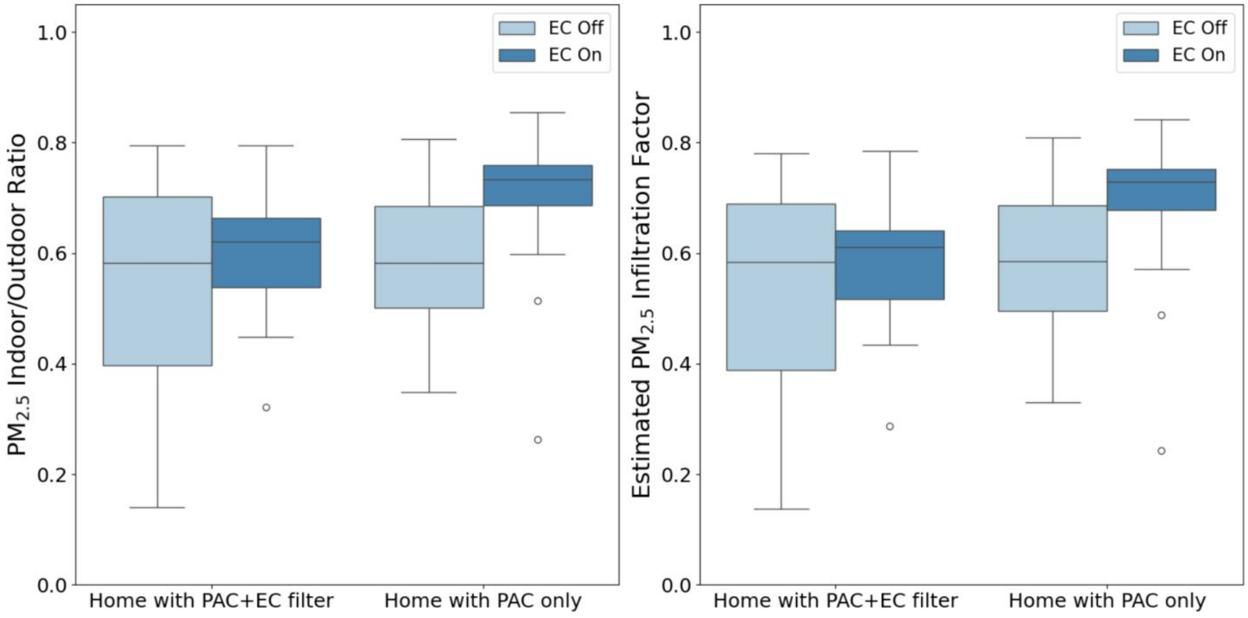

Figure S8. Comparison of distributions of home mean values of (a) $PM_{2.5}$ I/O ratios constrained to I/O < 1 (i.e., excluding indoor sources) and (b) estimated $PM_{2.5}$ infiltration factors between homes with PAC + EC filter homes with PAC only, conditionally comparing when ECs were known or predicted to be on to when they were known or predicted to be off, using all available post-intervention data.

Table S12. Summary of home average $PM_{2.5}$_alt infiltration metrics presented in Figure S8

**a) Constrained I/O $PM_{2.5}$ Ratio (-)**									
**Group**	**EC Run Mode[1]**	**Count**	**Mean**	**SD**	**Min**	**25%**	**50%**	**75%**	**Max**
PAC only	EC Off	18	0.58	0.15	0.35	0.50	0.58	0.68	0.81
	EC On	18	0.70	0.13	0.26	0.69	0.73	0.76	0.85
PAC + EC filter	EC Off	18	0.53	0.20	0.14	0.40	0.58	0.70	0.79
	EC On	18	0.60	0.12	0.32	0.54	0.62	0.66	0.80
**b) $PM_{2.5}$ Infiltration Factor (-)**									
**Group**	**EC Run Mode[1]**	**Count**	**Mean**	**SD**	**Min**	**25%**	**50%**	**75%**	**Max**
PAC only	EC Off	18	0.57	0.15	0.33	0.50	0.59	0.69	0.81
	EC On	18	0.68	0.14	0.24	0.68	0.73	0.75	0.84
PAC + EC filter	EC Off	18	0.52	0.20	0.14	0.39	0.58	0.69	0.78
	EC On	18	0.58	0.13	0.29	0.52	0.61	0.64	0.79

[1]Includes measured and predicted EC runtime values

Wildfire vs. non-wildfire periods

During the field campaign in 2023, there were two distinct wildfire events around the participating communities during the post-intervention period: August 22-24, 2023, and September 21-25, 2023, totaling approximately eight days. A comparison of outdoor $PM_{2.5}$_alt concentrations measured between the wildfire periods and all other non-wildfire periods is summarized in **Figure S9** and **Table S13**.

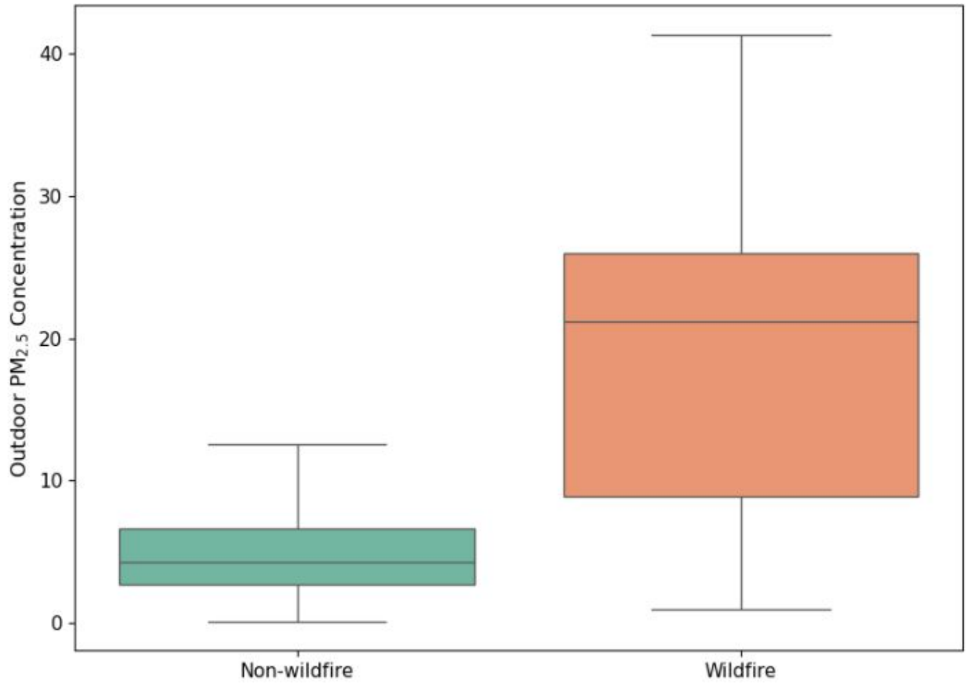

Figure S9. Comparison of outdoor $PM_{2.5}$_alt concentrations measured during wildfire and non-wildfire periods (Aug 22-24 and Sep 21-25, 2023)

Table S13. Summary of outdoor $PM_{2.5}$_alt concentrations during wildfire and non-wildfire periods

Period	Count	Concentration (μg/m^3)						
		Mean	SD	Min	25%	50%	75%	Max
Non-wildfire	42976	5.37	5.76	0.12	2.71	4.23	6.66	440.98
Wildfire	2304	18.45	9.31	0.95	8.90	21.15	26.00	41.32

For reference, **Figure S10** compares hourly average $PM_{2.5}$ concentrations from the outdoor PA in our study (located at Fuller Acres) that yielded a large amount of outdoor data throughout the study and that was closest to a regulatory ambient air quality monitoring station in Bakersfield (Bakersfield - California Ave, EPA AQS Site #60290014, utilizing a beta attenuation monitor, or BAM, ~15 km away). Data are shown separately for the entire study period for which data were available from this PA (April 30 – October 5, 2023, with 3784 hours of paired data available) and for the identified wildfire periods (August 22-24 and September 21-25, 2023, with 192 hours of paired data available). For the full data set, readings from the PA were moderately correlated with the nearest EPA BAM readings (R^2 = 0.65) and the PA modestly underestimated $PM_{2.5}$ concentrations on average (slope of 0.79). For data from just the wildfire periods, the correlation was stronger (R^2 = 0.91) and the PA somewhat overestimated $PM_{2.5}$ concentrations (slope of 1.28). Since the monitors are ~15 km apart, and the monitoring technologies are different, perfect correlation is not necessarily expected.

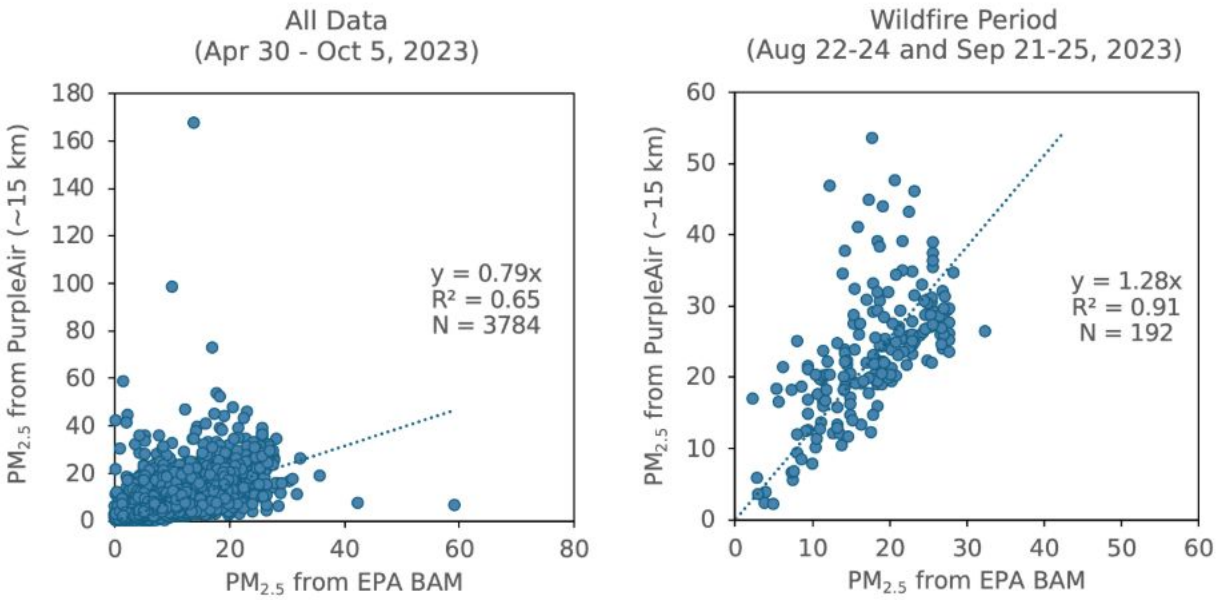

Figure S10. Hourly average outdoor $PM_{2.5}$ concentrations from the outdoor PA monitor in our study that was closest to a regulatory ambient air quality monitoring station in Bakersfield (Bakersfield - California Ave, EPA AQS Site #60290014, utilizing a beta attenuation monitor, or BAM, ~15 km away)

Table S14 summarizes the constrained I/O $PM_{2.5}$ ratios during these two periods when ECs were known or predicted to be operating (i.e., data comprising **Figure 4** in the main text), while **Table S15** and **Table S16** summarize these values for each home for non-wildfire and wildfire periods, respectively, comparing between times when ECs are known or predicted to be on vs. off.

Table S14. Summary of constrained I/O $PM_{2.5}$ ratios during non-wildfire and wildfire periods across all homes (supporting Figure 4 in the main text)

**Non-Wildfire periods**									
**Group**	**EC Run Mode[1]**	**Count**	**Mean**	**SD**	**Min**	**25%**	**50%**	**75%**	**Max**
PAC only	EC Off	64008	0.57	0.25	0.01	0.36	0.57	0.79	1.00
	EC On	10329	0.71	0.24	0.03	0.56	0.79	0.91	1.00
PAC+EC filter	EC Off	28500	0.58	0.25	0.00	0.37	0.61	0.79	1.00
	EC On	24409	0.64	0.18	0.02	0.53	0.64	0.76	1.00
**Wildfire periods**									
**Group**	**EC Run Mode[1]**	**Count**	**Mean**	**SD**	**Min**	**25%**	**50%**	**75%**	**Max**
PAC only	EC Off	9630	0.56	0.25	0.04	0.37	0.55	0.78	1.00
	EC On	1232	0.72	0.23	0.04	0.52	0.78	0.92	1.00
PAC+EC filter	EC Off	5483	0.59	0.25	0.05	0.36	0.62	0.80	1.00
	EC On	3102	0.69	0.14	0.28	0.58	0.67	0.79	1.00

[1]Includes measured and predicted EC runtime values

Table S15. Summary of constrained I/O $PM_{2.5}$ ratios during non-wildfire periods in each home (supporting Figure 4 in the main text)

**Non-Wildfire Periods**										
**Group**	**Home**	**EC Run Mode[1]**	**Count**	**Mean**	**SD**	**Min**	**25%**	**50%**	**75%**	**Max**
PAC only	FK07	EC Off	3815	0.55	0.22	0.13	0.37	0.52	0.73	1.00
		EC On	422	0.61	0.22	0.06	0.44	0.62	0.79	1.00
	FK13	EC Off	1577	0.59	0.27	0.06	0.39	0.64	0.82	1.00
		EC On	436	0.71	0.21	0.11	0.58	0.75	0.87	1.00
	FK16	EC Off	4689	0.35	0.20	0.02	0.19	0.31	0.45	1.00
		EC On	699	0.76	0.20	0.10	0.67	0.81	0.91	1.00
	FK19	EC Off	3536	0.57	0.22	0.01	0.41	0.53	0.72	1.00
		EC On	483	0.73	0.25	0.16	0.49	0.85	0.97	1.00
	FK25	EC Off	5082	0.73	0.18	0.04	0.61	0.76	0.87	1.00
		EC On	580	0.74	0.17	0.15	0.62	0.75	0.88	1.00
	FK27	EC Off	3454	0.68	0.19	0.06	0.54	0.67	0.83	1.00
		EC On	469	0.74	0.19	0.18	0.61	0.77	0.90	1.00
	FK28	EC Off	5604	0.61	0.27	0.02	0.36	0.69	0.86	1.00
		EC On	1317	0.74	0.19	0.09	0.61	0.80	0.88	1.00
	FK31	EC Off	2853	0.69	0.21	0.03	0.53	0.71	0.87	1.00
		EC On	396	0.75	0.20	0.10	0.62	0.79	0.91	1.00
	FK34	EC Off	2658	0.80	0.18	0.10	0.72	0.86	0.94	1.00
		EC On	550	0.80	0.18	0.16	0.69	0.85	0.94	1.00
	KE17	EC Off	5019	0.53	0.21	0.02	0.36	0.51	0.68	1.00
		EC On	170	0.67	0.27	0.05	0.46	0.74	0.89	1.00
	KE28	EC Off	4945	0.39	0.21	0.02	0.23	0.36	0.52	1.00
		EC On	790	0.79	0.19	0.04	0.66	0.85	0.94	1.00
	KE30	EC Off	3307	0.78	0.14	0.04	0.69	0.80	0.90	1.00

		EC On	398	0.77	0.15	0.43	0.65	0.79	0.90	1.00
	KE34	EC Off	4822	0.49	0.22	0.01	0.33	0.45	0.65	1.00
		EC On	1493	0.86	0.15	0.08	0.83	0.90	0.96	1.00
	KE35	EC Off	5741	0.60	0.24	0.03	0.39	0.59	0.81	1.00
		EC On	720	0.70	0.24	0.06	0.53	0.80	0.89	1.00
	KE38	EC Off	2306	0.36	0.21	0.03	0.20	0.32	0.46	1.00
		EC On	714	0.26	0.18	0.08	0.15	0.20	0.32	0.99
	KE40	EC Off	4600	0.51	0.25	0.01	0.31	0.47	0.71	1.00
		EC On	692	0.59	0.28	0.03	0.34	0.62	0.84	1.00
PAC+EC filter	FK06	EC Off	1094	0.75	0.18	0.10	0.64	0.78	0.89	1.00
		EC On	3368	0.78	0.18	0.07	0.67	0.84	0.92	1.00
	FK08	EC Off	3155	0.72	0.18	0.05	0.62	0.76	0.86	1.00
		EC On	3556	0.58	0.16	0.04	0.49	0.60	0.67	1.00
	FK10	EC Off	4208	0.49	0.21	0.07	0.32	0.45	0.64	1.00
		EC On	2389	0.77	0.15	0.04	0.69	0.79	0.89	1.00
	FK14	EC Off	3232	0.72	0.17	0.14	0.63	0.74	0.84	1.00
		EC On	955	0.67	0.17	0.08	0.56	0.70	0.79	1.00
	FK15	EC Off	1769	0.61	0.19	0.05	0.47	0.62	0.76	1.00
		EC On	5007	0.65	0.16	0.03	0.58	0.68	0.74	1.00
	FK26	EC Off	3414	0.69	0.20	0.09	0.57	0.72	0.85	1.00
		EC On	1225	0.59	0.16	0.08	0.51	0.61	0.69	1.00
	FK37	EC Off	2598	0.29	0.18	0.03	0.17	0.24	0.36	0.99
		EC On	1606	0.54	0.11	0.02	0.48	0.53	0.57	1.00
	FK38	EC Off	787	0.58	0.17	0.04	0.47	0.57	0.69	1.00
		EC On	2101	0.58	0.11	0.14	0.52	0.56	0.63	1.00
	FK39	EC Off	808	0.41	0.18	0.00	0.30	0.40	0.50	0.99
		EC On	87	0.50	0.19	0.10	0.38	0.48	0.62	0.97
	KE11	EC Off	1534	0.53	0.26	0.02	0.29	0.53	0.74	1.00
		EC On	381	0.75	0.16	0.13	0.62	0.78	0.88	1.00
	KE19	EC Off	2701	0.37	0.16	0.02	0.27	0.34	0.44	1.00
		EC On	1731	0.55	0.17	0.02	0.48	0.54	0.61	1.00
	KE21	EC Off	180	0.21	0.15	0.05	0.10	0.15	0.32	0.82
		EC On	232	0.32	0.20	0.05	0.14	0.35	0.44	0.98
	KE24	EC Off	2797	0.79	0.14	0.03	0.70	0.80	0.89	1.00
		EC On	1143	0.64	0.11	0.09	0.59	0.64	0.70	1.00
	KE25	EC Off	n/a	n/a	n/a	n/a	n/a	n/a	n/a	n/a
		EC On	577	0.64	0.17	0.04	0.53	0.61	0.78	1.00
	KE33	EC Off	223	0.14	0.09	0.05	0.08	0.11	0.16	0.65
		EC On	51	0.45	0.07	0.33	0.39	0.43	0.49	0.64

[1]Includes measured and predicted EC runtime values

Table S16. Summary of the constrained I/O $PM_{2.5}$ ratios during wildfire periods in each home (supporting Figure 4 in the main text)

**Wildfire periods**										
**Group**	**Home**	**EC Run Mode[1]**	**Count**	**Mean**	**SD**	**Min**	**25%**	**50%**	**75%**	**Max**
PAC only	FK07	EC Off	579	0.51	0.21	0.18	0.35	0.46	0.67	1.00
		EC On	69	0.59	0.21	0.23	0.44	0.55	0.76	0.99
	FK16	EC Off	746	0.40	0.18	0.10	0.30	0.38	0.46	0.99
		EC On	34	0.91	0.09	0.66	0.85	0.95	0.97	1.00
	FK19	EC Off	569	0.58	0.16	0.24	0.47	0.55	0.66	1.00
		EC On	38	0.67	0.21	0.30	0.51	0.64	0.89	1.00
	FK25	EC Off	726	0.84	0.10	0.48	0.79	0.86	0.91	1.00

		EC On	64	0.86	0.10	0.59	0.83	0.89	0.93	0.98
	FK27	EC Off	630	0.59	0.16	0.17	0.49	0.57	0.68	1.00
		EC On	60	0.63	0.17	0.35	0.48	0.63	0.77	0.98
	FK28	EC Off	782	0.64	0.26	0.18	0.39	0.71	0.88	1.00
		EC On	150	0.77	0.20	0.24	0.72	0.84	0.92	0.99
	FK31	EC Off	396	0.73	0.15	0.38	0.62	0.73	0.85	1.00
		EC On	38	0.82	0.13	0.48	0.75	0.82	0.93	1.00
	FK34	EC Off	407	0.82	0.20	0.38	0.76	0.92	0.96	1.00
		EC On	90	0.93	0.10	0.45	0.93	0.95	0.98	1.00
	KE17	EC Off	945	0.48	0.21	0.11	0.30	0.46	0.64	1.00
		EC On	10	0.85	0.15	0.60	0.76	0.89	0.96	1.00
	KE28	EC Off	803	0.24	0.17	0.04	0.11	0.19	0.30	0.99
		EC On	174	0.55	0.15	0.36	0.45	0.50	0.62	0.99
	KE30	EC Off	518	0.78	0.17	0.37	0.66	0.82	0.92	1.00
		EC On	92	0.83	0.14	0.36	0.76	0.86	0.93	1.00
	KE34	EC Off	723	0.51	0.15	0.21	0.41	0.51	0.58	1.00
		EC On	115	0.79	0.20	0.35	0.61	0.89	0.95	1.00
	KE35	EC Off	872	0.56	0.22	0.22	0.36	0.54	0.73	0.99
		EC On	116	0.85	0.13	0.37	0.78	0.89	0.94	1.00
	KE38	EC Off	249	0.28	0.18	0.07	0.12	0.23	0.42	0.99
		EC On	65	0.24	0.19	0.08	0.10	0.15	0.34	0.90
	KE40	EC Off	685	0.60	0.21	0.07	0.44	0.59	0.75	1.00
		EC On	117	0.65	0.20	0.04	0.49	0.62	0.83	1.00
PAC+EC filter	FK06	EC Off	331	0.75	0.10	0.48	0.68	0.73	0.81	1.00
		EC On	556	0.87	0.07	0.53	0.83	0.88	0.92	1.00
	FK08	EC Off	696	0.80	0.11	0.42	0.72	0.78	0.88	1.00
		EC On	271	0.65	0.07	0.42	0.62	0.64	0.68	0.97
	FK10	EC Off	692	0.42	0.18	0.17	0.29	0.34	0.51	0.99
		EC On	166	0.70	0.08	0.41	0.66	0.70	0.75	0.92
	FK14	EC Off	126	0.82	0.10	0.58	0.76	0.83	0.90	0.99
		EC On	n/a	n/a	n/a	n/a	n/a	n/a	n/a	n/a
	FK15	EC Off	353	0.63	0.14	0.37	0.51	0.61	0.72	1.00
		EC On	590	0.70	0.07	0.40	0.66	0.69	0.73	0.98
	FK26	EC Off	580	0.83	0.10	0.51	0.76	0.83	0.90	1.00
		EC On	67	0.71	0.11	0.51	0.64	0.70	0.76	1.00
	FK37	EC Off	622	0.29	0.16	0.05	0.20	0.28	0.35	0.97
		EC On	287	0.55	0.09	0.28	0.50	0.54	0.58	1.00
	FK38	EC Off	191	0.66	0.17	0.28	0.53	0.63	0.79	1.00
		EC On	370	0.54	0.08	0.34	0.48	0.52	0.56	0.97
	FK39	EC Off	587	0.49	0.17	0.12	0.35	0.46	0.58	1.00
		EC On	n/a	n/a	n/a	n/a	n/a	n/a	n/a	n/a
	KE11	EC Off	77	0.35	0.24	0.11	0.16	0.25	0.49	0.98
		EC On	43	0.90	0.10	0.42	0.86	0.92	0.95	1.00
	KE19	EC Off	754	0.39	0.13	0.09	0.30	0.37	0.46	0.96
		EC On	142	0.63	0.14	0.43	0.56	0.59	0.64	1.00
	KE24	EC Off	474	0.85	0.08	0.60	0.78	0.85	0.91	1.00
		EC On	13	0.64	0.11	0.53	0.59	0.61	0.65	0.94
	KE25	EC Off	n/a	n/a	n/a	n/a	n/a	n/a	n/a	n/a
		EC On	597	0.68	0.13	0.38	0.58	0.66	0.75	1.00

[1]Includes measured and predicted EC runtime values

Sensitivity analyses: Constrained I/O threshold and outdoor-to-indoor lag

To evaluate the sensitivity and appropriateness of the assumed constrained I/O threshold of I/O ≤ 1 (without any outdoor-to-indoor lag), we first calculated correlation coefficients (Pearson's R) between concurrent indoor and outdoor $PM_{2.5}$ concentrations for each home as a function of varying constrained I/O thresholds from 0 to 5 in increments of 0.5 to ensure that we observe what we would expect to observe. **Figure S11** plots the mean (+/- SD) of correlation coefficients across homes at these different thresholds. As expected, the highest correlation was with an I/O threshold of 1 and decreased with increasing constrained I/O threshold, suggesting that as the threshold relaxes, more indoor sources are included, and correlations between indoor and outdoor are weaker.

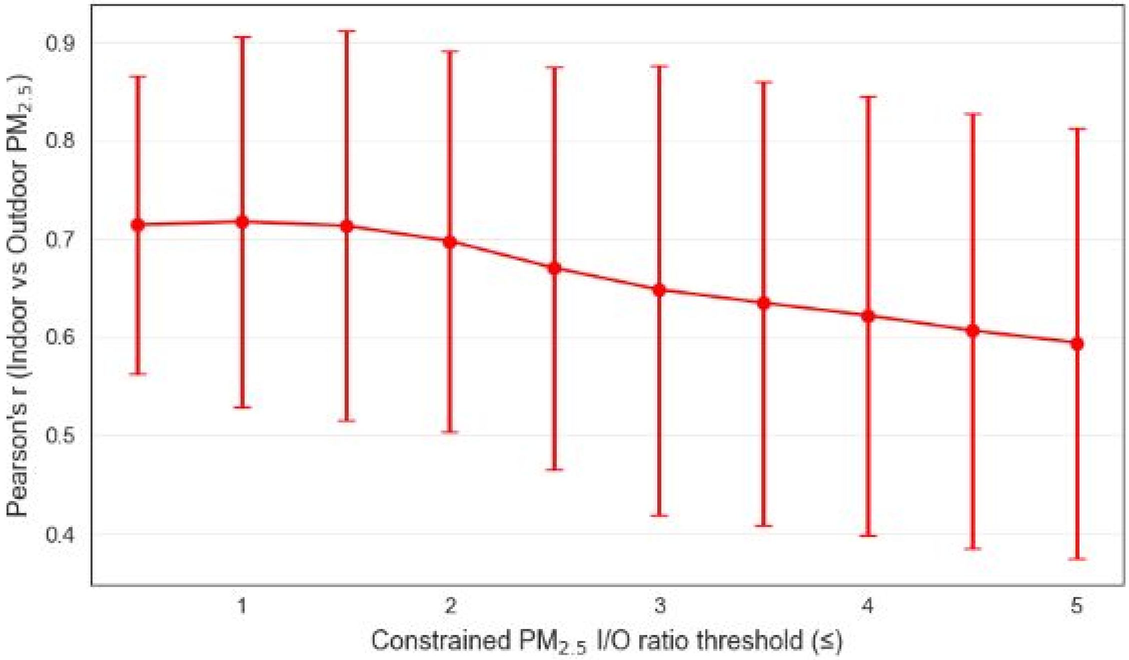

Figure S11. Correlations between indoor and outdoor $PM_{2.5}$ concentrations as a function of constrained $PM_{2.5}$ I/O ratio thresholds (0.5 - 5, step = 0.5)

Figure S12 presents more granular results within a narrower I/O threshold range of 0.5 to 1.5 in increments of 0.1. There does not appear to be much difference across much of this range in this correlation metric. To 3 decimal places, correlation coefficients were 0.713, 0.717, and 0.716 for thresholds of 0.9, 1.0, and 1.1, respectively. **Figure S13** provides similar correlations but separates wildfire periods from non-wildfire periods. For the wildfire periods, there is a slightly stronger correlation at I/O threshold of 1.0 than 1.1, and an increasingly stronger correlation at lower I/O thresholds. However, reducing the threshold much lower than 1 is inadvisable because it would exclude many I/O values near 1 that are likely legitimate signals of ambient $PM_{2.5}$

infiltration, especially with ECs operating. The combination of these results suggests that the assumed I/O threshold of 1.0 is reasonable.

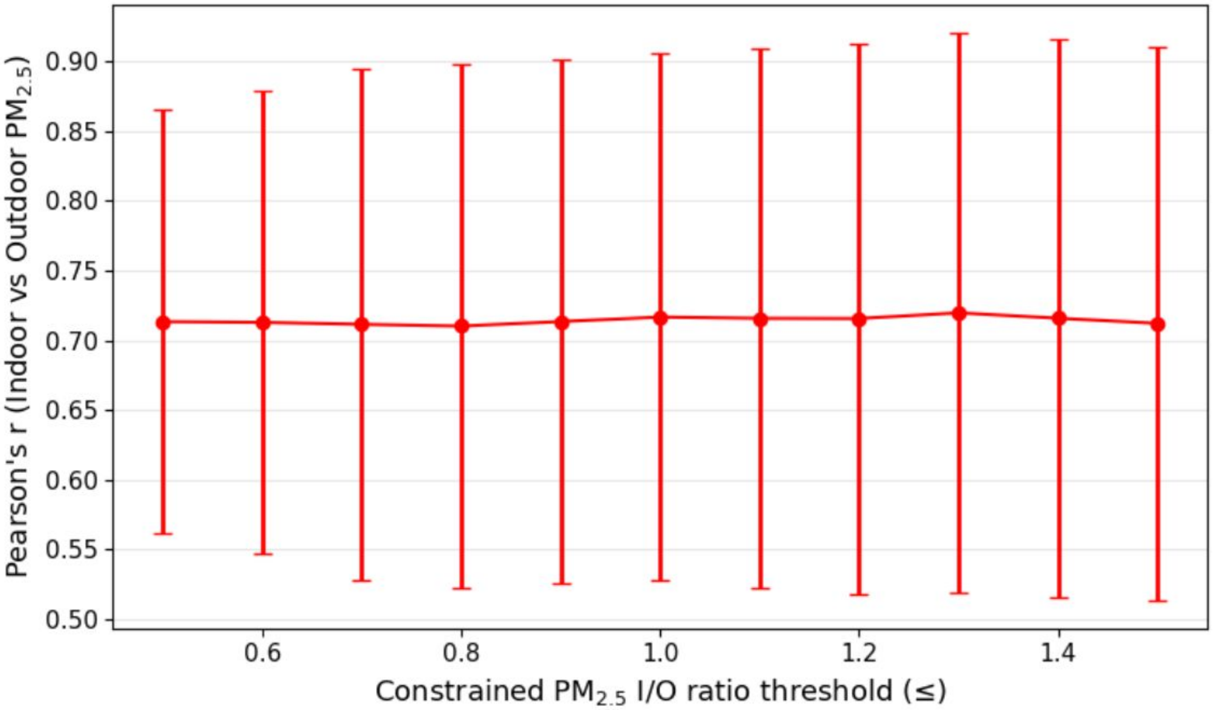

Figure S12. Correlations between concurrent indoor and outdoor $PM_{2.5}$ concentrations as a function of constrained $PM_{2.5}$ I/O ratio thresholds (0.5 - 1.5, step = 0.1)

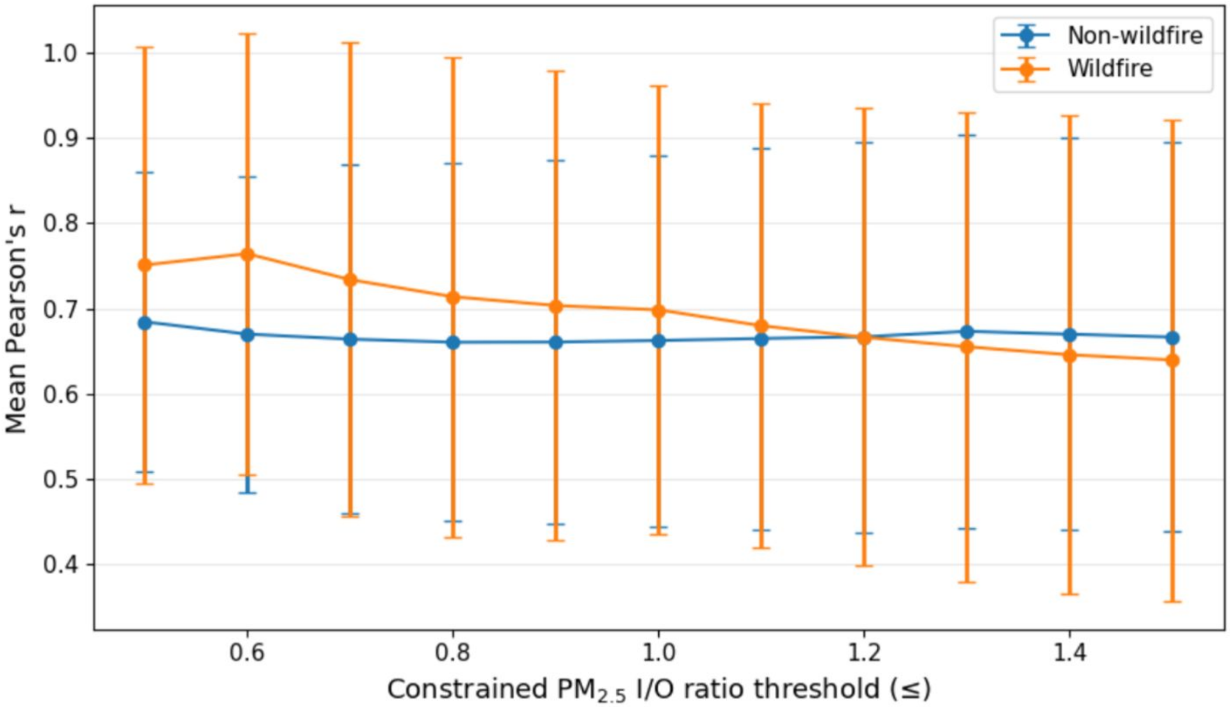

Figure S13. Correlations between concurrent indoor and outdoor $PM_{2.5}$ concentrations during wildfire periods and non-wildfire periods as a function of constrained $PM_{2.5}$ I/O ratio thresholds (0.5 - 1.5, step = 0.1)

Next, we recreate Figures 3 and 4 from the main text with I/O thresholds of 0.9, 1.0, and 1.1 to test the sensitivity of results to these assumptions (**Figures S14** and **S15**). The results are only modestly sensitive to I/O threshold, with similar median values for all three thresholds. The lowest median values are for threshold of 0.9 and the highest median values were for 1.1. Given that EC operation can often lead to I/O near 1, we do not think it is appropriate to use 0.9 as a threshold, and the lower median compared to threshold of 1.0 suggests that indeed values between 0.9 and 1.0 are getting excluded when perhaps they should not be. Conversely, a threshold of 1.1 may overestimate impacts by including times of modest indoor sources. Therefore, the combination of correlation coefficients and sensitivity analysis across all data and also the smaller subset of wildfire period data suggests that keeping I/O threshold of 1.0 is reasonable as our primary endpoint.

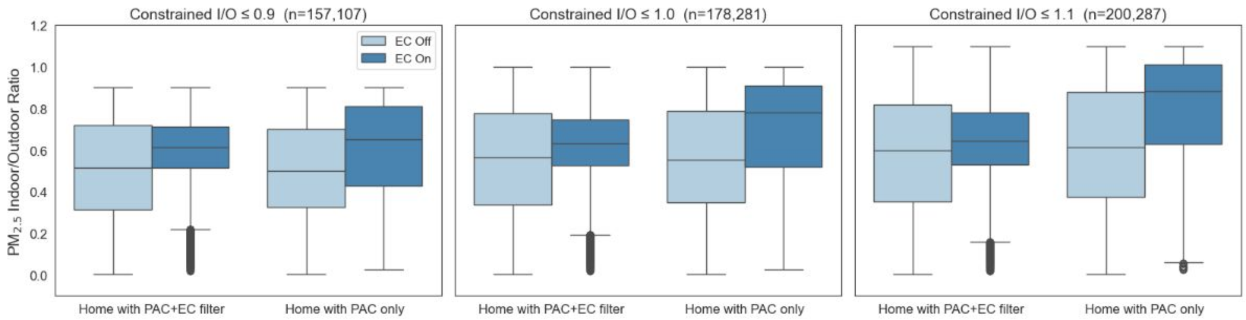

Figure S14. Comparison of constrained $PM_{2.5}$ I/O ratios between homes with PAC + EC filter homes with PAC only, conditionally comparing when ECs were measured or predicted to be on to when they were measured or predicted to be off, using all available post-intervention data, constrained to I/O ≤ 0.9, 1, and 1.1 to test sensitivity

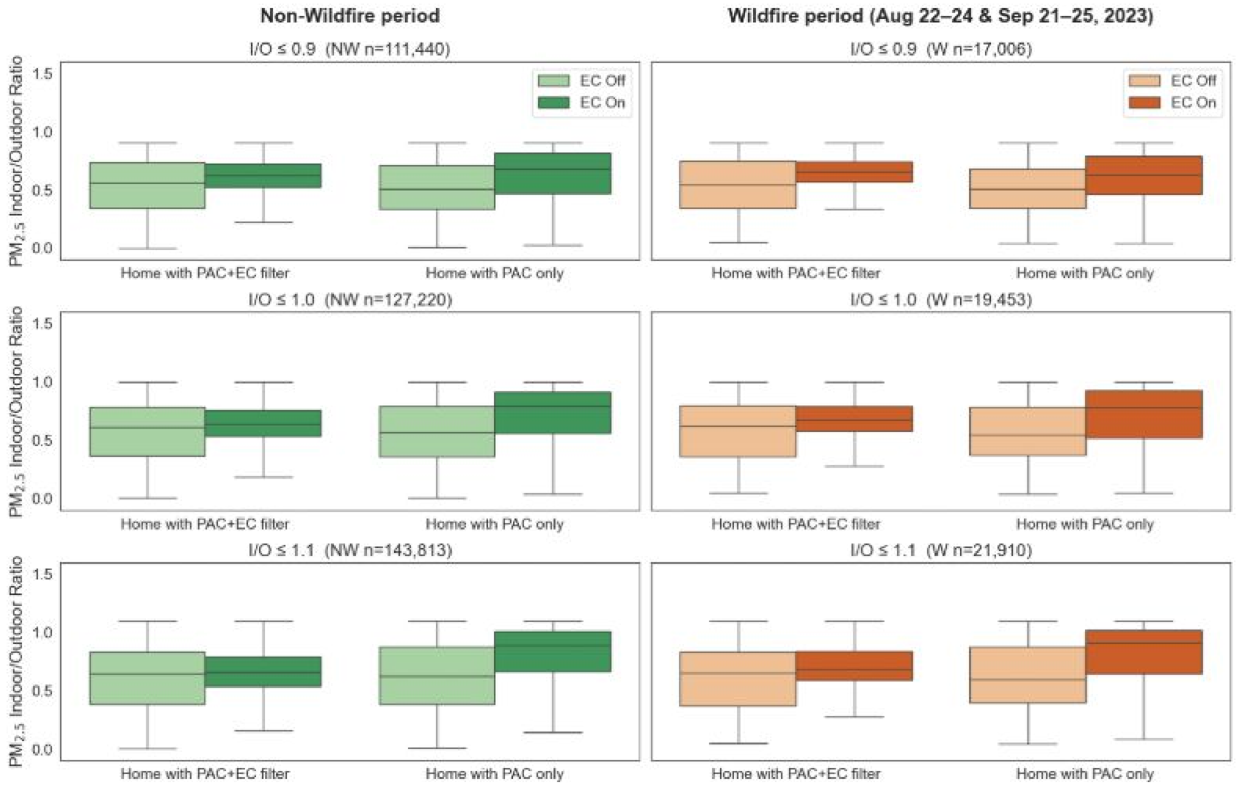

Figure S15. Comparison of constrained I/O $PM_{2.5}$ ratios in all homes during (a) non-wildfire periods and (b) wildfire event periods, comparing PAC + EC filter homes vs. PAC only homes, conditionally comparing when ECs were measured or predicted to be on to when they were measured or predicted to be off, using all available post-intervention data, constrained to I/O ≤ 0.9, 1, and 1.1 to test sensitivity

Next, we evaluate the sensitivity and appropriateness of assuming no time lag between outdoor and indoor $PM_{2.5}$ concentrations by testing different lag periods. First, **Figure S16** shows correlation coefficients (Pearson's R) calculated between indoor and outdoor $PM_{2.5}$ concentrations for each home (showing mean ± SD across homes) as a function of time delay (from 0-120 minutes in increments of 10 minutes) for two data sets: one with all data (which includes periods with indoor sources) and one without indoor sources (i.e., the remaining indoor concentrations after running the F_{inf} algorithm). Correlations are weak using all data (mean R < 0.2), as expected given the high prevalence of indoor sources in this data set, while correlations are relatively high (mean R ~0.7 or higher) for all lag periods when applied to the data flagged as likely being free of indoor sources. Additionally, the highest correlation coefficient with ambient-infiltrated (F_{inf} filtered) data was with a 10-minute lag (R ~0.8) followed by a 20-minute lag. The rest of the lag comparisons yield similar correlation coefficients near 0.7. These results suggest that a 10-minute lag may be the most appropriate for the F_{inf} filtered indoor concentration data. However, before making that decision, **Figure S17** further explores correlations between indoor and outdoor $PM_{2.5}$ concentrations at different lag periods when

applied to the constrained I/O ratio data (including thresholds of 0.9, 1.0, and 1.1) rather than the F_{inf} filtered data, since that is our primary data source throughout the main analysis. These results show the highest correlation between indoor and outdoor $PM_{2.5}$ concentrations when constrained below 0.9, 1.0, and 1.1 are all at a lag of 0 minutes, and that there were no differences in correlations at the different constrained ratio thresholds.

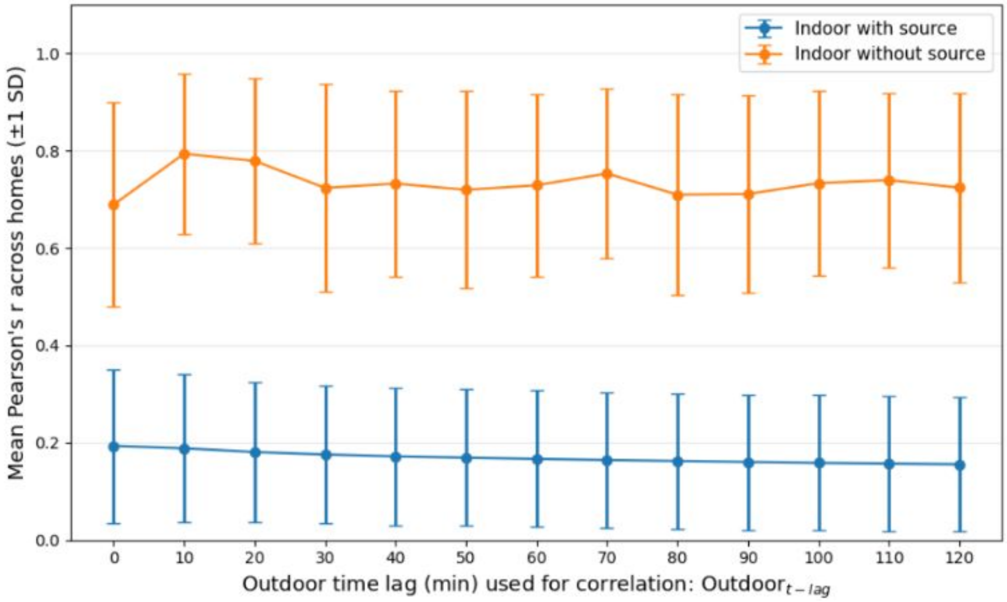

Figure S16. Correlations between indoor and outdoor $PM_{2.5}$ concentration using (a) all data available and (b) the remaining data after applying the F_{inf} algorithm as a function of outdoor-to-indoor concentration time lag (0-120 min, step = 10 min)

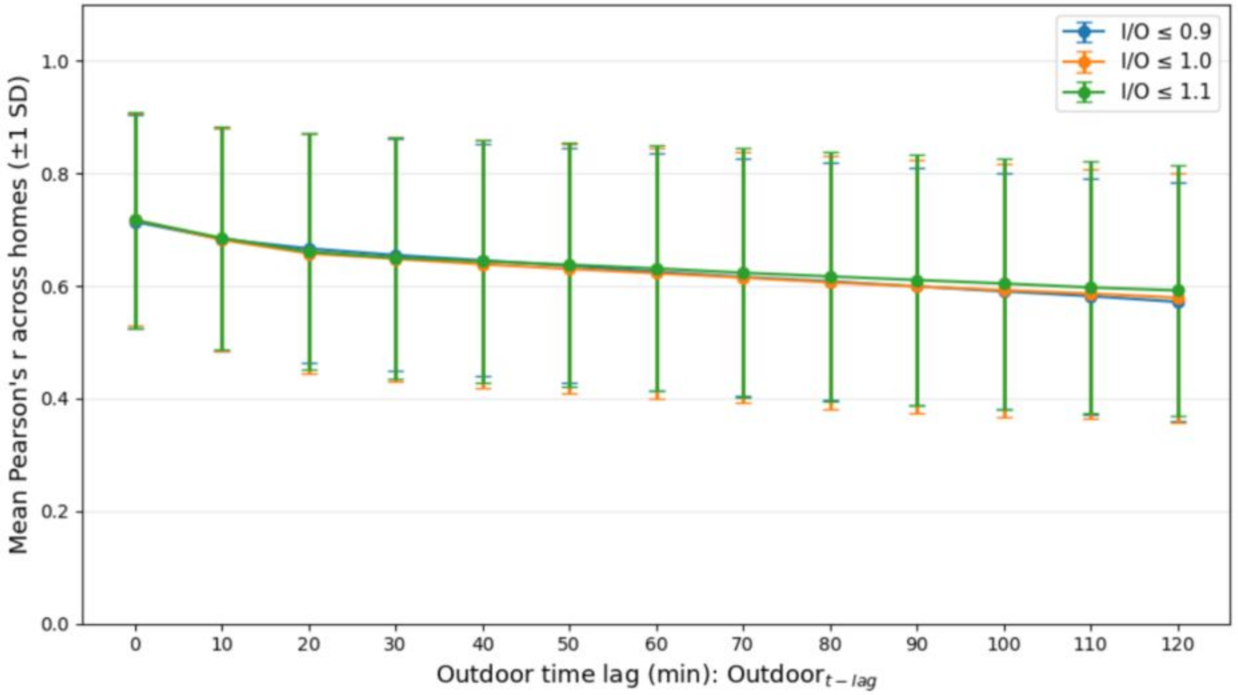

Figure S17. Correlations between indoor and outdoor $PM_{2.5}$ concentrations using all data available constrained to I/O ≤ 0.9, 1, and 1.1, as a function of outdoor-to-indoor concentration time lag (0-120 min, step = 10 min)

Before exploring the sensitivity of results to the different lag assumptions, it is worth emphasize that these comparisons also illustrate important aspect: the average correlation coefficients between indoor and outdoor $PM_{2.5}$ concentrations when using constrained I/O ratios are very similar to correlations between indoor and outdoor $PM_{2.5}$ concentrations when applied only to data that have been filtered to remove indoor source effects, each with a mean R of ~0.7. This suggests that the constrained I/O ratio approach is likely doing a reasonable job of isolating indoor PM of predominantly outdoor origin, as desired, which supports using this metric as our primary endpoint, especially given how much more data it provides.

Next, to test the sensitivity of the intervention group comparison results to different assumptions for indoor-outdoor lag periods, **Figure S18** recreates **Figure 3** to compare constrained I/O ratios ≤ 1 and F_{inf} for lag periods from 0 to 40 minutes in intervals of 10 minutes. The results are not very sensitive to different assumptions for lag periods in this range. Therefore, between observing a lack of sensitivity to lag assumptions and the strongest correlations at a lag of 0 minutes shown above, we have confidence that our primary assumption of no lag is reasonable.

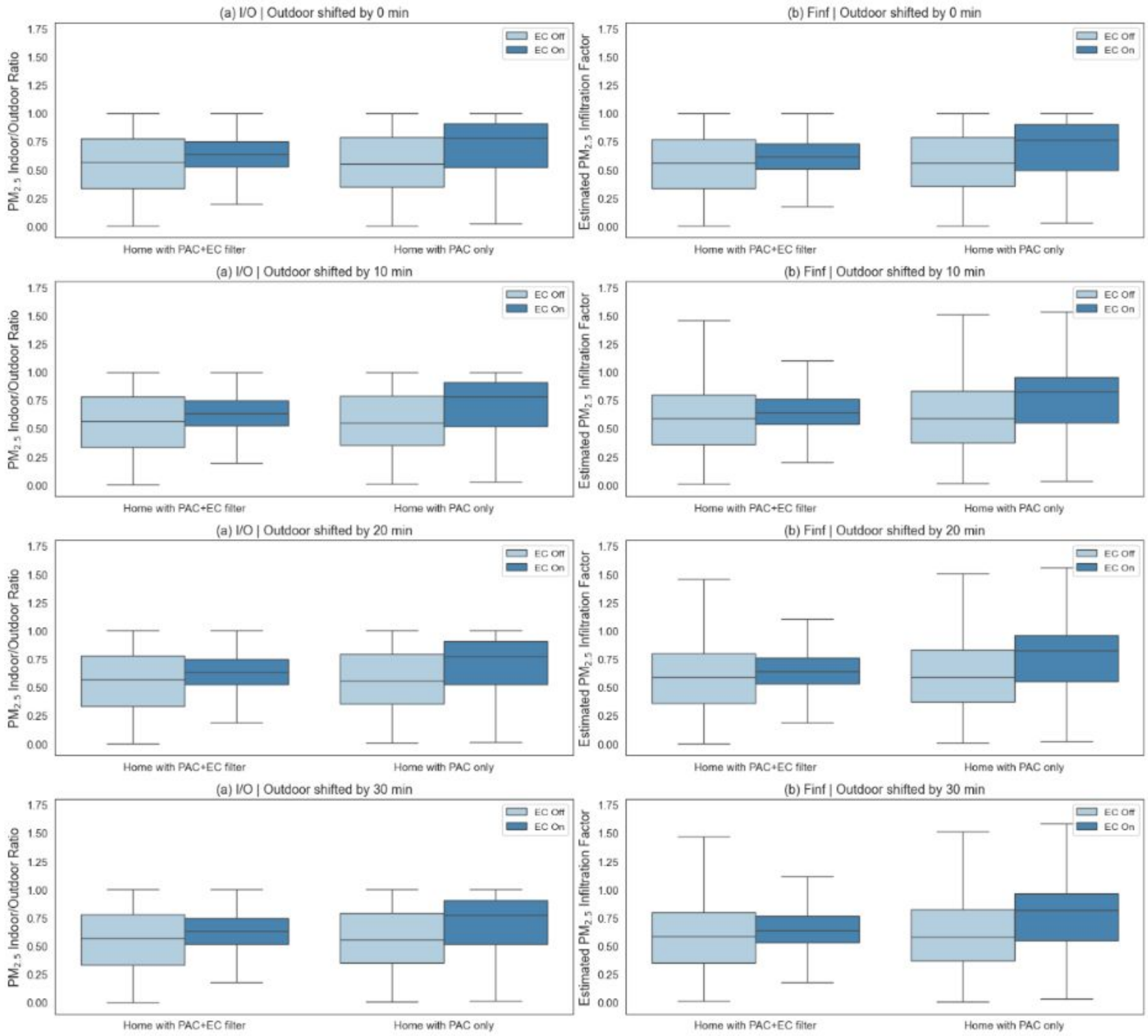

Figure S18. Comparison of (a) $PM_{2.5}$ I/O ratios constrained to I/O ≤ 1 (i.e., excluding indoor sources) and (b) estimated $PM_{2.5}$ infiltration factor between homes with PAC + EC filter homes with PAC only, conditionally comparing when ECs were known or predicted to be on to when they were known or predicted to be off, using all available post-intervention data, with time lags between indoor and outdoor concentrations of 0, 10, 20, and 30 minutes to test sensitivity

New vs. used filters

Table S17 summarizes in tabular form the data in **Figure 5** of the main text showing constrained I/O $PM_{2.5}$ ratios in homes with PAC and EC filters compared during the first three weeks of DIY filter installation to a three-week period after 50 days of installation. **Table S18** expands these data to show values for each home.

Table S17. Summary of constrained I/O $PM_{2.5}$ ratios with new and used EC filters across all homes (supporting Figure 5 in the main text)

Period	EC Run Mode	Home #	Count	Mean	SD	Min	25%	50%	75%	Max
Day 1–21 of Post-Intervention Period	EC Off	15	15375	0.54	0.24	0.00	0.36	0.55	0.73	1.00
	EC On	16	17039	0.59	0.17	0.04	0.49	0.59	0.69	1.00
Day 51–71 of Post-Intervention Period	EC Off	12	13857	0.55	0.26	0.02	0.33	0.55	0.78	1.00
	EC On	11	3981	0.65	0.18	0.02	0.56	0.67	0.76	1.00

Table S18. Summary of constrained I/O $PM_{2.5}$ ratios with new and used EC filters in each home (supporting Figure 5 in the main text)

Period	EC Run Mode	Home	Count	Mean	SD	Min	25%	50%	75%	Max
Day 1–21 post-intervention	EC Off	FK06	261	0.69	0.18	0.28	0.56	0.66	0.83	1.00
		FK08	926	0.59	0.19	0.12	0.43	0.59	0.73	1.00
		FK10	1414	0.45	0.20	0.07	0.29	0.42	0.58	1.00
		FK14	1437	0.66	0.18	0.14	0.55	0.66	0.78	1.00
		FK15	402	0.54	0.22	0.13	0.38	0.52	0.70	1.00
		FK26	1304	0.60	0.21	0.12	0.43	0.61	0.77	1.00
		FK37	1416	0.58	0.19	0.07	0.45	0.57	0.72	1.00
		FK38	674	0.56	0.16	0.04	0.46	0.53	0.65	1.00
		FK39	2392	0.45	0.16	0.00	0.34	0.44	0.54	1.00
		KE11	1151	0.58	0.25	0.02	0.37	0.60	0.79	1.00
		KE21	52	0.38	0.13	0.22	0.31	0.35	0.40	0.82
		KE24	972	0.79	0.13	0.13	0.71	0.80	0.90	1.00
		KE25	631	0.67	0.13	0.15	0.57	0.65	0.73	1.00
		KE37	1201	0.18	0.13	0.03	0.10	0.16	0.23	1.00
		KE39	1142	0.59	0.30	0.06	0.29	0.66	0.87	1.00
	EC On	FK06	1487	0.69	0.18	0.07	0.57	0.70	0.83	1.00
		FK08	1886	0.50	0.16	0.04	0.39	0.51	0.60	1.00
		FK10	1182	0.73	0.17	0.17	0.63	0.75	0.86	1.00
		FK14	572	0.62	0.18	0.08	0.49	0.64	0.74	1.00
		FK15	2169	0.57	0.18	0.05	0.45	0.57	0.69	1.00
		FK26	807	0.55	0.16	0.08	0.45	0.55	0.64	1.00
		FK37	1062	0.51	0.18	0.05	0.42	0.53	0.61	0.99
		FK38	1848	0.58	0.10	0.14	0.51	0.56	0.62	1.00
		FK39	274	0.48	0.14	0.10	0.41	0.46	0.53	0.97
		KE11	312	0.73	0.15	0.13	0.61	0.75	0.84	1.00
		KE21	89	0.47	0.16	0.24	0.39	0.42	0.48	0.98
		KE24	758	0.62	0.10	0.09	0.58	0.62	0.67	1.00
		KE25	1296	0.66	0.15	0.04	0.55	0.63	0.75	1.00
		KE33	3	0.41	0.02	0.39	0.40	0.41	0.42	0.44
		KE37	1777	0.50	0.14	0.10	0.42	0.51	0.59	0.99

		KE39	1517	0.63	0.12	0.08	0.56	0.63	0.70	0.99
Day 51–71 post-intervention	EC Off	FK06	609	0.75	0.13	0.18	0.67	0.74	0.84	1.00
		FK08	1205	0.79	0.13	0.10	0.72	0.79	0.89	1.00
		FK10	1339	0.52	0.21	0.08	0.32	0.49	0.70	1.00
		FK14	276	0.80	0.08	0.58	0.74	0.79	0.86	1.00
		FK15	828	0.58	0.17	0.05	0.46	0.57	0.70	1.00
		FK26	1161	0.78	0.16	0.09	0.68	0.80	0.90	1.00
		FK37	851	0.36	0.17	0.03	0.25	0.32	0.42	0.99
		KE19	2014	0.37	0.12	0.02	0.28	0.36	0.44	0.98
		KE24	970	0.79	0.13	0.07	0.72	0.79	0.88	1.00
		KE36	1560	0.58	0.19	0.08	0.43	0.55	0.71	1.00
		KE37	1353	0.18	0.17	0.04	0.08	0.12	0.19	0.95
		KE39	1691	0.57	0.26	0.04	0.34	0.52	0.84	1.00
	EC On	FK06	616	0.86	0.10	0.08	0.83	0.87	0.91	1.00
		FK08	393	0.68	0.10	0.38	0.62	0.66	0.71	0.98
		FK10	296	0.74	0.10	0.41	0.68	0.74	0.79	0.99
		FK14	12	0.84	0.04	0.78	0.82	0.84	0.86	0.90
		FK15	975	0.69	0.09	0.06	0.65	0.69	0.72	1.00
		FK37	270	0.54	0.12	0.25	0.46	0.53	0.58	0.94
		KE19	500	0.53	0.12	0.06	0.50	0.56	0.60	0.92
		KE24	51	0.65	0.10	0.27	0.62	0.65	0.70	0.95
		KE36	261	0.58	0.21	0.21	0.41	0.54	0.74	1.00
		KE37	297	0.33	0.11	0.02	0.24	0.31	0.41	0.87
		KE39	310	0.69	0.11	0.34	0.64	0.70	0.75	0.99

Pre/post-intervention comparisons

Figure S19 compares constrained I/O $PM_{2.5}$ ratios before and after interventions across all homes with data available (i.e., aggregate data from **Figure 6** in the main text), comparing homes with PAC only to those with both PAC and DIY EC filters, only when ECs were either known or predicted to be operating. In PAC only homes, the median constrained I/O $PM_{2.5}$ ratio slightly decreased from 0.82 pre-intervention to 0.81 post-intervention (-1%; d = 0.31), while in homes with PAC + EC filters, the median constrained I/O $PM_{2.5}$ ratio decreased from 0.86 pre-intervention to 0.66 post-intervention (-23%; d = 0.74). **Table S19** shows the underlying distributions in **Figure S19** while **Table S20** expands these data to show values for each home (supporting **Figure 6** in the main text).

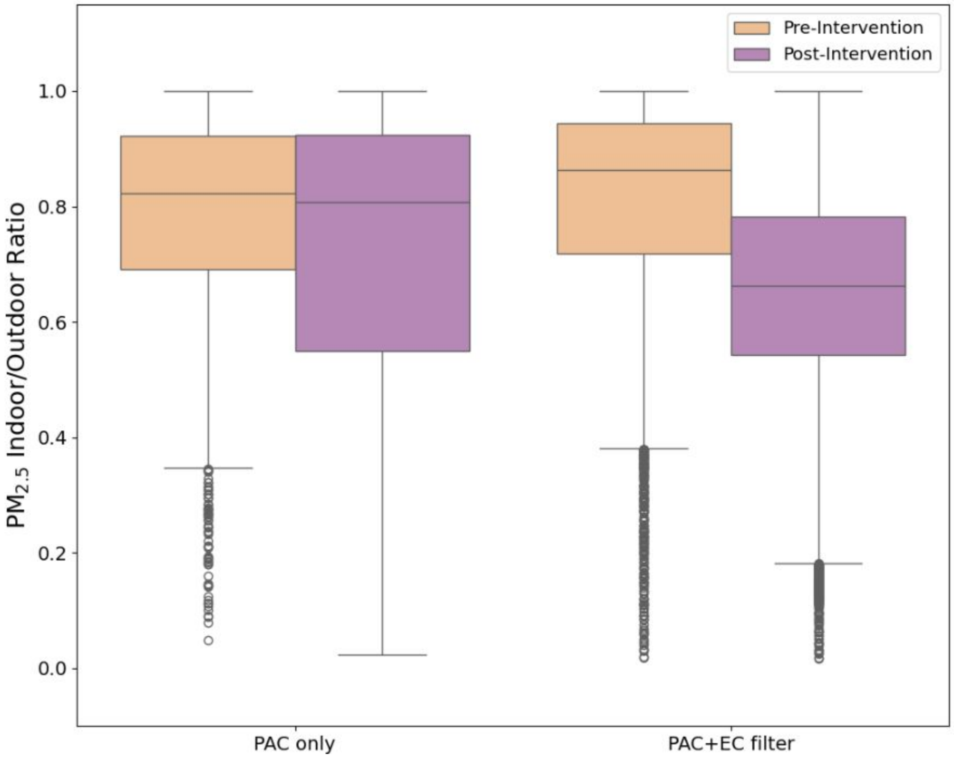

Figure S19. Comparison of constrained I/O $PM_{2.5}$ ratios between pre- and post-intervention periods with ECs known or predicted to be operating (aggregate data from Figure 6)

Table S19. Summary of constrained I/O $PM_{2.5}$ ratios between pre- and post-intervention periods with ECs known or predicted to be operating (Figure S19)

Group	Period	Count	Mean	SD	Min	25%	50%	75%	Max
PAC only	Post-Intervention	7567	0.72	0.24	0.02	0.55	0.81	0.92	1.00
	Pre-Intervention	2992	0.79	0.17	0.05	0.69	0.82	0.92	1.00
PAC+EC filter	Post-Intervention	23009	0.66	0.19	0.02	0.54	0.66	0.78	1.00
	Pre-Intervention	3515	0.80	0.20	0.02	0.72	0.86	0.94	1.00

Table S20. Summary of constrained I/O $PM_{2.5}$ ratios between pre- and post-intervention periods with ECs known or predicted to be operating in each home (supporting Figure 6 in the main text)

Group	Home	Pre/post-Intervention	Count	Mean	SD	25%	50%	75%	Min	Max	Median Diff. (%)
PAC only	FK07	Pre	565	0.80	0.14	0.71	0.82	0.90	0.26	1.00	-26%
		Post	491	0.61	0.22	0.44	0.61	0.79	0.06	1.00	
	FK12	Pre	464	0.80	0.15	0.73	0.84	0.92	0.18	1.00	-51%
		Post	1215	0.51	0.30	0.25	0.41	0.85	0.02	1.00	
	FK16	Pre	363	0.80	0.19	0.69	0.85	0.94	0.08	1.00	-5%
		Post	733	0.77	0.20	0.68	0.81	0.92	0.10	1.00	
	FK19	Pre	452	0.75	0.17	0.64	0.78	0.89	0.16	1.00	8%
		Post	521	0.73	0.25	0.50	0.84	0.97	0.16	1.00	
	FK27	Pre	263	0.81	0.17	0.73	0.84	0.94	0.09	1.00	-11%
		Post	529	0.73	0.19	0.59	0.75	0.89	0.18	1.00	
	KE17	Pre	133	0.84	0.21	0.79	0.93	0.97	0.11	1.00	-19%
		Post	180	0.68	0.26	0.47	0.75	0.89	0.05	1.00	
	KE28	Pre	235	0.69	0.19	0.55	0.68	0.87	0.19	1.00	16%
		Post	964	0.74	0.21	0.58	0.79	0.93	0.04	1.00	
	KE30	Pre	339	0.78	0.17	0.69	0.81	0.91	0.14	1.00	0%
		Post	490	0.78	0.15	0.67	0.81	0.91	0.36	1.00	
	KE34	Pre	115	0.81	0.20	0.74	0.87	0.94	0.05	1.00	3%
		Post	1608	0.85	0.16	0.83	0.90	0.96	0.08	1.00	
	KE35	Pre	63	0.86	0.11	0.78	0.87	0.96	0.53	1.00	-7%
		Post	836	0.72	0.23	0.59	0.81	0.90	0.06	1.00	
PAC+EC filter	FK06	Pre	289	0.87	0.14	0.82	0.92	0.97	0.23	1.00	-8%
		Post	3924	0.80	0.17	0.70	0.85	0.92	0.07	1.00	
	FK08	Pre	176	0.75	0.28	0.66	0.86	0.95	0.03	1.00	-29%
		Post	3827	0.58	0.15	0.50	0.61	0.67	0.04	1.00	
	FK10	Pre	437	0.78	0.16	0.70	0.81	0.90	0.15	1.00	-4%
		Post	2555	0.77	0.15	0.69	0.78	0.88	0.04	1.00	
	FK14	Pre	339	0.79	0.17	0.72	0.83	0.91	0.16	1.00	-16%
		Post	955	0.67	0.17	0.56	0.70	0.79	0.08	1.00	
	FK15	Pre	280	0.73	0.23	0.62	0.79	0.92	0.02	1.00	-14%
		Post	5597	0.65	0.15	0.59	0.68	0.74	0.03	1.00	
	KE11	Pre	322	0.71	0.23	0.53	0.75	0.91	0.11	1.00	5%
		Post	424	0.76	0.16	0.63	0.79	0.90	0.13	1.00	
	KE19	Pre	574	0.81	0.22	0.75	0.90	0.96	0.02	1.00	-39%
		Post	1873	0.56	0.17	0.49	0.55	0.61	0.02	1.00	
	KE24	Pre	10	0.65	0.19	0.52	0.67	0.76	0.29	0.93	-6%
		Post	1156	0.64	0.11	0.59	0.63	0.70	0.09	1.00	
	KE33	Pre	182	0.74	0.26	0.55	0.87	0.94	0.07	1.00	-51%
		Post	51	0.45	0.07	0.39	0.43	0.49	0.33	0.64	
	KE36	Pre	120	0.85	0.17	0.80	0.89	0.96	0.05	1.00	-30%
		Post	476	0.65	0.24	0.43	0.62	0.90	0.04	1.00	
	KE37	Pre	786	0.85	0.14	0.80	0.88	0.94	0.04	1.00	-43%
		Post	2171	0.48	0.15	0.39	0.50	0.58	0.02	0.99	

PAC usage and impact

Among the 44 homes in the merged dataset, 40 homes had PAC power draw measurements successfully logged by PLLs and 35 homes had overlapping time periods in which data from PurpleAir monitors and PLLs were available (another 5 homes had PLL data available but the data were not included in the merged dataset because their timestamps of available data did not overlap). The power draw data from each PAC were visually inspected to generate bounds of PAC fan speed settings for each type of PAC (i.e., low, medium, high, or off). **Figure S20** summarizes the percentage of time that the PAC in each home operated on each fan speed setting. On average, PACs were operated on low, medium, and high fan speed settings for 43%, 24%, and 12% of the time, respectively, and were not operated for the remaining 21% of the time. Homes with PAC only and homes with PAC + EC filters had similar average percentage of PAC run time on low, medium, and high fan speed settings: 41%, 25% and 15% for PAC only homes, and 45%, 23% and 8%, respectively. PACs were off 19% of the time in PAC only homes and 24% of the time in PAC + EC filter homes. PAC operation also varied by home, with some homes keeping their PACs off >90% of the time, some homes operating on high >75% of the time, although the majority operated them on low most often.

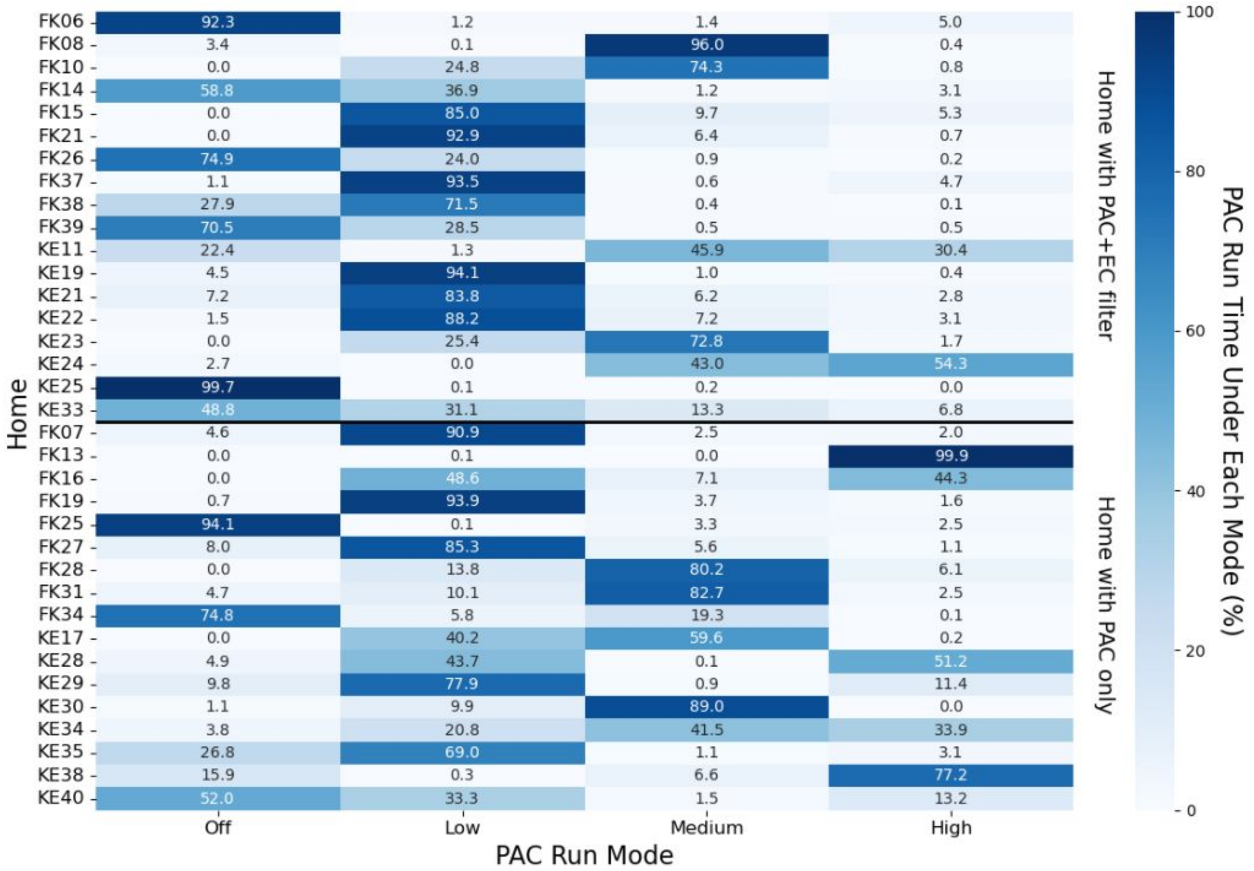

Figure S20. Percentage of PAC run time under each fan speed setting for each home

Figure S21 shows a binary indicator of daily average PAC runtime in each home, as well as the daily average across all homes, during the post-intervention period. While there is variability in daily PAC runtime in individual homes, the average daily runtime across all homes was maintained above 60% for every day of the post-intervention period, albeit with a gradual decline from an average of >80% of the time in the first few weeks to only <70% of the time in the last few weeks.

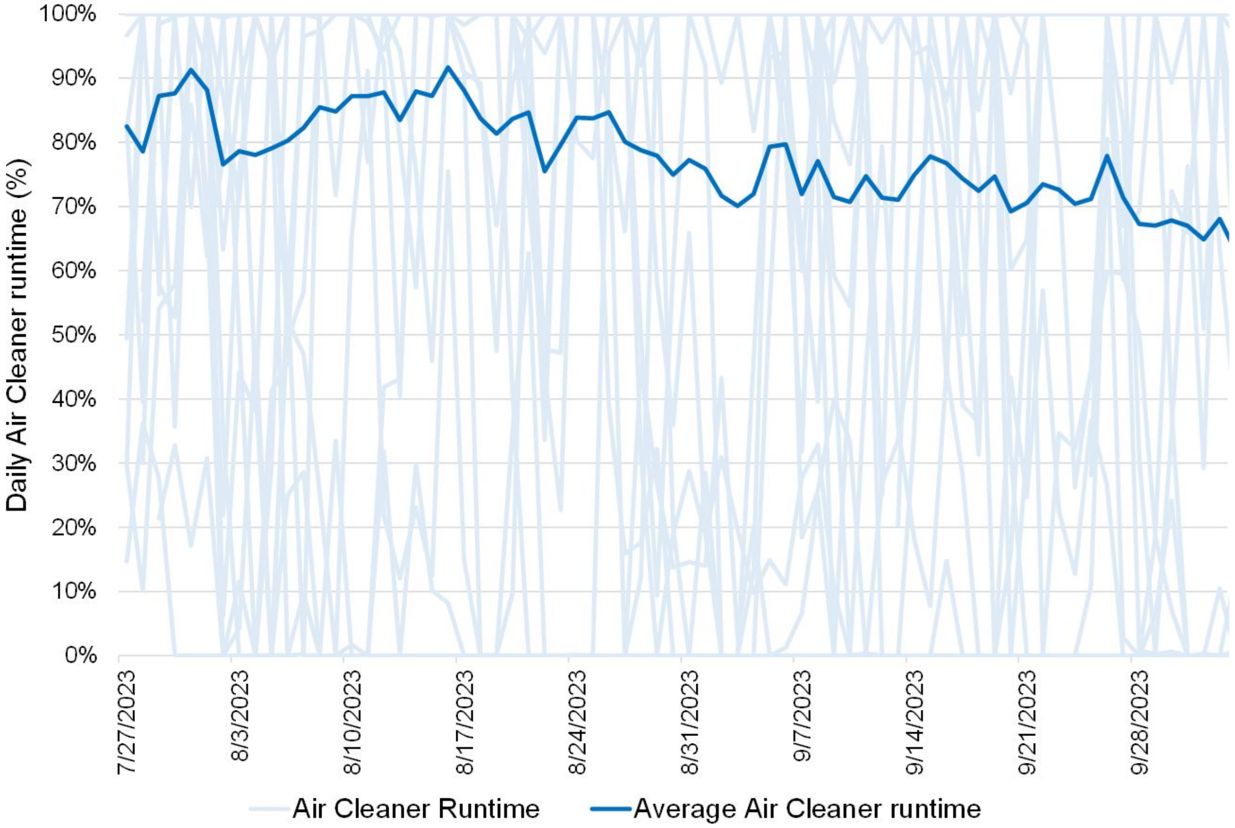

Figure S21. Daily average binary (on/off) portable air cleaner (PAC) runtime across all homes (individual and average of homes)

As another indicator of PAC usage and impact, the amount of clean air delivered in-situ by the PAC (i.e., the in-situ CADR) was calculated at each time step by multiplying the PLL-measured power draw by the manufacturer-reported energy efficiency rating (CADR/Watt) value for the corresponding PAC model. This value was then divided by the maximum CADR for each model PAC to generate an indicator of the percentage of maximum particle-free air (using smoke-size CADR) that was delivered in-situ at each time step. **Figure S**22 shows the average percentage of maximum CADR delivered in each home during the intervention period. Only one home operated their PAC on high fan speed settings often enough to achieve >80% of maximum

CADR; 6 homes operated between 20–80% of maximum CADR; and 29 homes operated <20% of their maximum CADR. On average, participants operated their PACs at around 16% of their maximum CADR, and the average (and median) in-situ CADR across homes was ~57 m^3/h (~27 m^3/h).

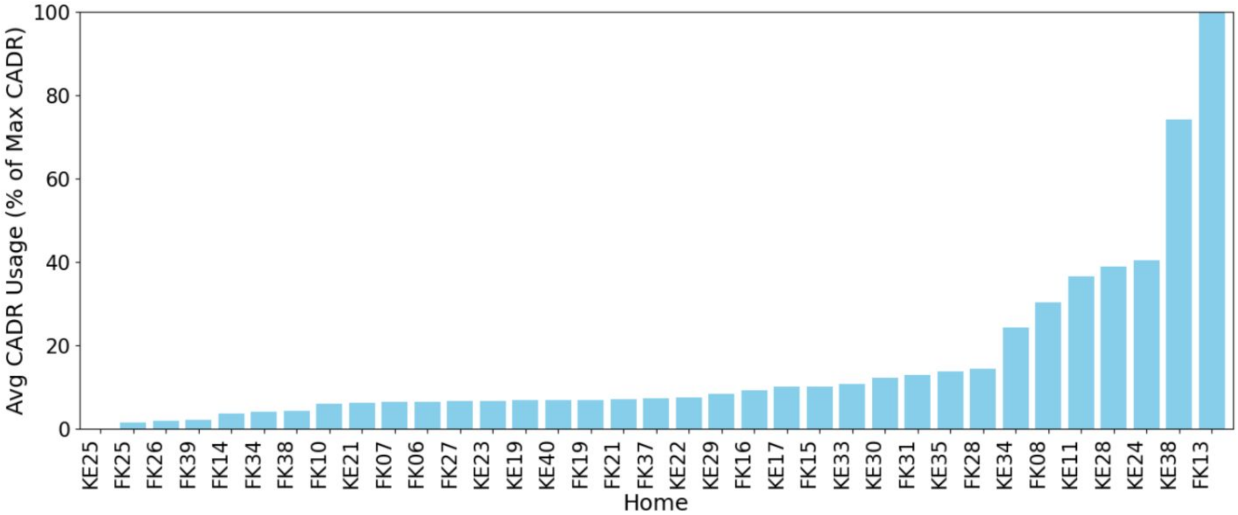

Figure S22. Percentage of maximum PAC CADR delivered in-situ in each home, average of post-intervention period

The distributions of average in-situ CADR delivered in PAC only homes and PAC+EC filter homes were similar (**Figure S23**), with a median of ~28 m^3/h and ~27 m^3/h, respectively. In-situ delivered CADRs were skewed higher in PAC only homes, with a mean of ~60 m^3/h compared to a mean of ~39 m^3/h in PAC+EC filter homes. While differences among the intervention groups were statistically significant ($p < 0.001$), the effect size was small ($d = 0.23$). Moreover, the difference favored PAC only homes, such that our observed differences in constrained I/O $PM_{2.5}$ ratios and F_{inf} in conditional analyses may be slightly conservative in favor of PAC only homes.

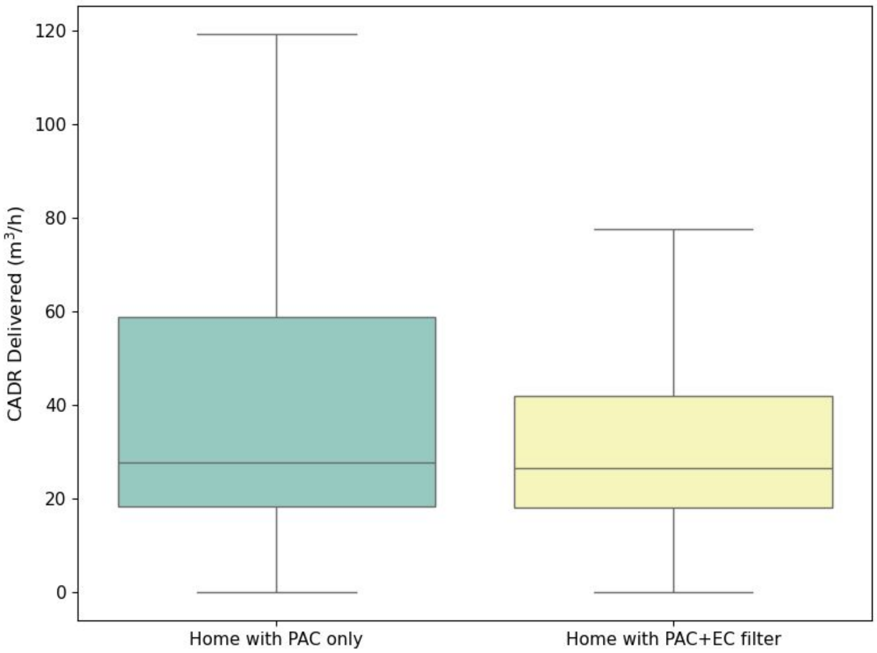

Figure S23. Distributions of average in-situ CADR delivered in PAC only and PAC+EC filter homes

To compare the impacts of PACs on mitigating ambient $PM_{2.5}$ infiltration, separate from EC usage, **Figure S24** compares constrained I/O $PM_{2.5}$ ratios between periods when PACs were operating and when PACs were off, comparing only data measured during times when ECs were measured or predicted to be off to minimize the influence of EC operation and the impact of DIY EC filters, yet comparing between homes with both PAC and EC filters and homes with only PACs. When PACs were operating and ECs were off (or likely off), median constrained I/O $PM_{2.5}$ ratios decreased from 0.78 to 0.52 (-33%; $p < 0.05$; $d = 0.74$) in PAC only homes and decreased from 0.74 to 0.53 (-28%; $p < 0.05$; $d = 0.82$) in homes with both PAC and EC filters. The constrained I/O $PM_{2.5}$ ratios with ECs off (or likely off) were similar across PAC only and PAC+EC filter groups both when PACs were on and off, suggesting that the home intervention groups were reasonably well randomized in terms of their base level of infiltration and also their PAC usage and impact of PACs on ambient $PM_{2.5}$ infiltration. **Table S21** summarizes the data in **Figure S24** in tabular form.

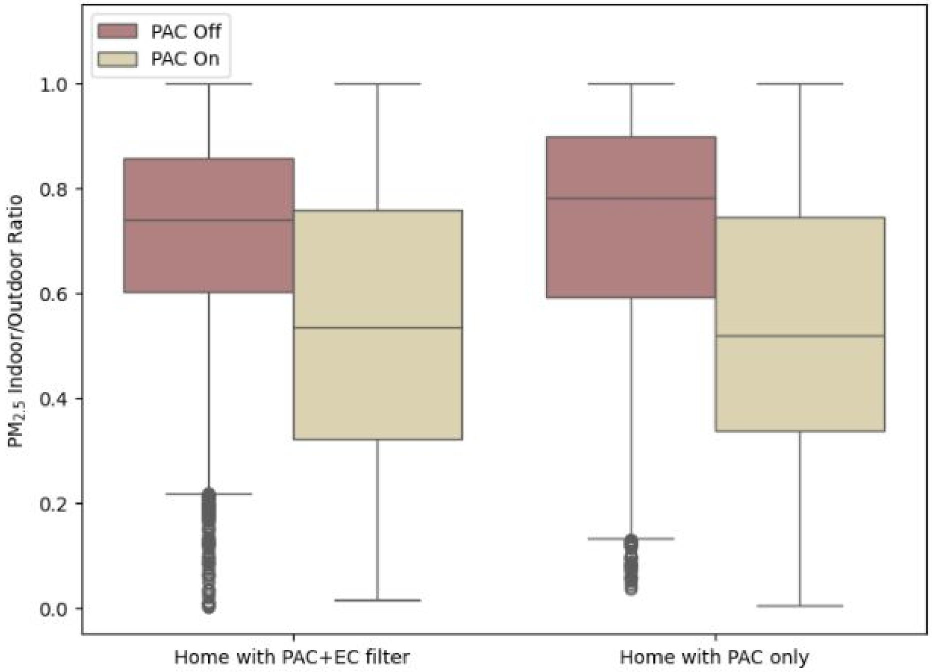

Figure S24. Comparison of constrained I/O $PM_{2.5}$ ratios between times when PACs were on vs. off, ECs were known or predicted to be off, comparing homes with PAC + EC filters to homes with PAC only

Table S21. Summary of constrained I/O $PM_{2.5}$ ratios during times when PACs were on vs. off, ECs were known or predicted to be off, comparing homes with PAC + EC filters to homes with PAC only (supporting Figure S24)

Group	PAC Run Mode	Count	Mean	SD	Min	25%	50%	75%	Max
PAC only	PAC Off	11517	0.73	0.21	0.04	0.59	0.78	0.90	1.00
	PAC On	62110	0.54	0.25	0.01	0.34	0.52	0.75	1.00
PAC+EC filter	PAC Off	8070	0.71	0.20	0.00	0.60	0.74	0.86	1.00
	PAC On	25906	0.54	0.25	0.02	0.32	0.53	0.76	1.00

EC runtime trends

Figure S25 shows the daily average EC runtime measured in individual homes that had PLLs on their ECs, as well as the average daily EC runtime across all homes with data available, along with the average daily outdoor temperature during the field measurements in 2023 (including both pre- and post-intervention periods). The daily average EC runtime was measured to range from ~40% early in the study (April), peaking at ~70% in July, and declining to <20% in late September. Although EC runtime varied between individual homes, the average daily EC runtime closely tracked average daily outdoor temperatures (Spearman rho = 0.57; $p < 0.001$).

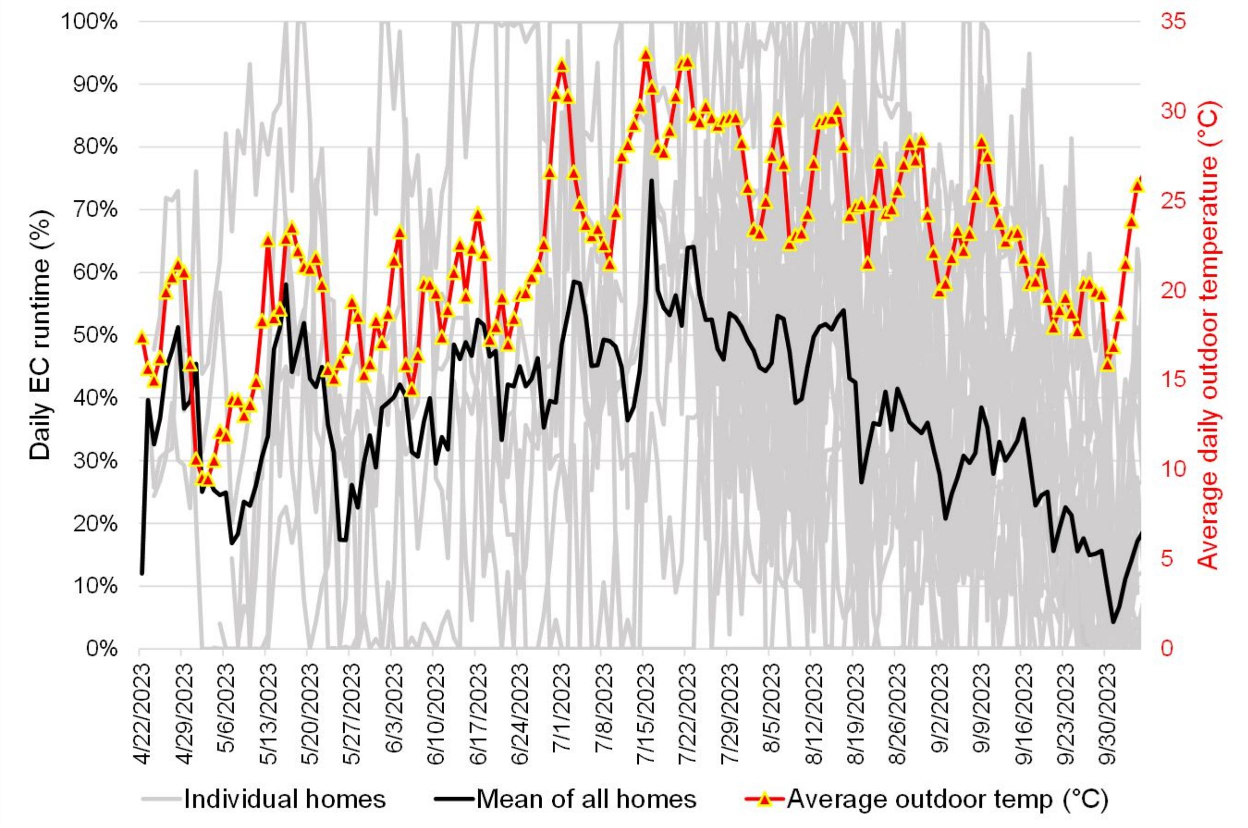

Figure S25. Daily average EC runtime across all homes (individual and average of homes) along with average daily outdoor temperature in the region

Supplemental References

(1) Kearney, J.; Wallace, L.; MacNeill, M.; Xu, X.; VanRyswyk, K.; You, H.; Kulka, R.; Wheeler, A. J. Residential Indoor and Outdoor Ultrafine Particles in Windsor, Ontario. *Atmos. Environ.* **2011**, *45* (40), 7583–7593. https://doi.org/10.1016/j.atmosenv.2010.11.002.

(2) MacNeill, M.; Wallace, L.; Kearney, J.; Allen, R. W.; Van Ryswyk, K.; Judek, S.; Xu, X.; Wheeler, A. Factors Influencing Variability in the Infiltration of PM2.5 Mass and Its Components. *Atmos. Environ.* **2012**, *61*, 518–532. https://doi.org/10.1016/j.atmosenv.2012.07.005.

(3) Kearney, J.; Wallace, L.; MacNeill, M.; Héroux, M.-E.; Kindzierski, W.; Wheeler, A. Residential Infiltration of Fine and Ultrafine Particles in Edmonton. *Atmos. Environ.* **2014**, *94*, 793–805. https://doi.org/10.1016/j.atmosenv.2014.05.020.

(4) MacNeill, M.; Kearney, J.; Wallace, L.; Gibson, M.; Héroux, M. E.; Kuchta, J.; Guernsey, J. R.; Wheeler, A. J. Quantifying the Contribution of Ambient and Indoor-Generated Fine Particles to Indoor Air in Residential Environments. *Indoor Air* **2014**, *24* (4), 362–375. https://doi.org/10.1111/ina.12084.

(5) Lunderberg, D. M.; Liang, Y.; Singer, B. C.; Apte, J. S.; Nazaroff, W. W.; Goldstein, A. H. Assessing Residential PM $_{2.5}$ Concentrations and Infiltration Factors with High Spatiotemporal Resolution Using Crowdsourced Sensors. *Proc. Natl. Acad. Sci.* **2023**, *120* (50), e2308832120. https://doi.org/10.1073/pnas.2308832120.

SI Appendix: Data Tables

Full data tables with basic information on each participating home, received intervention, and any spot measurements are provided in this SI appendix.

Table A1. Home and HVAC characteristics in homes that were received PAC+EC filters (25 homes)

Home #	Home Type[2]	Evaporative Cooler Make/Model	Outdoor EC Type[3]	Portable Air Cleaner (PAC) Make/Model	Outdoor Purple Air Monitor distance (km)[4]	Window AC	Range Hood Type (x for all that apply)				
							No Hood or non-operable	Over the Range	Exhausts to Outdoor	Recirculate inside	Other
**FK03**	Detached SF	Climate EC	Side	Winix D360	0 (FK 10)[4]	No		x	x		
**FK06**	Manufactured home	Bonaire	Side	Levoit H133	0 (FK 10)[4]	Yes		x	x		
**FK08**	Manufactured home	Bonaire 6280035	Side	Levoit 300	0 (FK 10)[4]	No	x				
**FK10**	Manufactured home	Mastercool MCP44	Side	Winix D360	0 (FK 10)[4]	Yes		x		x	
**FK14**	Manufactured home	Bonaire (2 ECs)	Side	Winix D360	0 (FK 10)[4]	Yes (2)		x	x		
**FK15**	n/a[1]	n/a[1]	n/a[1]	Levoit H133	0 (FK 10)[4]	n/a[1]	n/a[1]	n/a[1]	n/a[1]	n/a[1]	n/a[1]
**FK16**	Manufactured home	Phoenix S63NTKCJ	Side	Winix D360	0 (FK 10)[4]	Yes			x		
**FK19**	Manufactured home	Champion EC	n/a[1]	Winix D360	0 (FK 12)[4]	Yes		x			
**FK20**	Manufactured home	Champion EC	Side	Winix D360	0 (FK 12)[4]	No	x				
**FK21**	Manufactured home	Champion EC	Side	Winix D360	0 (FK 12)[4]	No		x	x		
**FK26**	Manufactured home	Phoenix HE2913	Side	Winix D360	1.1 (FK 10)[4]	Yes		x	x		
**FK37**	n/a[1]	n/a[1]	n/a[1]	Winix D360	0 (FK 10)[4]	n/a[1]	n/a[1]	n/a[1]	n/a[1]	n/a[1]	n/a[1]
**FK38**	n/a[1]	n/a[1]	n/a[1]	Winix D360	0 (FK 10)[4]	n/a[1]	n/a[1]	n/a[1]	n/a[1]	n/a[1]	n/a[1]
**FK39**	Manufactured home	Phoenix HE2913A	Side	Winix D360	0.5 (FK 36)[4]	Yes		x		x	
**KE11**	Manufactured home	Brisa BW4000	Side	Levoit 300	1.1 (KE 29)[4]	No	x				
**KE19**	Manufactured home	Champion RN35W	Side	Winix D360	1.1 (KE 29)[4]	No		x		x	
**KE21**	Detached SF	Brisa BW4502	Side	Winix D360	0.6 (KE 29)[4]	No		x	x		
**KE22**	Detached single-family	Champion EC	Side	Winix D360	0 (KE 22)[4]	No					x

**KE23**	Manufactured home	Champion ECM28	Side	Winix D360	0.3 (KE 30)[4]	No		x	x		
**KE24**	Manufactured home	Powercool UL7000	Side	Levoit 300	0.3 (KE 30)[4]	Yes					x
**KE25**	Manufactured home	Brisa BW4502	Side	Winix D360	0.3 (KE 30)[4]	No		x	x		
**KE33**	Multi-unit building	Champion RN35W	Side	Winix D360	0.6 (KE 29)[4]	Yes		x	x		
**KE36**	Detached SF	n/a[1]	Side	Winix D360	1.5 (KE 30)[4]	No	x				
**KE37**	Manufactured home	n/a[1]	Side	Winix D360	9.8 (KE 30)[4]	No		x	x		
**KE39**	Detached SF	Phoenix HE2913 (2 ECs)	Side	Winix D360	1.1 (KE 30)[4]	Yes			x		

[1] n/a: information not available from the field team or not measured; [2] SF: single-family; [3] side: side-mounted evaporative cooler (EC) (e.g., through window or wall)
[4] Home at which nearest outdoor Purple Air monitor is located; Zeros (0 km) distance indicate that the outdoor monitor is within the same mobile/trailer home park

Table A2. Home and HVAC characteristics in homes that received PAC only (23 homes)

Home #	Home Type[2]	Evaporative Cooler Make/Model	Outdoor EC Type[3]	Portable Air Cleaner (PAC) Make/Model	Outdoor Purple Air Monitor distance (km) [4]	Window AC	Range Hood Type (x for all that apply)				
							No Hood or non-operable	Over the Range	Exhausts to Outdoor	Recirculate inside	Other
**FK02**	Manufactured home	Phoenix HE2911SE	Side	n/a[1]	0 (FK 10) [4]	No		x		x	
**FK07**	Manufactured home	Tradewinds EC	RM[3]	Winix D360	0 (FK 10) [4]	No					x
**FK12**	Detached SF	n/a[1]	Side	Winix D360	0 (FK 12) [4]	No		x	x		
**FK13**	Detached SF	Breezeair	Roof	Levoit H133	2.4 (FK 10) [4]	No		x	x		
**FK16**	n/a[1]	n/a[1]	n/a[1]	Winix D360	0 (FK 10) [4]	n/a[1]	n/a[1]	n/a[1]	n/a[1]	n/a[1]	n/a[1]
**FK19**	n/a[1]	n/a[1]	n/a[1]	Winix D360	0 (FK 10) [4]	n/a[1]	n/a[1]	n/a[1]	n/a[1]	n/a[1]	n/a[1]
**FK25**	Manufactured home	Champion Cooler	Roof	Levoit 300	1.1 (FK 10) [4]	Yes	x				
**FK27**	Detached SF	Frigiking FD450A	Roof	Winix D360	7.9 (FK 10) [4]	No		x	x		
**FK28**	Manufactured home	n/a[1]	n/a[1]	Winix D360	1.6 (FK 10) [4]	No					x
**FK29**	Manufactured home	Champion EC	n/a[1]	Winix D360	14.5 (FK 10) [4]	Yes		x	x		
**FK31**	Manufactured home	Champion 4401DD	n/a[1]	Levoit H133	0 (FK 10) [4]	Yes		x		x	
**FK32**	Detached SF	Tradewinds	Roof	Winix D360	2.4 (FK 10) [4]	Yes					x
**FK34**	n/a[1]	n/a[1]	n/a[1]	Levoit H133	0 (FK 10) [4]	n/a[1]	n/a[1]	n/a[1]	n/a[1]	n/a[1]	n/a[1]
**FK36**	Detached SF	Bonaire	Side	Winix D360	0 (FK 36) [4]	Yes		x	x		
**KE10**	Manufactured home	Champion	Side	Winix D360	1.1 (KE 29) [4]	No		x		x	
**KE17**	Multi-unit building	n/a[1]	Side	Winix D360	0.7 (KE 29) [4]	No	x				
**KE28**	Detached SF	Mastercool	Side	Winix D360	1.6 (KE 30) [4]	Yes		x	x		
**KE29**	Detached SF	Champion EC	Side	Winix D360	0 (KE 29) [4]	No		x		x	
**KE30**	Detached SF	Champion 5500dd	Side	Levoit H133	0 (KE 30) [4]	Yes (2)		x	x		
**KE34**	Detached SF	Scone RC50WA	Side	Winix D360	0.6 (KE 30) [4]	Yes		x	x		
**KE35**	Detached SF	Champion CM28	Side	Winix D360	1 (KE 30) [4]	Yes		x	x		
**KE38**	Manufactured home	n/a[1]	Side	Levoit 300	0.6 (KE 30) [4]	No	x				
**KE40**	Manufactured home	n/a[1]	n/a[1]	Winix D360	7.4 (KE 22) [4]	No	x				

[1] n/a: information not available from the field team or not measured; [2] SF: single-family; [3] side or roof: side- or roof-mounted evaporative cooler (EC)

[4] Home at which nearest outdoor Purple Air monitor is located; Zeros (0 km) distance indicate that the outdoor monitor is within the same mobile/trailer home park

Table A3. Spot measurements in homes that received EC filters, at time of EC filter installation (July 2023)

Home #	DIY solution type[2]	EC flowrate (no EC filter) (CFM)	Flowrate (w/ EC filter) (CFM)	% Change in EC flowrate	EC power draw (no EC filter) (Watts)	EC Power (w/ EC filter) (Watts)	Envelope pressure differential (no EC filter) (Pa)	Envelope pressure differential (w/ EC filter) (Pa)
**FK03**	n/a[1]	1060	866	-18%	95	96	27.7	19.6
**FK06**	4 of SM13 & SF	2653	2264	-15%	386	379	30.7	18.7
**FK08**	2 of LM13	1682	1318	-22%	365	376	13.1	7.6
**FK10**	2 of LM13	1968	1650	-16%	297	306	36.6	26.8
**FK14 (EC1)**	2 LM13 & SF	2234	1630	-27%	318	330	12.5	6.6
**FK14 (EC2)**	2 of LM13 & SF	1322	1293	-2%	440	445	4.1	2.6
**FK15**	2 of LM13	2377	2022	1-5%	382	390	6.6	4.8
**FK20**	4 of SM13	1931	1693	1-2%	415	390	10	7.5
**FK21**	n/a[1]	n/a[1]	n/a[1]	n/a[1]	n/a[1]	n/a[1]	n/a[1]	n/a[1]
**FK26**	n/a[1]	2110	1625	-23%	287	278	2.1	1.9
**FK37**	6 of SM13	1065	960	-10%	258	250	10	7.5
**FK38 (EC1)**	n/a[1]	1381	1030	-25%	n/a[1]	n/a[1]	n/a[1]	n/a[1]
**FK38 (EC2)**	n/a[1]	1783	1284	-28%	n/a[1]	n/a[1]	n/a[1]	n/a[1]
**FK39**	n/a[1]	1467	1399	-5%	n/a[1]	n/a[1]	n/a[1]	n/a[1]
**KE11**	3 of LM13	2423	2028	16	435	400	18.8	13.6
**KE19**	4 of SM13	1482	1321	11	360	355	2.8	2.5
**KE21**	6 of LM13	1717	1597	7	435	427	44.2	36.8
**KE22**	SF	913	648	29	248	237	4.7	3.5
**KE24**	6 of LM13	1330	1218	8	286	277	7.3	5.6
**KE33**	4 of SM13	1343	1175	13	306	297	18.9	15.9
**KE36**	SF	1090	579	47	212	214	9.2	2.6
**KE37**	3 of LM13 & SF	1095	862	21	175	177	17.5	10.1
**KE39**	n/a[1]	836	705	16	247	350	5.6	4.5

[1] n/a: information not available from the field team or not measured

[2] EC filters installed: SM13 - small MERV 13 (16"x25"x4"); LM13 - large MERV 13 (20"x30"x4"); SF - Sheet Filter (0.5-cm thick flat sheet media, labeled as MERV 13-16, lab-tested closer to MERV 11)

Table A4. Spot measurements in homes that received EC filters, end of study (October 2023)

Home #	DIY solution type[2]	EC flowrate (no EC filter) (CFM)	Flowrate (w/ EC filter) (CFM)	% Change in EC flowrate	EC power draw (no EC filter) (Watts)	EC Power (w/ EC filter) (Watts)	Envelope pressure differential (no EC filter) (Pa)	Envelope pressure differential (w/ EC filter) (Pa)
**FK03**	n/a[1]	1022	990	3	253	253	n/a[1]	n/a[1]
**FK06**	4 of SM13 & SF	2437	1870	23	400		n/a[1]	n/a[1]
**FK08**	2 of LM13	1065	836	21	191	196	n/a[1]	n/a[1]
**FK10**	2 of LM13	1438	780	46	241	261	n/a[1]	n/a[1]
**FK14 (EC1)**	2 LM13 & SF	n/a[1]	n/a[1]	n/a[1]	n/a[1]	n/a[1]	n/a[1]	n/a[1]
**FK14 (EC2)**	2 of LM13 & SF	1081	1051	3	308	313	n/a[1]	n/a[1]
**FK15**	2 of LM13	2231	1748	22	n/a	n/a	n/a[1]	n/a[1]
**FK20**	4 of SM13	1537	1184	23	408	371	n/a[1]	n/a[1]
**FK21**	n/a[1]	1288	1081	16	278	n/a	n/a[1]	n/a[1]
**FK26**	n/a[1]	1356	1101	19	276	274	n/a[1]	n/a[1]
**FK37**	6 of SM13	879	698	21	247	243	n/a[1]	n/a[1]
**FK38 (EC1)**	n/a[1]	n/a[1]	n/a[1]	n/a[1]	n/a[1]	n/a[1]	n/a[1]	n/a[1]
**FK38 (EC2)**	n/a[1]	2281	1989	13	n/a	n/a	n/a[1]	n/a[1]
**FK39**	n/a[1]	1722	1423	17	13.3	13.3	n/a[1]	n/a[1]
**KE11**	3 of LM13	n/a[1]	n/a[1]	n/a[1]	n/a[1]	n/a[1]	n/a[1]	n/a[1]
**KE19**	4 of SM13	762	616	19	n/a[1]	n/a[1]	n/a[1]	n/a[1]
**KE21**	6 of LM13	2062	1853	10	500	473	13.9	12.7
**KE22**	SF	n/a[1]	n/a[1]	n/a[1]	n/a[1]	n/a[1]	n/a[1]	n/a[1]
**KE23**	n/a[1]	877	783	11	154	178	4.7	3.5
**KE24**	6 of LM13	1377	1281	7	n/a[1]	n/a[1]	n/a[1]	n/a[1]
**KE25**	n/a[1]	1383	1248	10	338	330	1.8	7.1
**KE33**	4 of SM13	1134	939	17	338	330	24.1	18.3
**KE36**	SF	n/a[1]	n/a[1]	n/a[1]	n/a[1]	n/a[1]	n/a[1]	n/a[1]
**KE37**	3 of LM13 & SF	1372	1210	12	152	152	n/a[1]	n/a[1]
**KE39**	n/a[1]	334	389	-16	n/a[1]	n/a[1]	5.6	4.5
**KE41**	n/a[1]	1993	1788	10	615	720	12.1	11.2

[1] n/a: information not available from the field team or not measured

[2] EC filters installed: SM13 - small MERV 13 (16”x25”x4”); LM13 - large MERV 13 (20”x30”x4”); SF - Sheet Filter (0.5-cm thick flat sheet media, labeled as MERV 13-16, lab-tested closer to MERV 11)

SI Appendix: Photo Gallery - DIY filter installations in the field sites

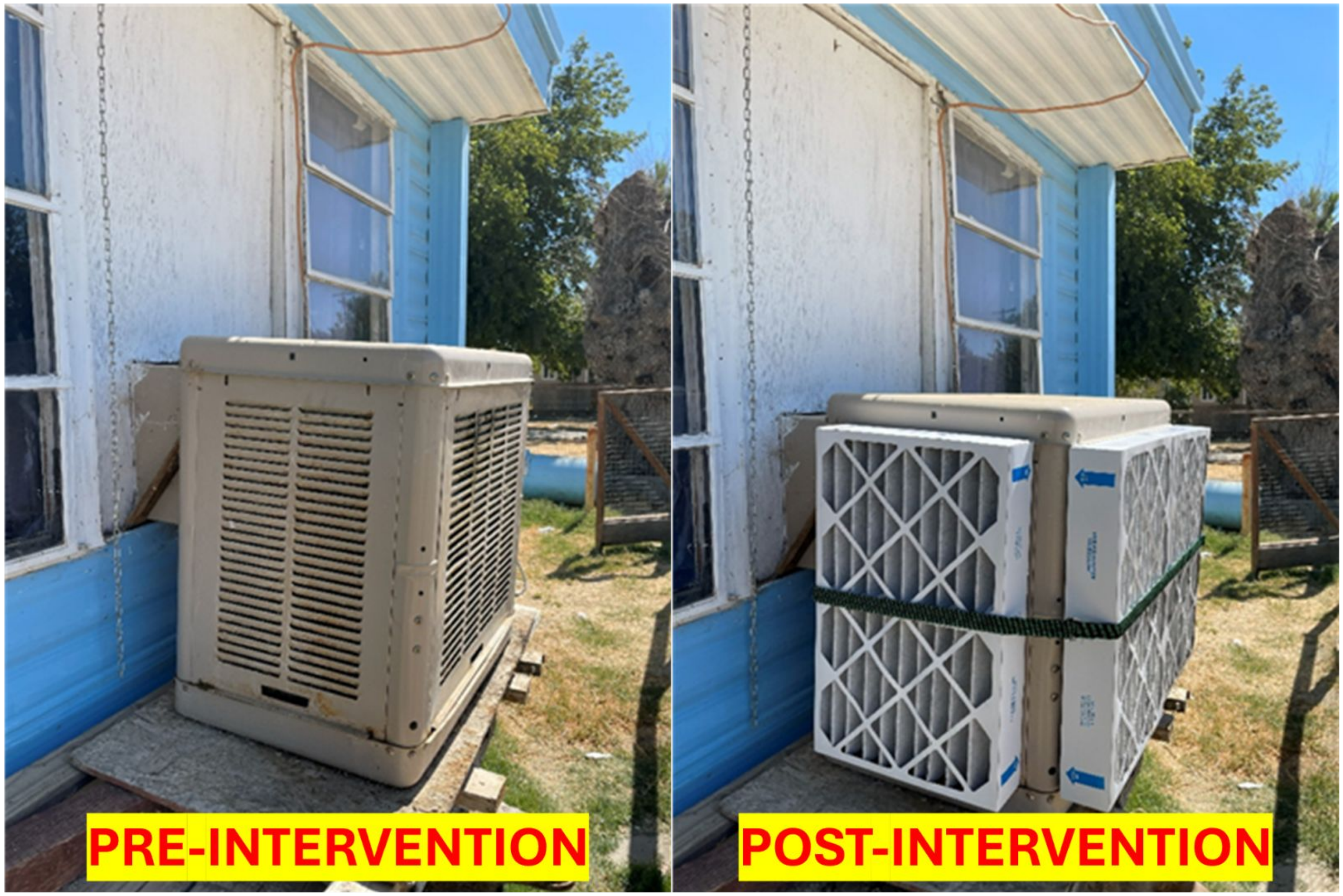

Figure A1. Regular DIY Solution – Filter dimensions conform closely to EC intakes with a bungee cord

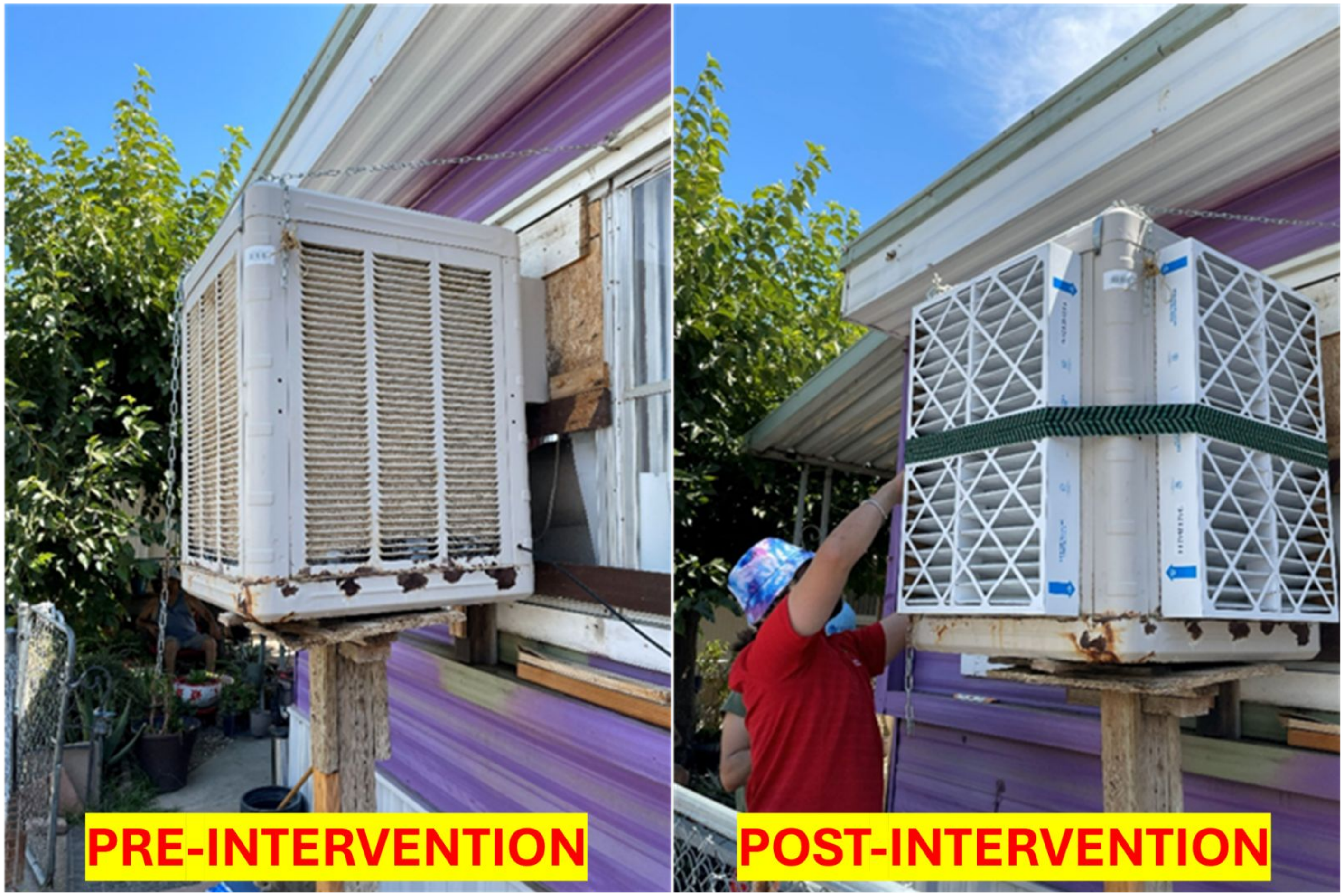

Figure A2. Regular DIY Solution - Filter dimensions conform closely to EC intakes with a bungee cord

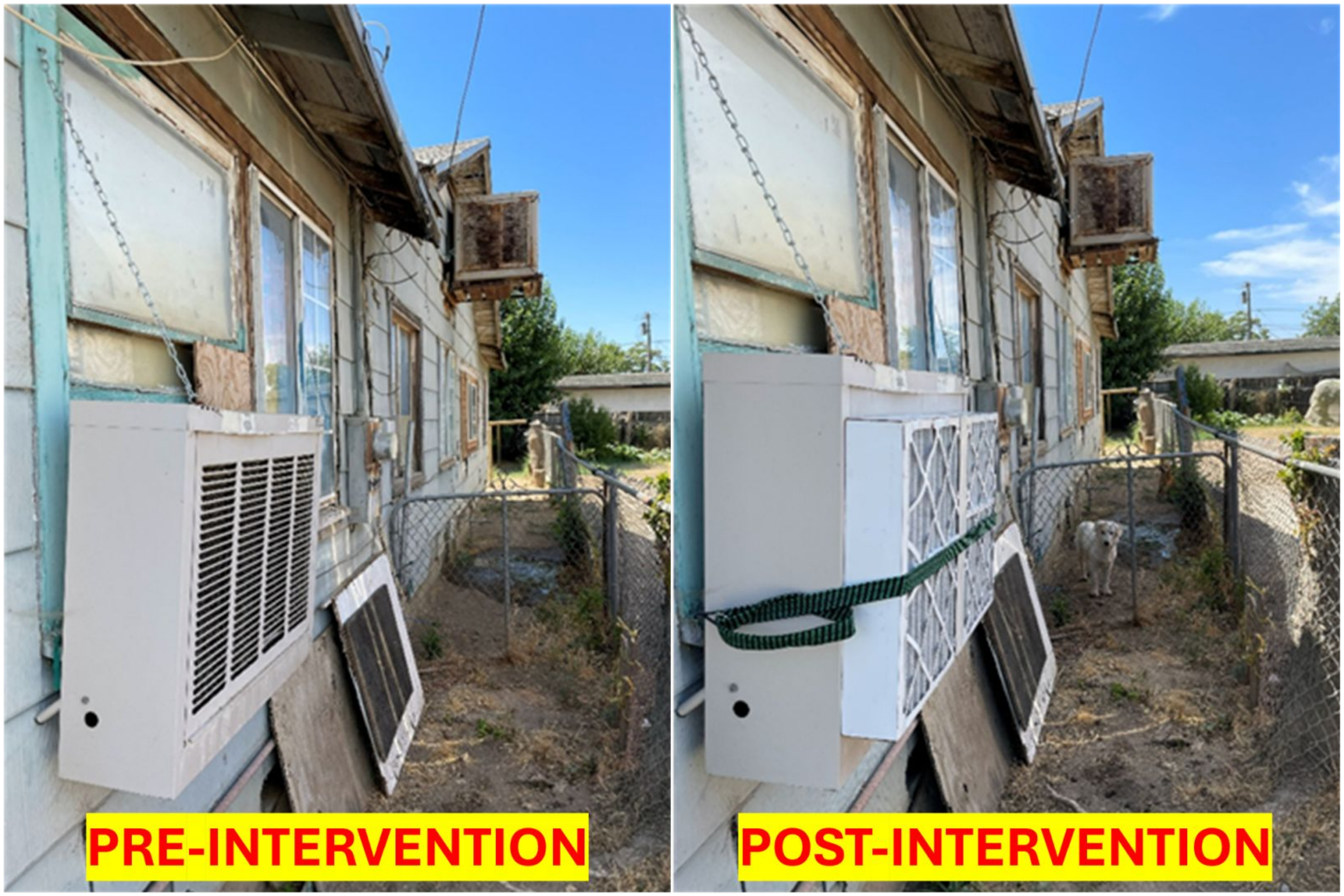

Figure A3. Regular DIY Solution - Filter dimensions conform closely to EC intakes with a bungee cord

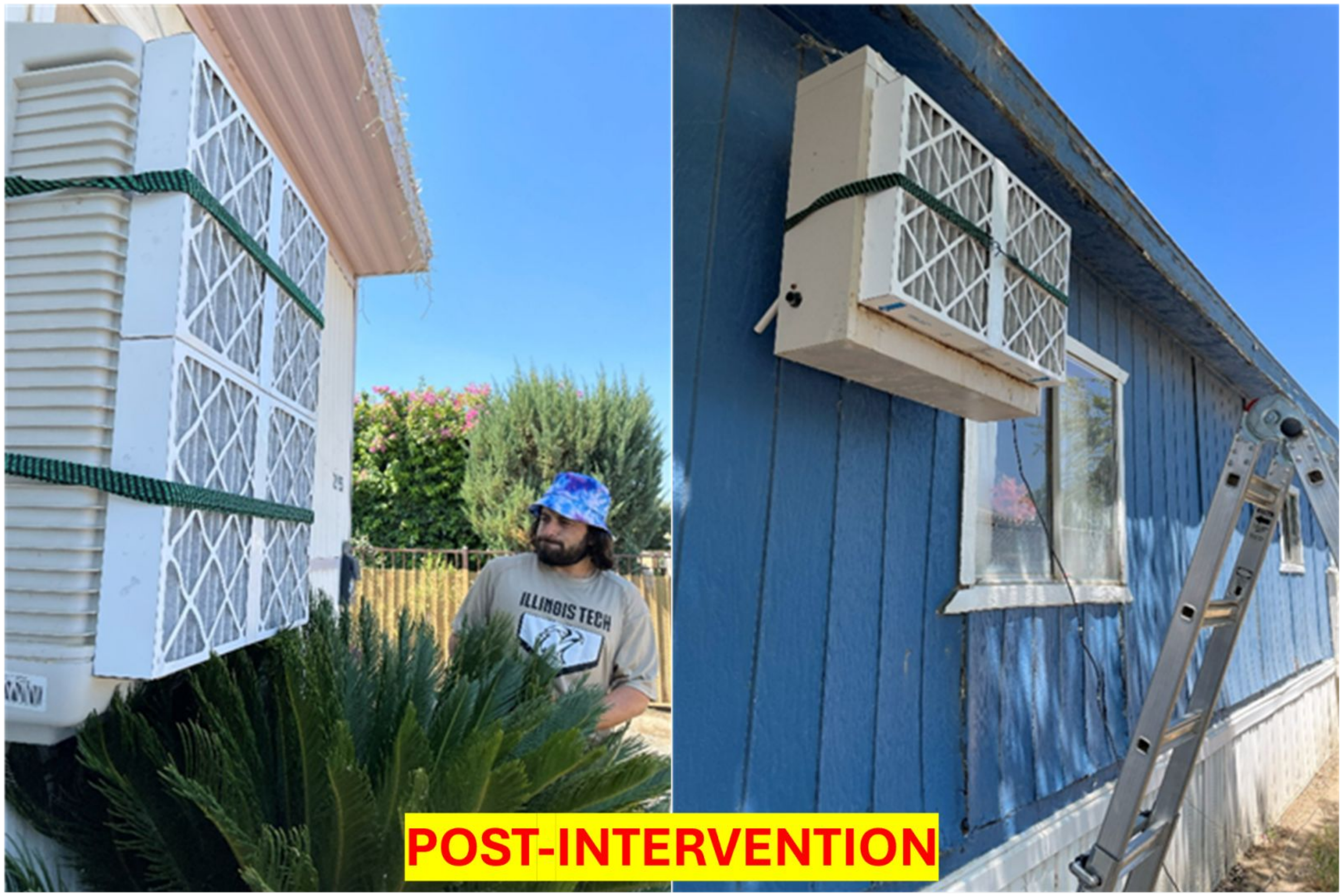

Figure A4. Regular DIY Solution - Filters on EC held together with a bungee cord but with some difficulties during installation due to surrounding obstacles

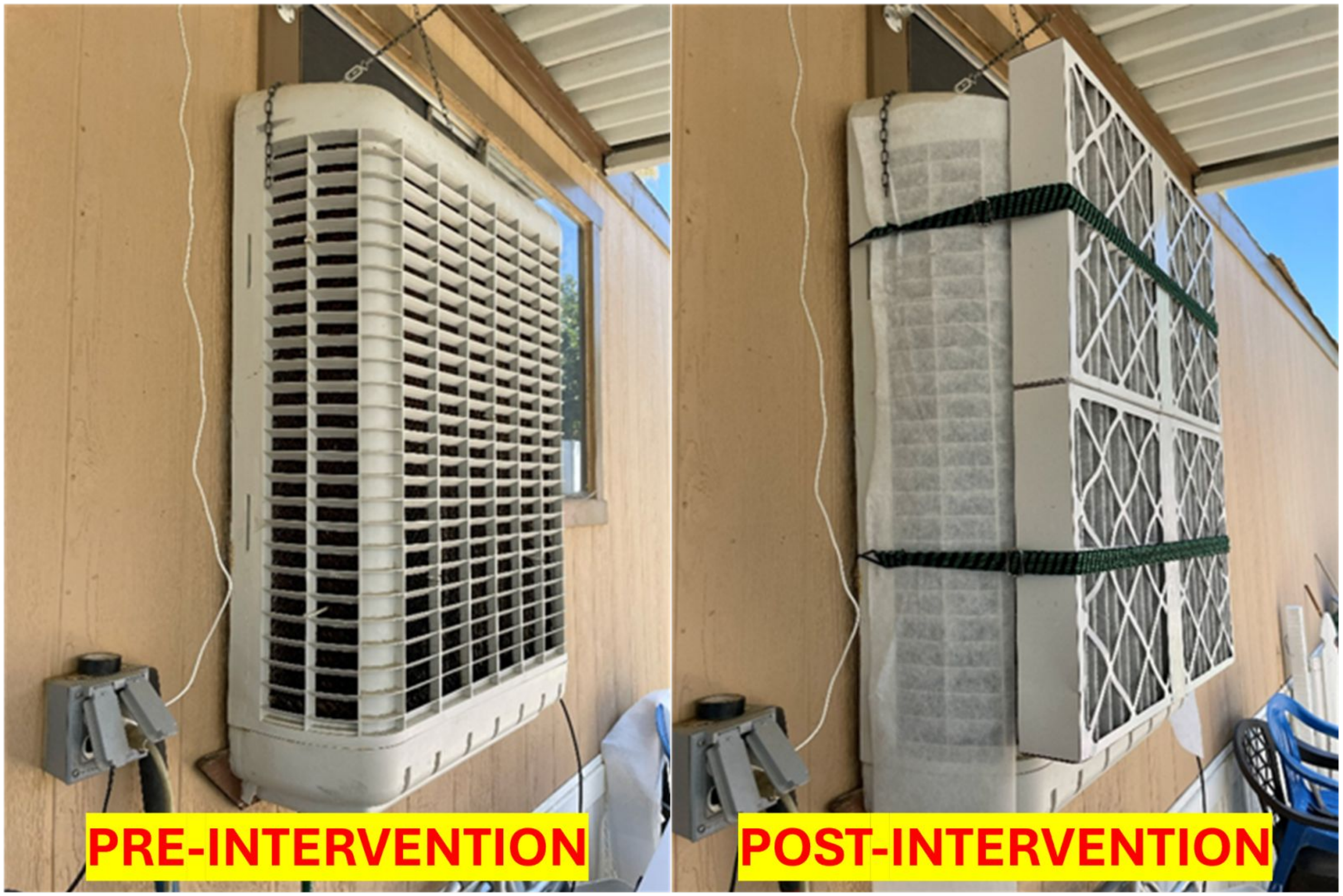

Figure A5. Combo DIY Solution – Regular Filters on EC combined with sheet filters due to EC shape irregularities and obstacles

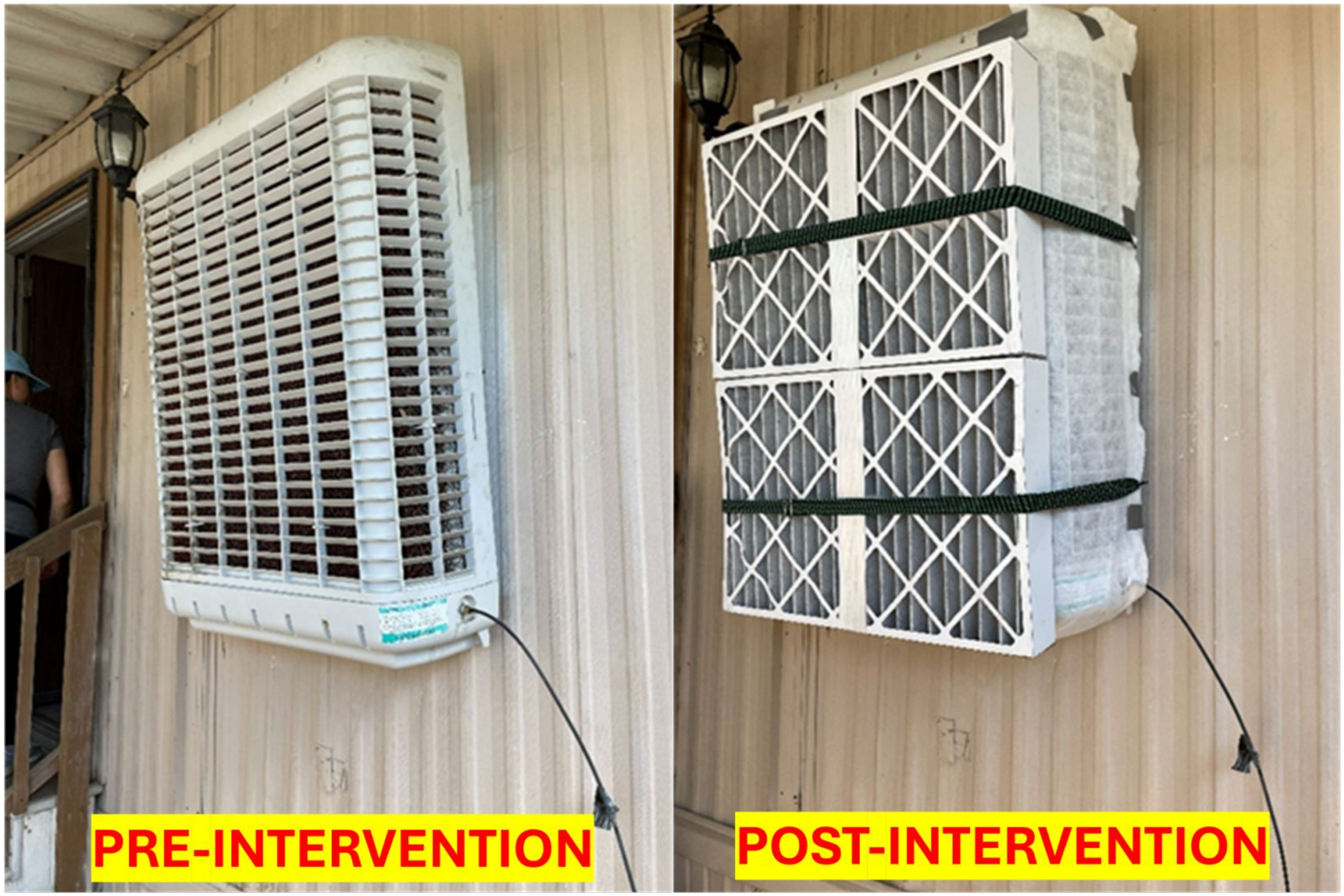

Figure A6. Combo DIY Solution – Regular Filters on EC combined with sheet filters due to EC shape irregularities and obstacles

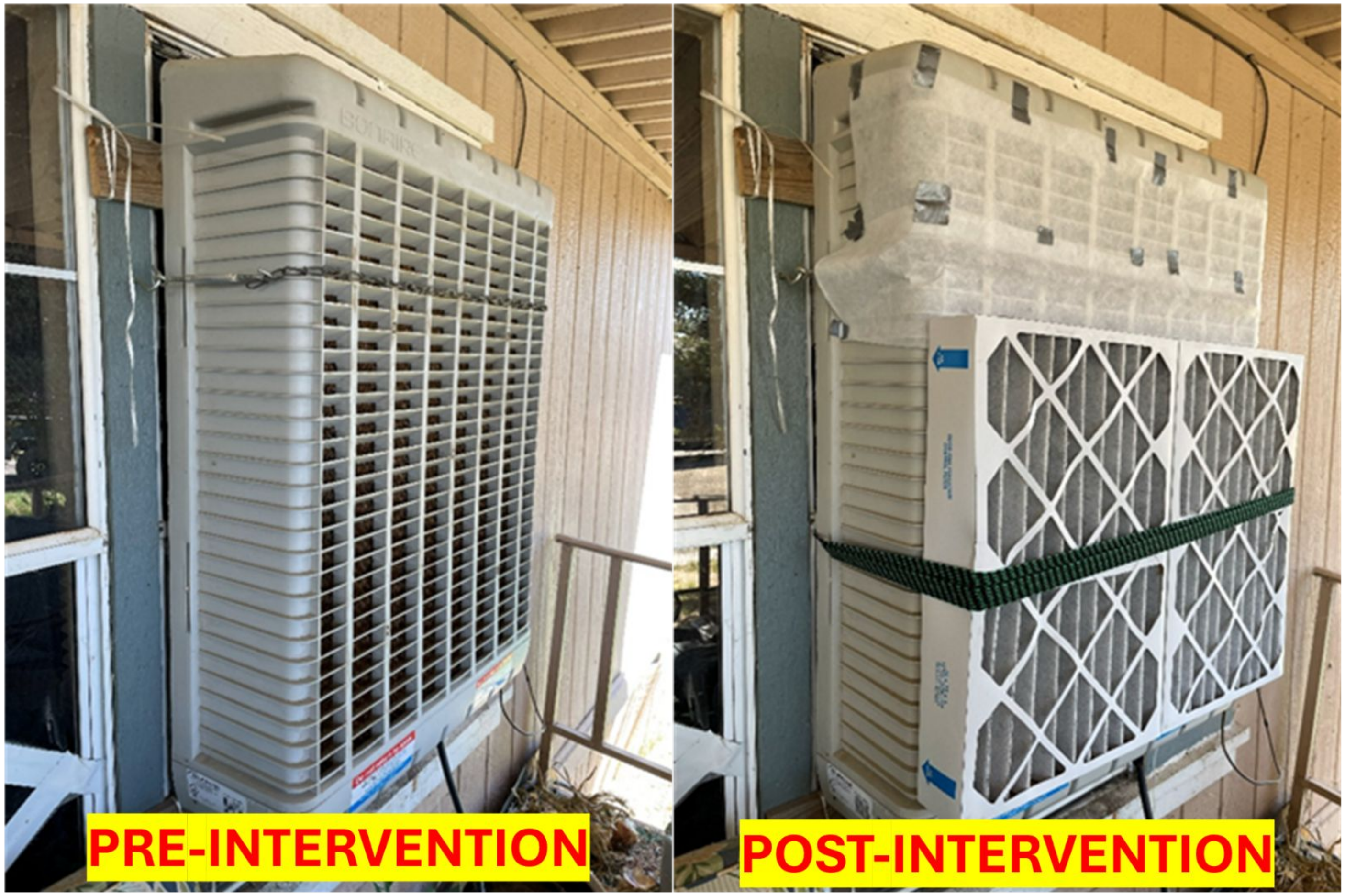

Figure A7. Combo DIY Solution – Regular Filters on EC combined with sheet filters due to EC shape irregularities and obstacles

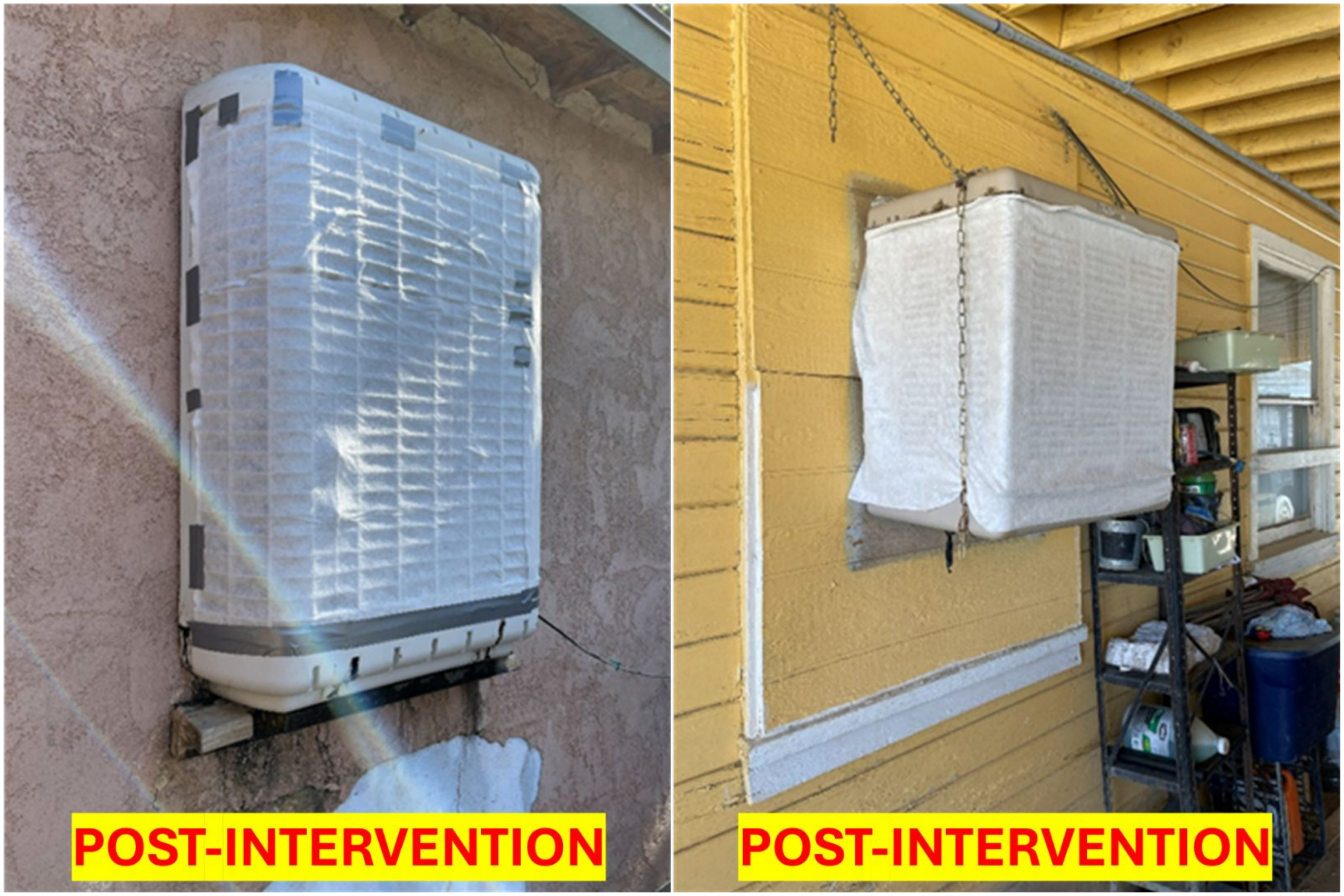

Figure A8. Sheet Filter Solution – Only Sheet filters on EC due to EC shape irregularities and obstacles preventing attachment of regular filters
